# Interplay between the Directing Group and Multifunctional Acetate Ligand in Pd-Catalyzed *anti*-Acetoxylation of Unsymmetrical Dialkyl-Substituted Alkynes

**DOI:** 10.1021/acscatal.2c00710

**Published:** 2022-05-19

**Authors:** Javier Corpas, Enrique M. Arpa, Romain Lapierre, Inés Corral, Pablo Mauleón, Ramón Gómez Arrayás, Juan C. Carretero

**Affiliations:** † Departamento de Química Orgánica, Facultad de Ciencias, Universidad Autónoma de Madrid (UAM), 28049 Madrid, Spain; ‡ Division of Theoretical Chemistry, IFM, Linköping University, 581 83 Linköping, Sweden; § Departamento de Química, Facultad de Ciencias, UAM, Cantoblanco, 28049 Madrid, Spain; ∥ Institute for Advanced Research in Chemical Sciences (IAdChem), UAM, 28049 Madrid, Spain

**Keywords:** multifunctional ligand, ligand-assisted proton shuttle, acetoxylation of alkynes, unsymmetrical dialkyl alkynes, metal−ligand cooperativity, directing group

## Abstract

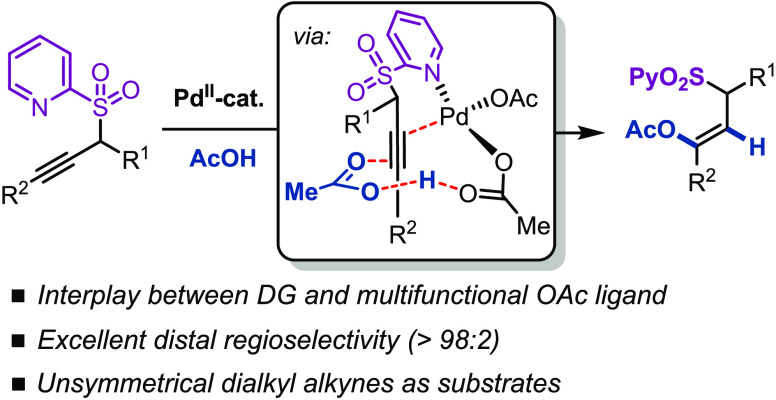

The cooperative action of the acetate ligand, the 2-pyridyl sulfonyl (SO_2_Py) directing group on the alkyne substrate, and the palladium catalyst has been shown to be crucial for controlling reactivity, regioselectivity, and stereoselectivity in the acetoxylation of unsymmetrical internal alkynes under mild reaction conditions. The corresponding alkenyl acetates were obtained in good yields with complete levels of β-regioselectivity and *anti*-acetoxypalladation stereocontrol. Experimental and computational analyses provide insight into the reasons behind this delicate interplay between the ligand, directing group, and the metal in the reaction mechanism. In fact, these studies unveil the multiple important roles of the acetate ligand in the coordination sphere at the Pd center: (i) it brings the acetic acid reagent into close proximity to the metal to allow the simultaneous activation of the alkyne and the acetic acid, (ii) it serves as an inner-sphere base while enhancing the nucleophilicity of the acid, and (iii) it acts as an intramolecular acid to facilitate protodemetalation and regeneration of the catalyst. Further insight into the origin of the observed regiocontrol is provided by the mapping of potential energy profiles and distortion–interaction analysis.

## Introduction

**Scheme 1:**
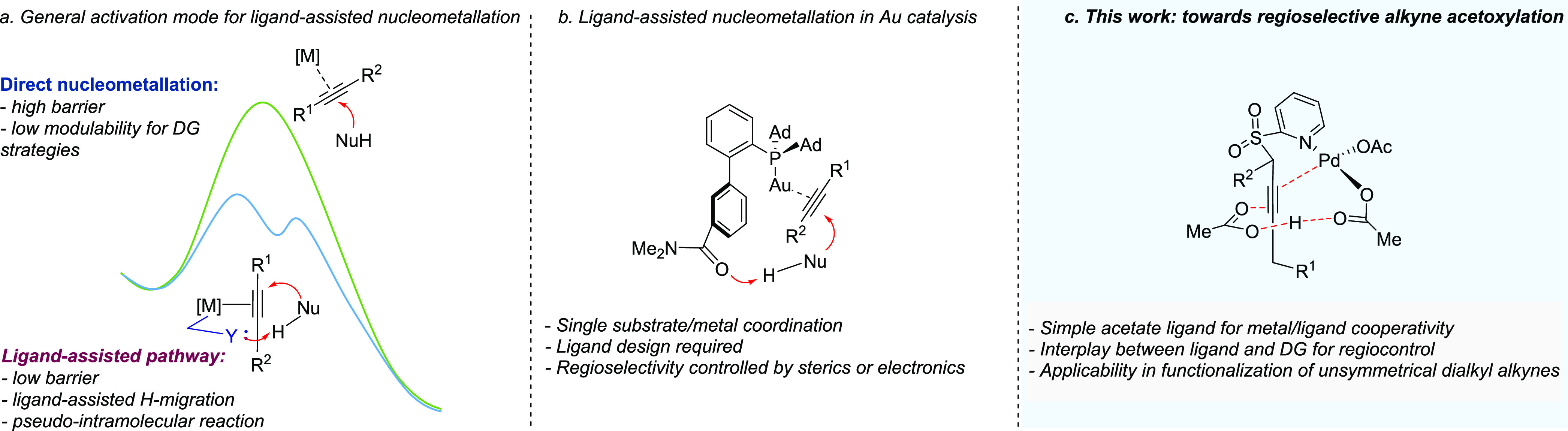
Catalytic *anti*-Hydro-oxycarbonylation of Alkynes via Metal/Ligand Cooperativity

Catalytic reactions controlled by the action of ligands playing active roles besides stabilizing and tuning the metal, commonly known as multifunctional ligands, are capturing increasing attention in synthetic chemistry.^1^ The ability of carboxylate ancillary ligands to switch denticity, thereby facilitating reactions alongside the metal, has been widely exploited in some catalytic transformations.^2^ For example, acetate ligands have enabled mechanisms for C–H activation alternative to direct oxidative addition by acting as an inner-sphere base to assist deprotonation of the C–H bond concomitant with M–C bond formation, which is referred to as concerted metalation–deprotonation (CMD) or amphiphilic metal–ligand activation (AMLA).^3^ Another general class of related mechanism refers to proton transfer between two different ligands without involving any metal hydride known as ligand-to-ligand hydrogen transfer (LLHT).^4^ However, in this type of reaction, most often the protonated ligand subsequently leaves the metal to afford the product. Alternatively, it could remain bonded to the metal and still has active participation as an intramolecular acid at other stages of the catalytic cycle, a mechanistic variation termed as a ligand-assisted proton shuttle (LAPS).^5^ This strategy is neither restricted to carboxylate ligands nor to C–H activation. Capitalizing on this concept, the application of metal–ligand interaction in the catalytic functionalization of alkynes would be translated into more efficient and selective transformations (Scheme 1a). Recently, Zhang and co-workers have described a gold-catalyzed *anti*-carboxylation of alkynes using an amide-containing phosphine ligand, which directs the attack of the incoming carboxylic acid via hydrogen bonding.^6^ This ligand–reagent interaction not only makes the addition reaction pseudointramolecular in nature but also enhances the nucleophilicity of the acid (Scheme 1b). In addition, the protonated amide that remains at the coordination sphere of the metal subsequently serves as an intramolecular proton source for protodeauration, thereby accelerating the catalyst turnover.^7^ Despite some emerging examples, reactions that occur via a LAPS mechanism remain rare.^8^

Despite the wealth of reactivity that has been established in the catalytic functionalization of alkynes toward the construction of stereochemically defined olefins,^9−11^ unsymmetrical dialkyl-substituted alkynes are noticeably absent from most contributions and, when present, typically provide unsatisfactory levels of reactivity and/or regioselectivity.^12^ In an effort to enhance the involvement of this class of alkynes, our group has reported an indirect solution that relies on the use of a propargylic SO_2_Py directing group for achieving site-selectivity control.^13^ In our previously reported Pd-catalyzed hydroarylation reaction, it was found that monodentate phosphine ligands significantly outperformed their bidentate analogues because they allow the alkyne substrate to accommodate through bidentate coordination in the ligand sphere of the metal, facilitating the regioselective insertion of the Pd–Ar σ-bond.^13c^ Guided by this knowledge, we envisaged that Pd-catalyzed acetoxylation could benefit from the readiness of carboxylate ligands to switch their denticity, thus generating a coordination position on the metal center for substrate activation. This transformation, also known as hydro-oxycarbonylation, is typically proposed to occur via an *anti*-carboxymetalation pathway in which the carboxylic acid attacks the C–C triple bond coordinated to an alkynophilic metal.^14−18^ To the best of our knowledge, only two single isolated examples of fully regioselective carboxylation of unsymmetrical dialkyl alkynes have been disclosed.^19^ Recently, Li has reported the use of the 2-pyridyloxy moiety as a directing group under Ru catalysis, but the method is limited to aryl-containing alkynes.^19b^ On the other hand, examples of palladium catalysis in carboxylation of internal alkynes^16^ are scarce compared to ruthenium^14^ or gold catalysis,^17^ and the mechanism of the reaction has not been investigated in detail before. This paper describes the viability, as well as a combined experimental and computational mechanistic study, of the SO_2_Py-directed regiocontrolled *anti*-acetoxylation of unsymmetrical dialkyl alkynes. This study has revealed multiple roles of the acetate ligand throughout the catalytic cycle. These include creating an acetic acid-binding pocket close to the metal center to favor regioselective addition, enhancing the nucleophilicity of the acid, and serving as a proton shuttle to facilitate protodemetalation (Scheme 1c).

## Results and Discussion

### Exploration of the Viability of *anti*-Acetoxylation

Based on previous studies for the *anti*-acetoxylation of alkynes,^20^ we initially tested model substrate **1a** under a different survey of metal catalysts using AcOH both as a solvent and reagent (Table 1). To our delight, we observed that Pd(OAc)_2_ delivered the corresponding acetoxylated product *Z*-**2a** in 86% isolated yield, with complete β-regio- and *anti*-stereoselectivities after 1 h at 80 °C (entry 1). Other precious metals with a high affinity toward alkyne activation such as Pt(CH_3_CN)_2_Cl_2_ and Au(PPh_3_)Cl/AgOTf delivered the product *Z*-**2a** along with considerable amounts of the byproduct **3a** (entries 2 and 3, respectively). When AgSbF_6_ was used as a catalyst, complete decomposition was observed (entry 4), while other metal salts such as Zn(ClO_4_)_2_ did not promote any reaction (entry 5). Having identified Pd(OAc)_2_ as a suitable catalyst, we explored lower loadings but the reduced yield of *Z*-**2a** was observed when using 3 mol % catalyst (41% yield, entry 6). A slight reduction in temperature to 60 °C afforded the product *Z*-**2a** in synthetically useful 76% yield, but longer reaction times were necessary to achieve full conversion (5 h, entry 7). Other palladium(II) sources lacking any acetate ligand such as PdBr_2_, Pd(acac)_2_, Pd(TFA)_2_, and Pd(CH_3_CN)_2_Cl_2_ provided the product in very low yield or completely suppressed the reaction, resulting in decomposition of the starting material (entries 8–11), which points toward an important role of the acetate ligand. In addition, no product was detected if palladium was omitted in the reaction (entry 12).

**Table 1:**
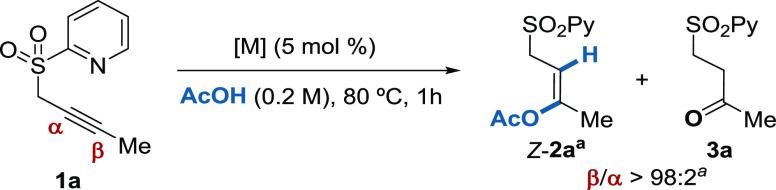
Optimization Studies for the Acetoxylation of Substrate **1a**

entry	catalyst	*Z*-**2a**/**3a**^a^	yield (%)^b^
1	Pd(OAc)_2_	>98:2	86
2	Pt(CH_3_CN)_2_Cl_2_	44:56	72
3	AuCl(PPh_3_)/AgOTf	62:38	66
4	AgSbF_6_	decomp	
5	Zn(ClO_4_)_2_·6H_2_O	nr	
6^c^	Pd(OAc)_2_	>98:2	41
7^d^	Pd(OAc)_2_	>98:2	76
8	PdBr_2_	nr	
9	Pd(acac)_2_	>98:2	10
10	Pd(TFA)_2_	nr	
11	PdCl_2_(CH_3_CN)_2_	decomp	
12	none	nr	
13	Pd/Au^e^	94:6	79
14	Pd(OAc)_2_,H_2_O^f^	86:14	81
15	Pd/Au^e^,H_2_O^f^	52:48	85

a Determined by ^1^H NMR spectroscopy from the crude mixture.

b Determined by ^1^H NMR using 1,3,5-trimethoxybenzene as an internal standard.

c 3 mol % Pd(OAc)_2_ was used.

d Reaction performed at 60 °C during 5 h for full completion.

e A combination of Pd(OAc)_2_ (5 mol %) and AuCl(PPh_3_)/AgOTf (5 mol %) was used as a catalyst.

f Extra water (10% v/v) was added to the reaction mixture. nr: no reaction (starting material recovered).

Formation of ketone **3a** led us to question whether it arises from enol acetate deprotection or from a competing attack of water onto the corresponding π-complex intermediate. To gain an insight into this aspect, we performed the reaction of **1a** catalyzed by AuCl(PPh_3_)/AgOTf but in the presence of Pd(OAc)_2_ as a cocatalyst (5 mol %). Interestingly, this change led to a dramatic increase in selectivity toward the acetoxylation product **2a** from **2a**/**3a** = 62:38 to 94:6 (Table 1, entry 13). This result provides compelling evidence that ketone **3a** arises from the attack of water present in AcOH onto the π-complex intermediate, followed by protodemetalation and also suggests that the acetate ligand on Pd(OAc)_2_ may play an important role in controlling chemoselectivity. Even when extra 10% v/v water was added to the reaction mixture under Pd(OAc)_2_ catalysis, the acetoxylation product was formed with good selectivity (**2a**/**3a** = 86:14, entry 14). The addition of extra water (10% v/v) in the presence of a combination of gold and palladium catalysis under otherwise identical conditions revealed significant loss of selectivity, likely due to competing Au-catalyzed hydration (**2a**/**3a** = 52:48, entry 15).

**Scheme 2:**
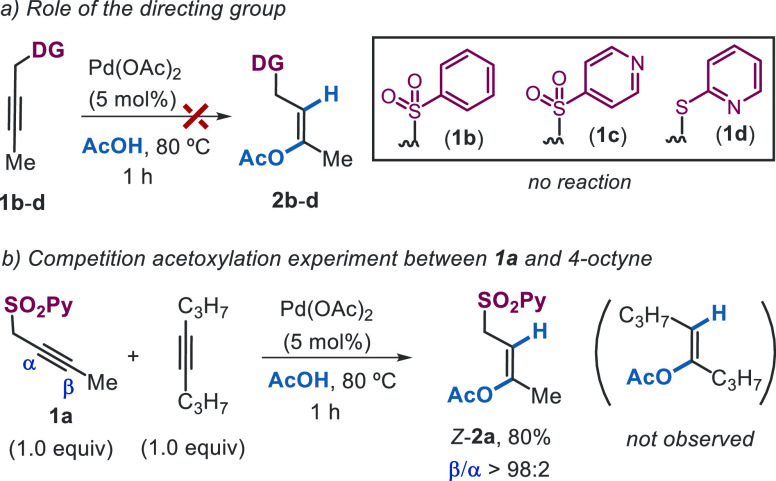
Importance of the Directing Group and Competition Experiment

Then, to test the role of the SO_2_Py directing group in controlling reactivity and selectivity, substrates bearing at the propargylic position related groups with different electronic and coordinating properties were screened under the optimized conditions (Scheme 2a). The SO_2_Ph analogue **1b** proved to be unreactive, being fully recovered after 1 h, and an identical result was found for alkyne **1c**, an electronically similar isomer of **1a** carrying the 4-pyridyl sulfonyl group. These results highlight the essential role of the coordinating 2-pyridyl unit for ensuring not only regiocontrol but also reactivity. Interestingly, the lack of reactivity showed by the 2-pyridyl sulfonyl alkyne **1d** emphasizes the cooperative directing role of both the sulfonyl-tethering group and the 2-pyridyl moiety, presumably by weakening its metal-coordinating ability to facilitate catalytic turnover.

The strong reliance of alkyne acetoxylation on the directing effect of the SO_2_Py moiety could be exploited to achieve chemoselective functionalization of alkynes. This hypothesis was validated in an intermolecular competition reaction between **1a** and 4-octyne under the standard reaction conditions, which revealed that only **1a** underwent smooth acetoxylation, providing **2a** in 80% yield and complete β-regioselectivity (Scheme 2b), with no acetoxylation of 4-octyne being detected. This result points toward an essential role of the SO_2_Py group in promoting acetoxylation.

With suitable conditions for the acetoxylation of substrate **1a** in our hands, we next questioned the need for using AcOH as a solvent by examining the effect of solvent in the reaction of **1a** in the presence of 2 equiv of AcOH (Table 2). In comparison with the use of AcOH (entry 1), a dramatic decrease in reactivity was observed under nonpolar solvents such as toluene (7%, entry 2). Slightly better reactivity was observed when switching to more polar solvents such as 4-CF_3_-C_6_H_5_ or 1,2-dichloroethane, albeit the yield was not synthetically useful (21%, entries 3 and 4). Interestingly, highly polar solvents such as dimethylformamide (DMF) or ^i^PrOH that could act as a stabilizing ligand occupying open coordination sites on the metal proved to be detrimental to reactivity, completely inhibiting the product formation (entries 5 and 6). In this case, we hypothesized that the catalytically active Pd(OAc)_2_ species could interact with the solvent through hydrogen bonding, thus precluding the activation of the incoming carboxylic acid during the nucleometalation of the alkyne.^21^ The same negative result was observed when 1,4-dioxane was used as a solvent (entry 7). However, when 1,1,1,3,3,3-hexafluoro-2-propanol (HFIP) was employed, the product *Z*-**2a** was obtained in 46% yield (entry 8).^22^ Using these conditions, the isolated yield of *Z*-**2a** could be further improved to 61% upon increasing the reaction time to 18 h (entry 9). These results strongly suggest that a drastic depletion of the reactivity was observed when using a solvent different from AcOH, with the exception of HFIP, thus proving the challenging nature of this transformation.

**Table 2:**
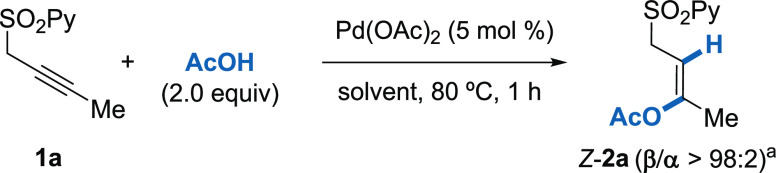
Solvent Effect in the Acetoxylation of **1a**

entry	solvent	yield (%)^a^
1	AcOH	86
2	toluene	7
3	4-CF_3_-C_6_H_5_	21
4	DCE	21
5	DMF	
6	^*i*^PrOH	
7	1,4-dioxane	
8	HFIP	46
9^b^	HFIP	61

a Determined by ^1^H NMR spectroscopy in the reaction crude.

b Reaction run for 18 h.

**Scheme 3:**
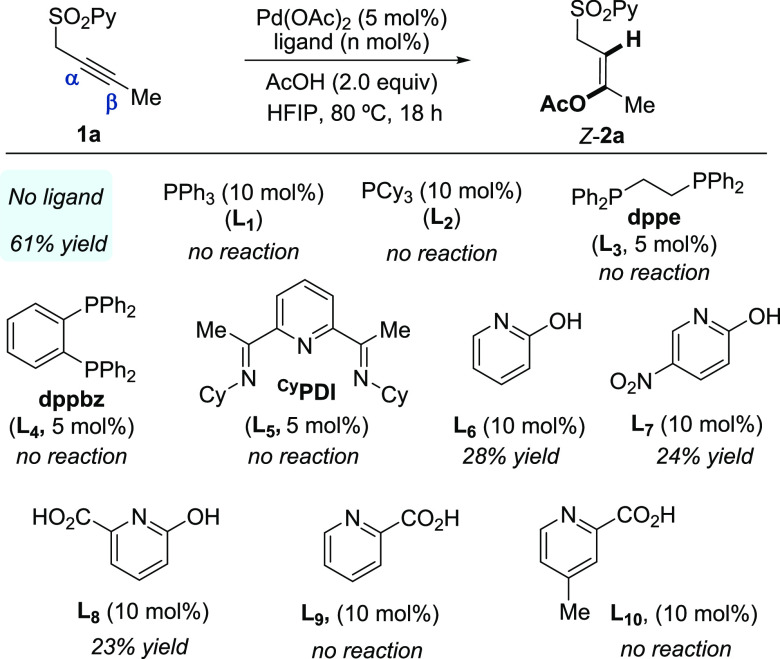
Ligand Effect in the Acetoxylation Reaction

The effect of ligands in the acetoxylation of **1a** was also investigated (Scheme 3). In this study, we decided to use HFIP as a solvent (conditions of entry 9 in Table 2) to favor the complexation of the metal to the added ligand and minimize possible interference caused by a large excess of AcOH. The examination of a variety of mono- (PPh_3_ and PCy_3_) and bidentate (dppe and dppbz) phosphines commonly used in organic synthesis and pincer-type nitrogen ligands such as bis(imino)pyridines (^Cy^PDI) completely inhibited the reaction. Interestingly, X-type pyridine-based ligands such as 2-hydroxypyridines/2-pyridones delivered the product *Z*-**2a** but only in low yield (**L**_**6**_–**L**_**8**_, 23–28%). This type of ligand is able to assist the dissociation of trimeric palladium acetate [Pd_3_(OAc)_6_].^23^ In contrast, pyridine-2-carboxylic acid derivatives were totally ineffective ligands (**L**_**9**_–**L**_**10**_). We speculate that the external ligand might hinder the simultaneous coordination of palladium to the substrate (in a bidentate fashion) and the acetate ligand, which could be key to enabling catalysis. Consequently, the ligandless conditions found initially in Table 1 (entry 1) were selected as optimal for the acetoxylation reaction.

### Structural Scope for the *anti*-Acetoxylation

**Scheme 4:**
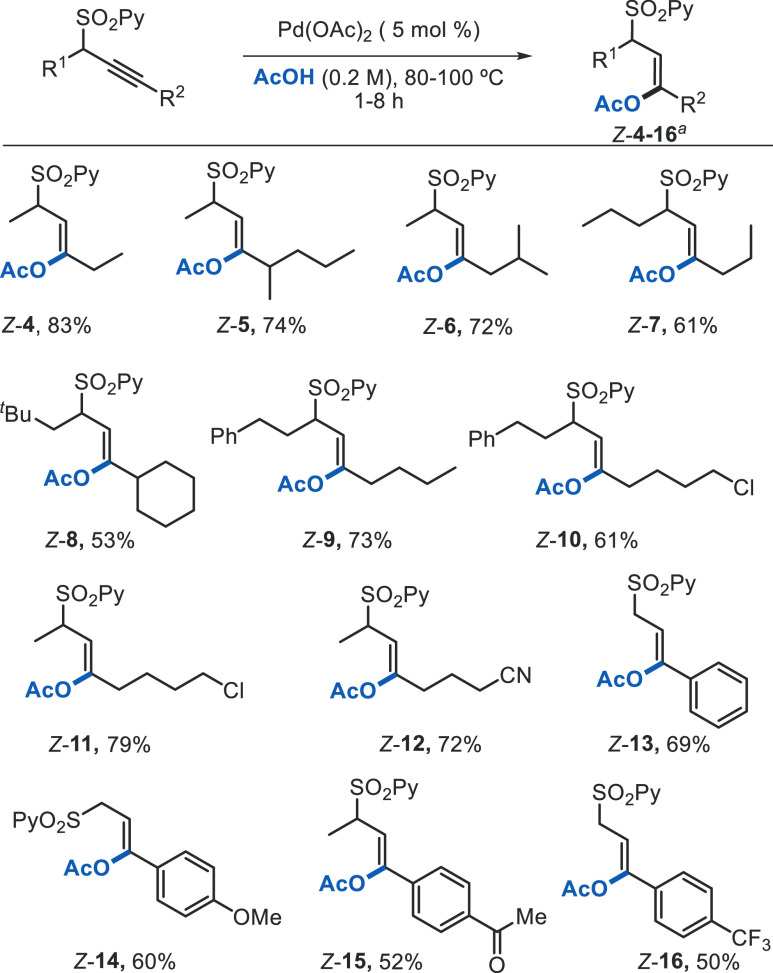
Representative Substrate Scope for the Acetoxylation of Unsymmetrical Internal Alkynes Employing the SO_2_Py Group as a Regiocontroller Regio- and stereochemistry of all products were determined in the reaction crude by ^1^H NMR spectroscopy. Complete levels of selectivity (β/α > 98:2; *Z*/*E* > 98:2) were observed in all cases studied. Reaction yields refer to products isolated after purification by flash column chromatograpy.

Having established an efficient catalytic system for the β-acetoxylation of substrate **1a**, we set out to investigate the versatility of the reaction toward more challenging substrates (Scheme 4). Gratifyingly, this method is compatible with the presence of an alkyl substituent at the propargylic position without erosion of the reaction yield (83%, *Z*-**4**). The use of substrates with larger alkynyl substituents was also well-tolerated, maintaining the exceptional levels of regio- and stereoselectivities (products *Z*-**5** and *Z*-**6**, 74 and 72% yield, respectively). Larger alkyl groups at the propargylic position, such as *^n^*Bu, phenethyl, or even ^*t*^Bu, were also amenable to the reaction without affecting the regio- or stereoselectivity (*Z*-**7**-**9**, 53–73%). Importantly, potentially sensitive functional groups such as alkyl halide or nitrile were also accommodated with no observable impact in yield or selectivity (products *Z*-**10**–**12**, 61–79% yield). Interestingly, the SO_2_Py directing group is capable to override the inherent electronic bias due to conjugation imposed by the aromatic substituent in internal aryl-substituted alkynes, since this class of alkynes typically undergoes insertion with opposite regioselectivity.^9^ As illustrated for products *Z*-**13**–**16**, the excellent β-regioselectivity was maintained for some representative aryl alkynes regardless of the electronic character of the aromatic ring. In all cases studied, the corresponding acetoxylation products were isolated as single regioisomers in synthetically useful yields, even for substrates containing strong electron-withdrawing groups (*Z*-**15** and ***Z*****-16**, 52 and 50%, respectively). Unfortunately, terminal alkynes were incompatible, largely producing catalyst deactivation. On the other hand, primary hydroxyl- and carboxylic acid-containing alkynes proved not to be suitable in this reaction, resulting in deactivation of the catalyst or decomposition of the starting material (not shown, see the Supporting Information, SI for further details).

### Kinetic Analysis

**Figure 1:**
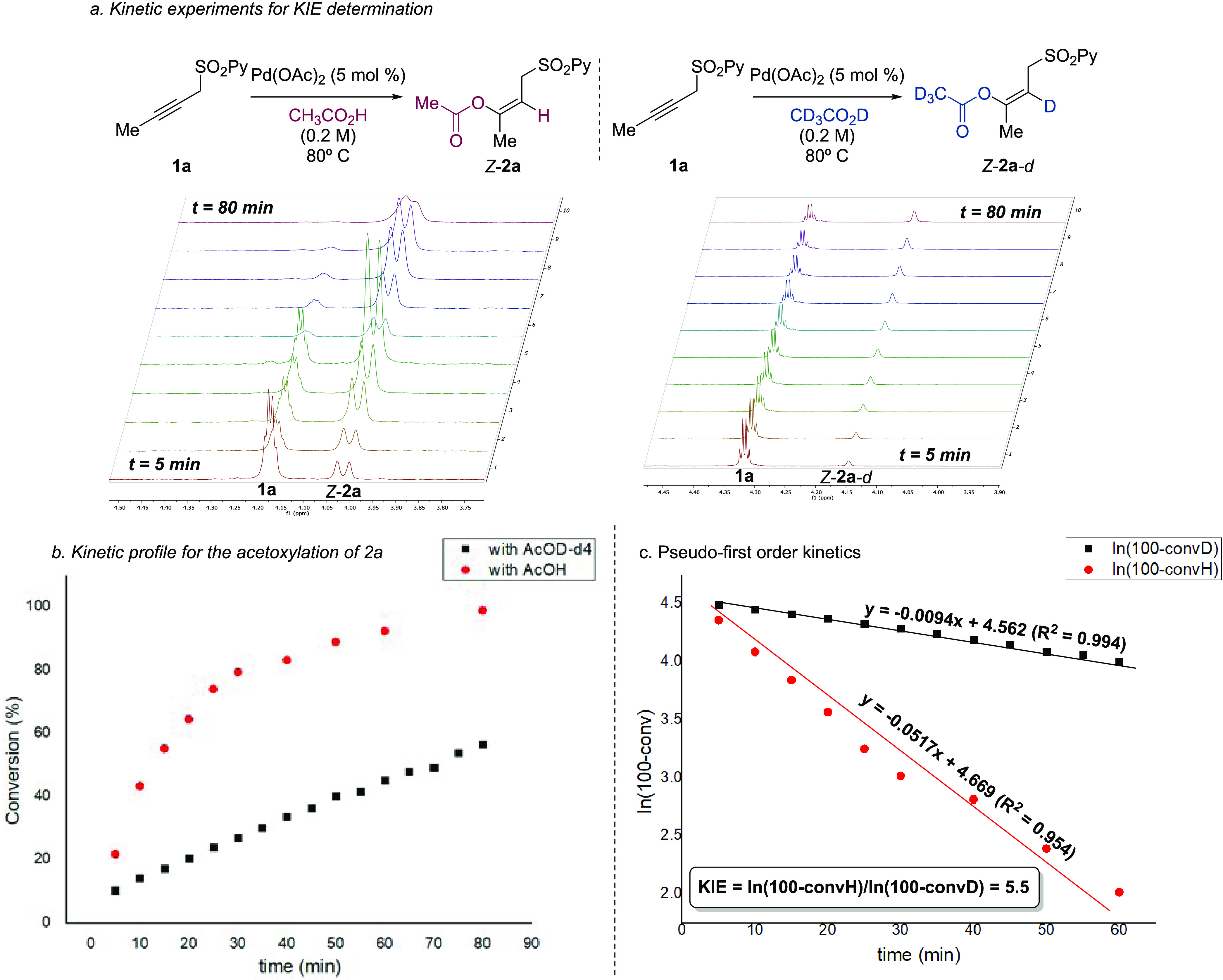
(Top) Time course of the acetoxylation of **1a** by ^1^H NMR spectroscopy in the presence of acetic acid (left) or acetic acid-*d*_4_ (right). (Bottom) Determination of the KIE for the acetoxylation of **1a**.

To further understand the role of acetic acid in the reaction, we performed a kinetic analysis to determine the presence of a primary kinetic isotope effect (KIE) by monitoring the acetoxylation of substrate **1a** in AcOH and AcOD-*d*_4_ by independent experiments (Figure 1). From this study, we observed a clear effect in the reaction rate for the acetoxylation of propargyl sulfone **1a**, in which a slower formation of the acetoxylated product took place when acetic acid-*d*_4_ was employed both as a solvent and reagent (Figure 1a,b). In addition, the plot of the ln(100-conversion) vs time for the nondeuterated and deuterated kinetic profiles led to a linear fit, resulting in a pseudo-first-order kinetics from which a primary KIE of 5.5 was obtained (Figure 1c, see the SI for details). These results suggest that either the addition of the acid or the protodepalladation step could be the rate-determining step of the reaction.

### Computational Analysis

**Figure 2:**
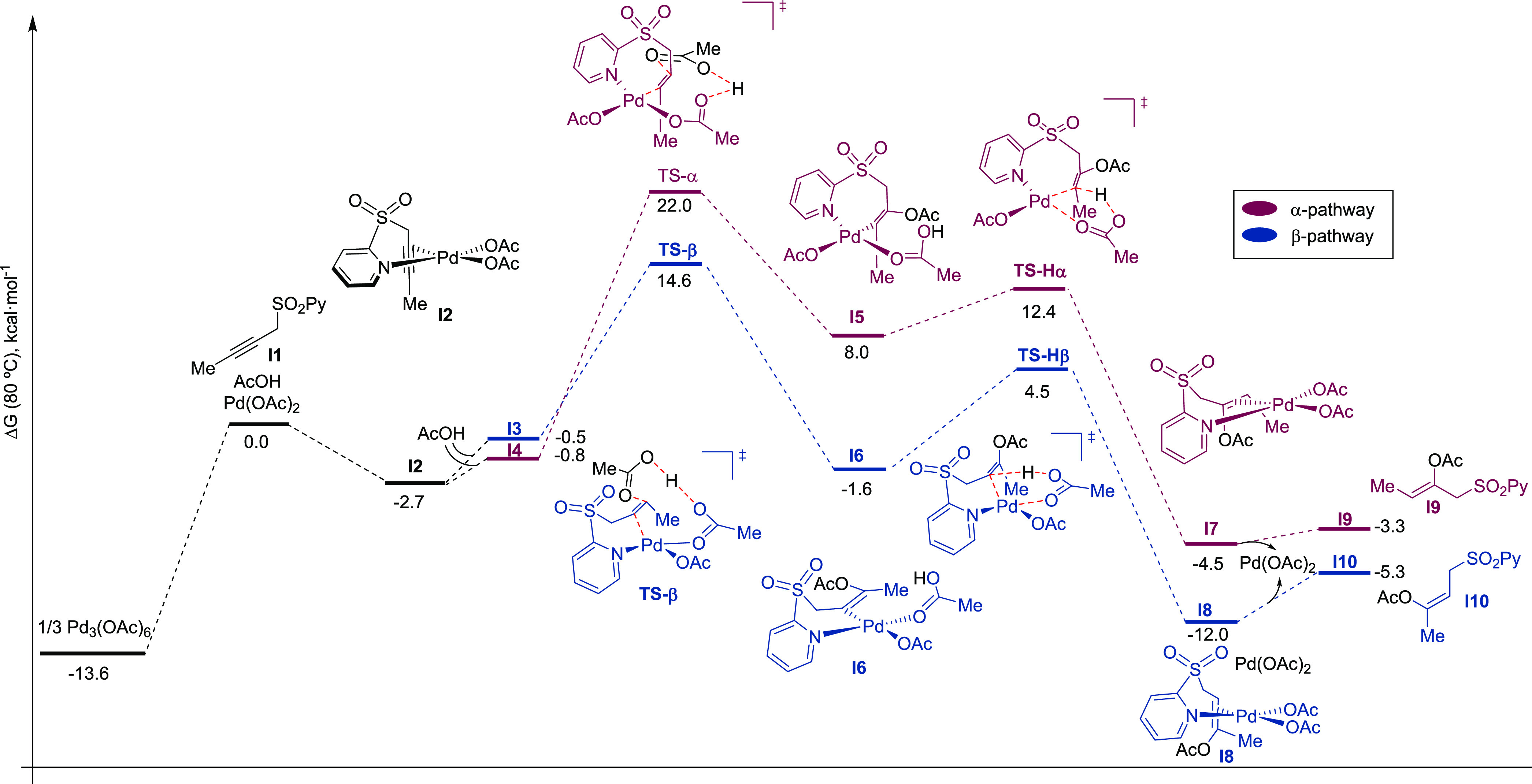
Reaction profiles for the intermolecular α- (magenta) and β-acetoxylation (blue). Gibbs free energies in kcal/mol at 353.15 K are given relative to the sum of all reagents (**1a** + AcOH + Pd(OAc)_2_) at an infinite distance.

To gain insight into the reaction mechanism, we conducted a computational analysis considering several pathways. These calculations were carried out using the M06-L functional. For gas-phase geometry optimizations and frequency calculations, the def2-SVP basis set with effective core potential (ECP) was used for Pd, and the cc-pVDZ basis set was used for all other atoms (C, H, N, O, S). Single-point energy calculations with an SMD continuum solvation model were performed using the def2-TZVP basis set with ECP for Pd, and the cc-pVTZ basis set for all other atoms (see the SI for further details). To analyze the intrinsic reactivity of the propargyl sulfone **1a** (**I1** in Figure 2) either free in solution or bound to Pd(OAc)_2_, we first calculated the condensed Fukui functions^24^ at the C_α_ and C_β_ positions ( Figure 2). These results showed that, albeit the triple bond is naturally polarized, it becomes more electrophilic upon Pd coordination (see the SI); in both cases, the C_β_ position is more electrophilic. However, the rather low difference between the two positions (+0.028 for C_α_ and +0.051 for C_β_) suggests that the selectivity toward the β-adduct cannot be exclusively accounted for by electrophilicity. Thus, we have calculated the complete reaction profiles for the α- and β-acetoxylation (Figure 2). We found that the reaction follows a stepwise mechanism in both cases. Initially, we observed that the complexation of propargyl sulfone **1a** with Pd(OAc)_2_ is thermodynamically feasible to form the corresponding chelate species **I2**. From this intermediate, we further explored the addition of AcOH to both positions of the C–C triple bond. The first step corresponds to the AcOH addition to the triple bond and the concomitant proton migration from the incoming AcOH molecule to one of the acetate ligands bound to Pd. From both alkenyl-palladium(II) intermediates (**I5** and **I6**), intramolecular protodepalladation can take place to form the final alkenes (**I9** and **I10**) and regenerate Pd(OAc)_2_ upon de-coordination from the product. The selectivity is determined at the first step, given that the formation of the 6-membered palladacycle (β-addition) is kinetically (ΔΔ*G*^≠^ = (**TS-β**-**I3**)-(**TS-α**-**I4**) = −7.7 kcal/mol) and thermodynamically (exergonic, ΔΔ*G*_reac_ = (**I6**-**I3**)-(**I5**-**I4**) = −9.9 kcal/mol) more favorable. In contrast, the protodepalladation step proceeds very similarly for both pathways (ΔΔ*G*^≠^ = (**TS-Hβ**-**I6**)-(**TS-Hα**-**I5**) = +1.7 and ΔΔ*G*_reac_ = (**I8**-**I6**)-(**I7**-**I5**) = +2.1 kcal/mol) in an intramolecular fashion since one AcOH is coordinated to the palladium center after the addition of the carboxylic acid. Alternative reaction pathways (coordination of an additional AcOH molecule and intramolecular *syn* insertion of the alkyne into the Pd—OAc bond) are discussed in the SI, none of them were found to be more favorable than the β-acetoxylation presented here. These conclusions help to understand the key role of the acetate ligand since it assists in the formation of the C–O bond during the acetoxypalladation event through proton migration. This reactivity closely resembles the more typical concerted metalation–deprotonation (CMD) pathway observed in some C–H activation processes.^2b^ Therefore, the acetate ligand behaves as a multifunctional ligand during the addition of the carboxylic acid and further protodemetalation. This observation is in accordance with the lack of reactivity observed when different strong coordinating ligands are present since they are not expected to be active in the proton migration event. In contrast, when 2-hydroxypyridines were employed, the reaction delivered the acetoxylated product in low yields, pointing out to the potential role as acid activators of these *N*-coordinating species during the proton migration.^25^ Additionally, this activation mode is observed in the transition state of the rate-determining step (the acetoxypalladation), thus explaining the KIE obtained when compared with the reaction kinetics in AcOH and AcOD-*d*_4_.

**Figure 3:**
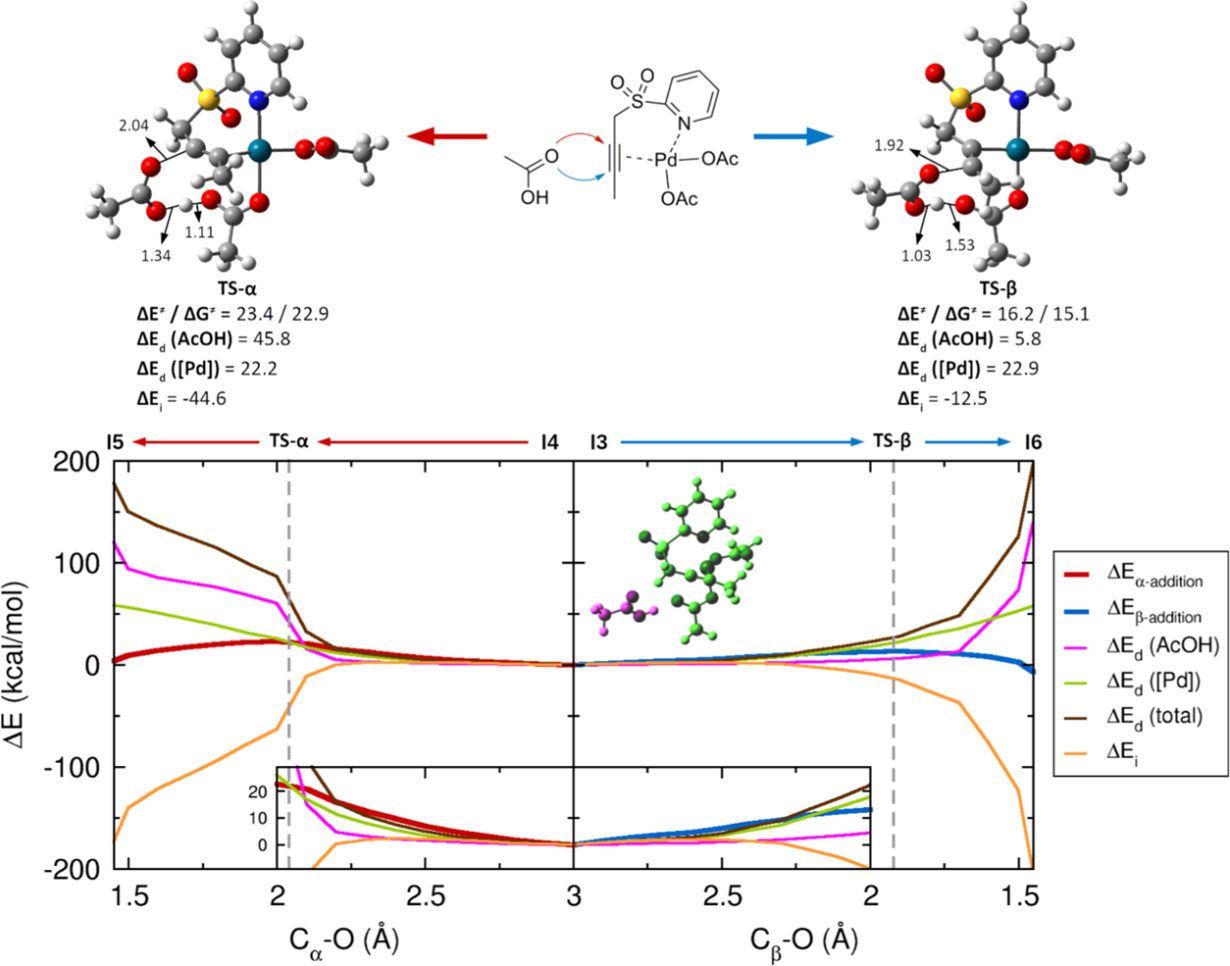
(Top) Activation energies and Gibbs free energies and distortion–interaction energy contributions at the TSs. Energies in kcal/mol. (Bottom) Distortion/interaction diagrams for the intermolecular α- (left) and β-acetoxylation (right) projected onto the C–O distance. Vertical dashed lines mark the position of TS-α or TS-β. [Pd] accounts for the **1a**-Pd(OAc)_2_ complex. A zoom of the region comprised between a C–O distance value of 2 and 3 Å is provided in the inset. Distances are given in Å, energies in kcal/mol.

To further understand the origin of the differences in the activation energies of the first step of the reaction, we analyzed the energy profiles by means of the distortion/interaction model.^26^ In short, this model proposes that the reaction energy at any point of the reaction coordinate can be divided into two components, one associated with the reagents’ geometry deformations (the distortion energy, Δ*E*_d_), and the stabilization produced when the distorted reagents interact (the interaction energy, Δ*E*_i_). Figure 3 shows the distortion/interaction diagrams for the α- (left) and β-acetoxylation (right). To carry out this analysis, we divided the reaction complex into two subunits, the incoming AcOH molecule and the **1a**-Pd(OAc)_2_ complex. While the Δ*E*_d_ ([Pd]) curves (the distortion energy associated with the **1a**-Pd(OAc)_2_ complex) are similar, the main difference between both reaction pathways lies in Δ*E*_d_ (AcOH), which starts to increase earlier in the α-addition than in the β-addition. In fact, at the position of **TS-α** (recall Figure 3), Δ*E*_d_ (AcOH) is not only larger than Δ*E*_d_ ([Pd]) but also slightly larger (in absolute values) than Δ*E*_i_, making it the predominant term for the activation energy. In contrast, for **TS-β**, the contribution of Δ*E*_d_ (AcOH) is minimal. This critical difference can be related to the moment at which proton migration takes place. For the α-addition, the lower electrophilicity of the C_α_ position requires that O–H dissociation occurs in an early stage to increase AcOH nucleophilicity and to facilitate C_α_–O bond formation, but the cost of the O–H bond cleavage is larger than the gain in reactivity. In contrast, as the C_β_ position is more reactive, O–H dissociation can develop after C_β_–O bond formation and would occur after the **TS-β**, so the energy required to O–H dissociation is clearly compensated by Δ*E*_i_ along the whole pathway.

**Figure 4:**
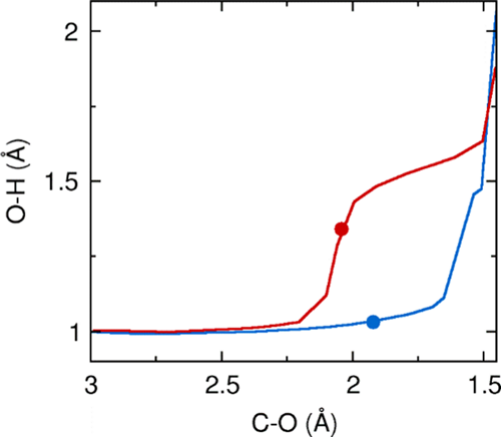
Evolution of the O–H and C–O distances along the α- (red) and β-acetoxylation (blue) reaction pathways. Full dots indicate the position of TS-α and TS-β.

The above-mentioned factor determines that the proton migration plays a critical role in favoring the β-acetoxylation over the α-, as for the α-addition the O–H cleavage is required in an early stage and thus increases Δ*E*_d_ (AcOH) to a point that it cannot be compensated by Δ*E*_i_, while for the β-addition, it occurs at the end of the reaction. This can be proved easily by plotting the O–H distance against the C–O (Figure 4); in the α-addition (red pathway), the O–H dissociation occurs before C–O formation and starts before the TS, while the opposite behavior is observed for the β-addition.

**Figure 5:**
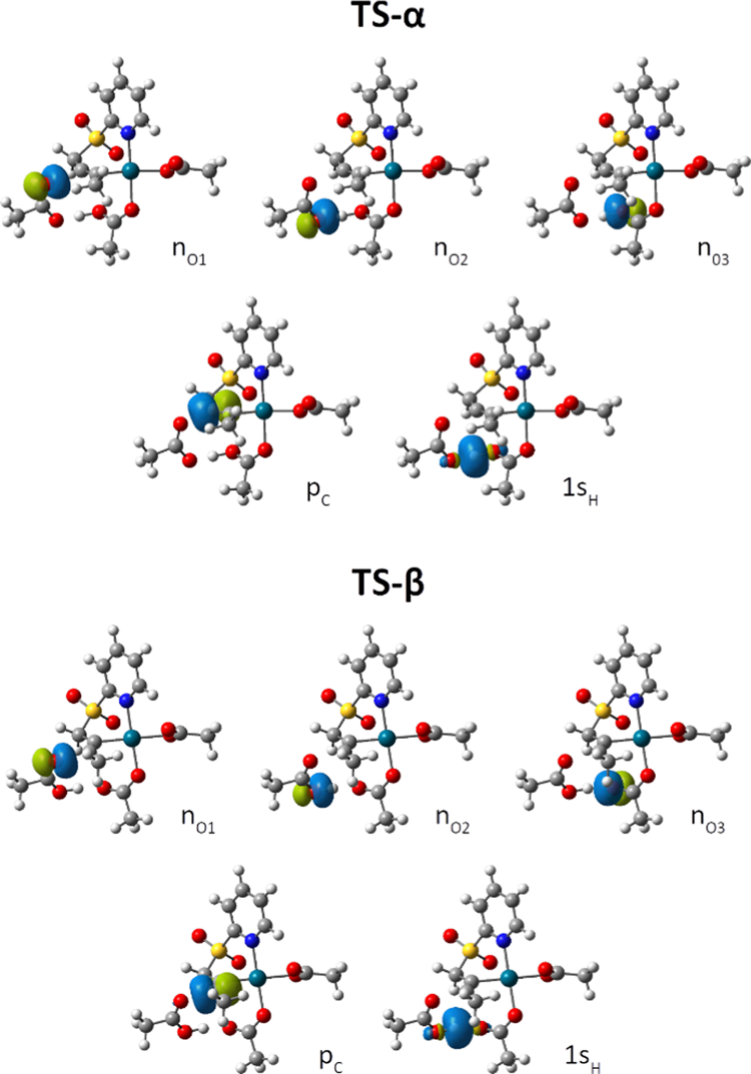
NBO orbitals of TS-α (top) and TS-β (bottom) considered in this study.

To further corroborate our hypothesis on the differential asynchronicity in the bond formation at **TS-α** and **TS-β**, we have resorted to a NBO analysis to evaluate/quantify the interaction between the two approximating moieties (Figure 5, top and bottom, respectively). For both transition states, the C_α_–C_β_ is no longer a triple bond, given that the C–Pd bond is already formed, and a virtual p orbital can be located over the other carbon center. However, the formation of the new C–O bond is in a more advanced state in **TS-β**, as reflected by the greater second-order interaction energy between the oxygen lone pair and the carbon p orbital of the **1a**-Pd(OAc)_2_ complex (Table 3, 107 kcal/mol in **TS-β** vs 68 kcal/mol in **TS-α**). In a similar vein, the original O–H bond of the incoming acetic acid is broken, while the new one (with the acetate ligand) is yet to be formed. As was discussed before, the proton transfer takes place earlier in the α-addition than in the β-addition. Consequently, the second-order interaction energy between the empty 1s orbital of the transferred proton and the oxygen lone pair of the acetate ligand is greater in **TS-α** (276 kcal/mol) than that in **TS-β** (49 kcal/mol). On the other hand, the second-order interaction energy between the 1s_H_ and the oxygen lone pair of acetic acid is greater in **TS-β** (354 kcal/mol) than that in **TS-α** (129 kcal/mol).

**Table 3:** Second-Order Interaction Energies (in kcal/mol) between Selected NBO Orbital Pairs for TS-α and TS-β

orbital pair	TS-α	TS-β
n_O1_ → p_C_	68	107
n_O2_ → 1s_H_	129	354
n_O3_ → 1s_H_	276	49

## Conclusions

In summary, this study of the acetoxylation reaction of unsymmetrical dialkyl alkynes with AcOH shows that the cooperative effects of the palladium metal, the SO_2_Py directing group at the propargylic position, and the acetate ligand play essential roles in reaction efficiency and selectivity control. The corresponding alkenyl acetates are obtained with good yields and excellent level of regioselectivity (acetate delivered distal to the directing group) and *anti*-addition stereoselectivity. Experimental and computational mechanistic analyses suggest that the acetate ligand plays multiple important roles. It brings the AcOH near the metal–substrate complex through hydrogen bonding, enhances its nucleophilicity, and directs the attack to the alkyne in a regioselective fashion. Additionally, once protonated, it also facilitates protodematallation acting as a proton shuttle to protonate the alkenyl-Pd intermediate intramolecularly. These studies revealed that the *anti*-acetoxypalladation of the alkyne is both a rate-determining and regioselectivity-determining step of the catalytic cycle. The two regiochemical outcomes have been analyzed in detail using the distortion/interaction model, showing that the proton migration plays a critical role in favoring the β-acetoxylation over the α-attack. These observations provide a blueprint for the rational design of more efficient methods to attain regiocontrol in the functionalization of internal alkynes through the addition of polar X—H bonds. Further studies regarding the synthetic potential of the resulting alkenyl acetates and directing group transformations are under study in our laboratories and will be reported in due course.

## Supporting Information Available

Experimental procedures; ^1^H and ^13^C NMR spectra for all new compounds; and DFT calculations (PDF)

## References

[ref1] Crabtree R. H. Multifunctional ligands in transition metal catalysis. New J. Chem. 2011, 35, 18–23. 10.1039/C0NJ00776E.

[ref2] aMusaev D. G.; Liebeskind L. S. On the Mechanism of Palladium(0) Catalyzed, Copper(I) Carboxylate Mediated Thioorganic-Boronic Acid Desulfitative Coupling. A Noninnocent Role for the Carboxylate Ligand. Organometallics 2009, 28, 4639–4642. 10.1021/om900602b. 20161122 PMC2742425

[ref3] aGarcía-Cuadrado D.; de Mendoza P.; Braga A. A. C.; Maseras F.; Echavarren A. M. Proton-Abstraction Mechanism in the Palladium-Catalyzed Intramolecular Arylation: Substituent Effects. J. Am. Chem. Soc. 2007, 129, 6880–6886. 10.1021/ja071034a. 17461585

[ref4] aGuihaumé J.; Halbert S.; Eisenstein O.; Perutz R. N. Hydrofluoroarylation of Alkynes with Ni Catalysts. C–H Activation via Ligand-to-Ligand Hydrogen Transfer, an Alternative to Oxidative Addition. Organometallics 2012, 31, 1300–1314. 10.1021/om2005673.

[ref5] aJohnson D. G.; Lynam J. M.; Slattery J. M.; Welby C. E. Insights into the Intramolecular Acetate-Mediated Formation of Ruthenium Vinylidene Complexes: a Ligand-Assisted Proton Shuttle (LAPS) Mechanism. Dalton Trans. 2010, 39, 10432–10441. 10.1039/c0dt00431f. 20938549

[ref6] Wang Y.; Wang Z.; Li Y.; Wu G.; Cao Z.; Zhang L. A General Ligand Design for Gold Catalysis Allowing Ligand-Directed anti-Nucleophilic Attack of Alkynes. Nat. Commun. 2014, 5, 3470 10.1038/ncomms4470. 24704803 PMC4119785

[ref7] Cheng X.; Zhang L. Designed Bifunctional Ligands in Cooperative Homogeneous Gold Catalysis. CCS Chem. 2021, 3, 1989–2002. 10.31635/ccschem.020.202000454.

[ref8] aLeeb N. M.; Drover M. W.; Love J. A.; Schafer L. L.; Slattery J. M. Phosphoramidate-Assisted Alkyne Activation: Probing the Mechanism of Proton Shuttling in a N,O-Chelated Cp*Ir(III) Complex. Organometallics 2018, 37, 4630–4638. 10.1021/acs.organomet.8b00656.

[ref9] aZeng X. Recent Advances in Catalytic Sequential Reactions Involving Hydroelement Addition to Carbon-Carbon Multiple Bonds. Chem. Rev. 2013, 113, 6864–6900. 10.1021/cr400082n. 23659649

[ref10] aCheng Z.; Guo J.; Lu Z. Recent Advances in Metal-Catalysed Asymmetric Sequential Double Hydrofunctionalization of Alkynes. Chem. Commun. 2020, 56, 2229–2239. 10.1039/D0CC00068J. 32040117

[ref11] Alonso F.; Beletskaya I. P.; Yus M. Transition-Metal-Catalyzed Addition of Heteroatom–Hydrogen Bonds to Alkynes. Chem. Rev. 2004, 104, 3079–3160. 10.1021/cr0201068. 15186189

[ref12] aLin C.; Shen L. Recent Progress in Transition Metal-Catalyzed Regioselective Functionalization of Unactivated Alkenes/Alkynes Assisted by Bidentate Directing Groups. ChemCatChem 2018, 11, 961–968. 10.1002/cctc.201801625.

[ref13] aMoure A. L.; Arrayás R. G.; Cárdenas D. J.; Alonso I.; Carretero J. C. Regiocontrolled Cu^I^-Catalyzed Borylation of Propargylic-Functionalyzed Internal Alkynes. J. Am. Chem. Soc. 2012, 134, 7219–7222. 10.1021/ja300627s. 22500739

[ref14] aRuppin C.; Dixneuf P. H. Synthesis of Enol Esters from Terminal Alkynes Catalyzed by Ruthenium Complexes. Tetrahedron Lett. 1986, 27, 6323–6324. 10.1016/S0040-4039(00)87798-9.

[ref15] Lumbroso A.; Vautravers N. R.; Breit B. Rhodium-Catalyzed Selective anti-Markovnikov Addition of Carboxylic Acids to Alkynes. Org. Lett. 2010, 12, 5498–5501. 10.1021/ol102365e. 21049947

[ref16] aLu X.; Zhu G.; Ma S. A Novel Regio- and Stereo-Specific Hydroacetoxylation Reaction of 2-Alkynoic Acid Derivatives. Tetrahedron Lett. 1992, 33, 7205–7206. 10.1016/S0040-4039(00)60873-0.

[ref17] aRoembke P.; Schmidbaur H.; Cronje S.; Raubenheimer H. Application of (Phosphine)Gold(I) Carboxylates, Sulfonates and Related Compounds as Highly Efficient Catalysts for the Hydration of Alkynes. J. Mol. Catal. A: Chem. 2004, 212, 35–42. 10.1016/j.molcata.2003.11.011.

[ref18] aMukaiyama T. The Directed Aldol Reaction. Org. React. 1982, 28, 203–331.

[ref19] aDupuy S.; Gasperini D.; Nolan S. P. Highly Efficient Gold (I)-Catalyzed Regio- and Stereoselective Hydrocarboxylation of Internal Alkynes. ACS Catal. 2015, 5, 6918–6921. 10.1021/acscatal.5b02090. 29291135 PMC5745071

[ref20] aZhang Q.; Lu X. Highly Enantioselective Palladium(II)-Catalyzed Cyclization of (Z)-4′-Acetoxy-2′-butenyl 2-Alkynoates: An Efficient Synthesis of Optically Active γ-Butyrolactones. J. Am. Chem. Soc. 2000, 122, 7604–7605. 10.1021/ja001379s.

[ref21] The acetoxylation of substrate **1a** in AcOH under different amounts of ^*i*^PrOH demonstrated an inverse relationship between conversion toward the acetoxylation product and the amount of ^*i*^PrOH added, suggesting that a competing interaction between the acetate ligand and the ^*i*^PrOH via hydrogen-bonding could disrupt the activation of the incoming carboxylic acid: 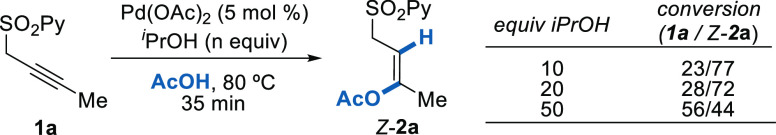

[ref22] Bai Z.; Bai Z.; Song F.; Wang H.; Chen G.; He G. Palladium-Catalyzed Amide-Directed Hydrocarbofunctionalization of 3-Alkenamides with Alkynes. ACS Catal. 2020, 10, 933–940. 10.1021/acscatal.9b04285.

[ref23] Mandal N.; Datta A. Harnessing the Efficacy of 2-Pyridone Ligands for Pd-Catalyzed (β/γ)-C(sp^3^)–H Activations. J. Org. Chem. 2020, 85, 13228–13238. 10.1021/acs.joc.0c02210. 32975420

[ref24] Ayers P. W.; Morrison R. C.; Roy R. K. Variational Principles for Describing Chemical Reactions: Condensed Reactivity Indices. J. Chem. Phys. 2002, 116, 8731–8744. 10.1063/1.1467338.

[ref25] aWang P.; Farmer M. E.; Huo X.; Jain P.; Shen P.-X.; Ishoey M.; Bradner J. E.; Wisniewski S. R.; Eastgate M. D.; Yu J.-Q. Ligand-Promoted Meta-C—H Arylation of Anilines, Phenols, and Heterocycles. J. Am. Chem. Soc. 2016, 138, 9269–9276. 10.1021/jacs.6b04966. 27384126 PMC5512281

[ref26] Bickelhaupt F. M.; Houk K. N. Analyzing Reaction Rates with the Distortion/Interaction-Activation Strain Model. Angew. Chem., Int. Ed. 2017, 56, 10070–10086. 10.1002/anie.201701486. PMC5601271 28447369

