# Supplementary material for: Interplay between the Directing Group and Multifunctional Acetate Ligand in Pd-Catalyzed anti-Acetoxylation of Unsymmetrical Dialkyl-Substituted Alkynes
Source: ACS Catal. 2022 May 19;12(11):6596–605. doi: 10.1021/acscatal.2c00710 (PMC9173690; doi:10.1021/acscatal.2c00710)
Supplement: Supplementary file 1 — cs2c00710_si_001.pdf [file cs2c00710_si_001.pdf]

Supporting information for

# *Interplay between Directing Group and Multifunctional Acetate Ligand in Pd-Catalyzed anti-Acetoxylation of Unsymmetrical Dialkyl-Substituted Alkynes*

Javier Corpas*[a] Enrique M. Arpa,[b] Romain Lapierre[a], Inés Corral,[c,d] Pablo Mauleón*[a,d], Ramón Gómez Arrayás*[a,d] and Juan C. Carretero[a,d].

[a]Departamento de Química Orgánica, Facultad de Ciencias, Módulo 1, Universidad Autónoma de Madrid, Campus de Excelencia UAM-CSIC Cantoblanco, 28049 Madrid (Spain).

[b]Division of Theoretical Chemistry, IFM, Linköping University, 581 83 Linköping, Sweden.

[c]Departamento de Química, Facultad de Ciencias, Módulo 13, Universidad Autónoma de Madrid, Campus de Excelencia UAM-CSIC Cantoblanco, 28049 Madrid (Spain).

[d]Institute for Advanced Research in Chemical Sciences (IAdChem), Universidad Autónoma de Madrid, 28049 Madrid, Spain

*e-mail for J.C..: javier.corpas@uam.es
*e-mail for P.M.: pablo.mauleon@uam.es
*e-mail for R.G.A.: ramon.gomez@uam.es

## CONTENTS

1. General methods. .......... 2
2. Pd-catalyzed acetoxylation of unsymmetrical internal alkynes .......... 2
3. KIE experiments .......... 6
4 Determination of the stereochemistry .......... 8
5 DFT studies .......... 9
6 Unsuccessful substrates .......... 30
7 References .......... 31
8 NMR spectra .......... 32

1. **General methods.**

All reagents and solvents were purchased from commercial sources and used as received. Starting materials were prepared according to previous method reported in the literature.[1,2] All reactions were carried out in anhydrous solvents and air atmosphere, unless otherwise noted. Column liquid chromatographies were performed on silica gel (230-400 mesh ASTM). TLC analysis was performed on 0.2 mm aluminium-based plates (60 230-400 mesh). $^{1}$H, $^{13}$C, and $^{19}$F NMR spectra were recorded in $CDCl_3$ or AcOD-$d_4$ solutions at 25 °C (indicated on each case) on AV-300, AVII-300 y AVIII-HD-300 (300, 75, and 282 MHz, respectively) spectrometers (δ, ppm; J, Hz). $^{1}$H and $^{13}$C NMR spectra were referenced using the solvent signal as internal standard. HRMS electrospray ionization (ESI+) were recorded using an API-QToF ESI with a mass range from 20 to 3000 m/z and mass resolution 15000 (FWHM). Melting points were determined in open-end capillary tubes.

## 2. Pd-catalyzed acetoxylation of unsymmetrical internal alkynes

**General procedure for the acetoxylation of propargyl 2-pyridyl sulfones:**

In a scintillation vial charged with a magnetic stir bar, the corresponding propargyl 2-pyridyl sulfone **1** (0.2 mmol) and $Pd(OAc)_2$ (2.24 mg, 0.01 mmol, 5 mol%) were dissolved in 1.0 mL of acetic acid. The mixture was allowed to stir at room temperature for 10 minutes. Then, the reaction vial was placed into a pre-heated oil bath and stirred at 80-100 °C (indicated in each case). The reaction was followed by TLC analysis after full completion was observed. After that, the reaction was cooled to room temperature, and the residue was filtered off through a pad of silica gel employing AcOEt as eluent. The solvent was removed in vacuo and the resulting residue was further purified by flash column chromatography.

**(*Z*)-4-(pyridin-2-ylsulfonyl)but-2-en-2-yl acetate (*Z*-2a):**

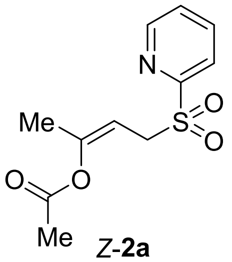


*Z*-**2a**

Following the general procedure, the reaction between the alkyne **1a** (39 mg, 0.2 mmol) and $Pd(OAc)_2$ (2.24 mg, 0.01 mmol) at 80 °C for 1h afforded after purification by flash column chromatography (cyclohexane:AcOEt 2:1) 42.9 mg (84% yield) of the titled compound as a white solid. M.p. = 189-191°C. **$^{1}$H-NMR** (300 MHz, $CDCl_3$): δ 8.73 (d, J = 4.8 Hz, 1H), 8.03 (d, J = 7.8 Hz, 1H),7.94 (td, J = 7.7, 1.3 Hz, 1H), 7.55 (ddd, J = 7.6, 4.8, 1.3 Hz, 1H, HC), 5.05 (t, J = 7.8 Hz, 1H), 4.08 (d, J = 7.7 Hz, 2H), 2.08 (s, 3H), 1.86 (s, 3H). **$^{13}$C-NMR** (75 MHz, $CDCl_3$): δ 168.0, 156.7, 152.7, 150.3, 138.1, 127.6, 123.0, 103.1, 49.7, 20.8, 19.8. **HRMS** (ESI+) m/z $[M + H]^+$ calcd for $C_{11}H_{14}NO_4S$ 256.0638, found 256.0642.

**(*Z*)-5-(pyridin-2-ylsulfonyl)hex-3-en-3-yl acetate (*Z*-4):**

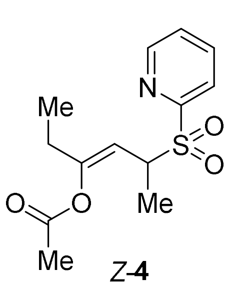


*Z*-**4**

Following the general procedure, the reaction between the alkyne **1b** (44.7 mg, 0.2 mmol) and $Pd(OAc)_2$ (2.24 mg, 0.01 mmol) at 80 °C for 1 h afforded after purification by flash column chromatography (cyclohexane:AcOEt 2:1) 47 mg (83% yield) of the titled compound as a pale yellow oil. **$^{1}$H-NMR** (300 MHz, $CDCl_3$): δ 8.75 (s, 1H), 8.02 (s, 1H), 7.93 (t, J = 7.4 Hz, 1H), 7.54 (s, 1H), 4.97 (d, J = 10.3 Hz, 1H), 4.54 – 4.32 (m, 1H), 2.14 – 2.06 (m, 2H), 2.06 (s, 3H), 1.52 (d, J = 6.9 Hz, 3H), 0.86 (t, J = 7.5 Hz, 3H). **$^{13}$C-NMR** (75 MHz, $CDCl_3$): δ 168.0, 156.4, 155.7, 150.4, 137.7, 127.5, 124.0, 109.1, 54.2, 26.4, 20.8, 12.5, 11.0. **HRMS** (ESI+) m/z $[M + H]^+$ calcd for $C_{13}H_{18}NO_4S$ 284.0951, found 284.0956.

**(*Z*)-5-methyl-2-(pyridin-2-ylsulfonyl)oct-3-en-4-yl acetate (*Z*-5):**

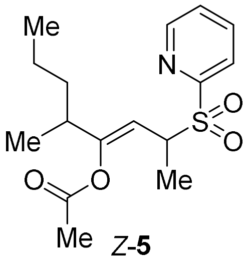
*Z*-**5**

Following the general procedure, the reaction between the alkyne **1c** (53.1 mg, 0.2 mmol) and $Pd(OAc)_2$ (2.24 mg, 0.01 mmol) at 100 °C for 3 h afforded after purification by flash column chromatography (cyclohexane:AcOEt 2:1) 48.1 mg (74% yield) of the titled compound as an oil. Mixture of diastereoisomers (the asterisk denotes the signal for the diastereoisomer) **¹H-NMR** (300 MHz, $CDCl_3$): δ 8.74 (dt, J = 3.5, 1.9 Hz, 1H), 8.06 – 7.93 (m, 1H), 7.95 – 7.87 (m, 1H), 7.53 (ddt, J = 7.4, 4.7, 1.2 Hz, 1H), 4.97 (d, J = 10.6 Hz, 1H), 4.44 – 4.26 (m, 1H), 2.18-2.05 (m, 4H), 1.53* (d, J = 1.4 Hz, 1.5H), 1.51* (d, J = 1.4 Hz, 1.5H), 1.28 – 0.92 (m, 4H), 0.91 – 0.67 (m, 6H). **¹³C-NMR** (75 MHz, $CDCl_3$): δ 168.2*, 168.1, 158.0*, 157.6, 156.5 (common signal), 150.4 (common signal), 137.7*, 137.6, 127.4*, 127.32, 109.2*, 109.0, 54.5*, 54.4, 37.8*, 37.5, 36.5*, 36.4, 20.7*, 20.6, 19.9*, 19.8, 18.0*, 17.9, 14.1*, 14.0, 12.1*, 12.0. **HRMS** (ESI+) m/z $[M + H]^+$ calcd for $C_{16}H_{24}NO_4S$ 326.1421, found 326.1416.

**(*Z*)-6-methyl-2-(pyridin-2-ylsulfonyl)hept-3-en-4-yl acetate (*Z*-6):**

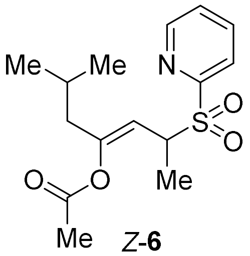
*Z*-**6**

Following the general procedure, the reaction between the alkyne **1d** (50.3 mg, 0.2 mmol) and $Pd(OAc)_2$ (2.24 mg, 0.01 mmol) at 100 °C for 2.5 h afforded after purification by flash column chromatography (cyclohexane:AcOEt 2:1) 44.8 mg (72% yield) of the titled compound as an oil. **¹H-NMR** (300 MHz, $CDCl_3$): δ 8.73 (ddd, J = 4.7, 1.7, 0.9 Hz, 1H), 8.02 (dq, J = 7.8, 0.9 Hz, 1H), 7.91 (td, J = 7.7, 1.7 Hz, 1H), 7.63 – 7.37 (m, 1H), 4.96 (d, J = 10.4 Hz, 1H), 4.50 (dq, J = 10.2, 7.0 Hz, 1H), 2.06 (s, 3H), 1.96 (dd, J = 7.1, 4.5 Hz, 2H), 1.60-1.45 (m, 4H), 0.76 (d, J = 6.6 Hz, 3H), 0.67 (d, J = 6.6 Hz, 3H). **¹³C-NMR** (75 MHz, $CDCl_3$): δ 168.0, 156.5, 153.3, 150.4, 137.8, 127.4, 123.7, 111.2, 54.1, 42.5, 25.9, 22.2, 20.8, 12.6. **HRMS** (ESI+) m/z $[M + H]^+$ calcd for $C_{15}H_{22}NO_4S$ 312.1264, found 312.1260.

**(*Z*)-6-(pyridin-2-ylsulfonyl)non-4-en-4-yl acetate (*Z*-7):**

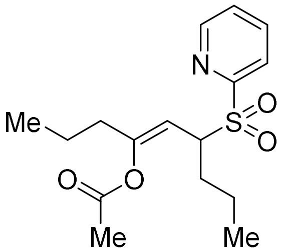
*Z*-**7**

Following the general procedure, the reaction between the alkyne **1e** (53 mg, 0.2 mmol) and $Pd(OAc)_2$ (2.24 mg, 0.01 mmol) at 100 °C for 2 h afforded after purification by flash column chromatography (cyclohexane:AcOEt 2:1) 39.7 mg (61% yield) of the titled compound as an oil. **¹H-NMR** (300 MHz, $CDCl_3$): δ 8.73 (s, 1H), 8.00 (d, J = 6.3 Hz, 1H), 7.91 (t, J = 7.4 Hz, 1H), 7.53 (s, 1H), 4.88 (d, J = 10.5 Hz, 1H), 4.40 (t, J = 9.8 Hz, 1H), 2.14 – 2.05 (m, 2H), 2.03 (s, 3H), 1.82-1.62 (m, 2H), 1.60-1.52 (m, 2H), 1.25 (q, J = 7.4 Hz, 2H), 0.93 (t, J = 7.3 Hz, 3H), 0.76 (t, J = 7.3 Hz, 3H). **¹³C-NMR** (75 MHz, $CDCl_3$): δ 167.9, 155.7, 137.8, 127.4, 123.8, 109.0, 58.6, 35.1, 28.2, 22.4, 20.8, 19.8, 13.8, 13.4. **HRMS** (ESI+) m/z $[M + H]^+$ calcd for $C_{16}H_{24}NO_4S$ 326.1421, found 326.1418.

**(*Z*)-1-cyclohexyl-5,5-dimethyl-3-(pyridin-2-ylsulfonyl)hex-1-en-1-yl acetate (*Z*-8):**

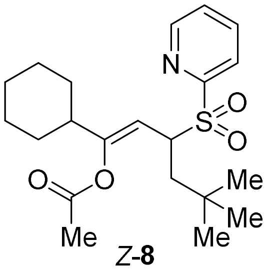
*Z*-**8**

Following the general procedure, the reaction between the alkyne **1f** (66.7 mg, 0.2 mmol) and $Pd(OAc)_2$ (2.24 mg, 0.01 mmol) at 100 °C for 8 h afforded after purification by flash column chromatography (cyclohexane:AcOEt 3:1) 41.7 mg (53% yield) of the titled compound as an oil. **¹H-NMR** (300 MHz, $CDCl_3$): δ 8.74 (ddd, J = 4.6, 1.7, 0.9 Hz, 1H), 7.97 (dt, J = 7.8, 1.2 Hz, 1H), 7.89 (td, J = 7.7, 1.7 Hz, 1H), 7.52 (ddt, J = 7.5, 4.7, 1.5 Hz, 1H), 4.87 (dd, J = 10.4, 1.1 Hz, 1H), 4.38 (td, J = 10.7, 1.8 Hz, 1H), 2.28 (dd, J = 13.8, 1.8 Hz, 1H), 2.02 (s, 3H), 1.79 – 1.41 (m, 7H), 1.18 – 0.97 (m, 4H), 0.94 (s, 9H). **¹³C-NMR** (75 MHz, $CDCl_3$): δ 167.9, 158.8, 156.3, 150.4, 137.5, 127.3, 124.6, 109.5, 56.9, 41.0, 39.3, 31.1, 30.8, 30.6, 29.9, 26.1, 26.0, 20.8. **HRMS** (ESI+) m/z $[M + H]^+$ calcd for $C_{21}H_{32}NO_4S$ 394.2047, found 394.2043.

**(*Z*)-1-phenyl-3-(pyridin-2-ylsulfonyl)non-4-en-5-yl acetate (*Z*-9):**

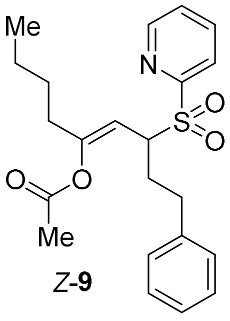

*Z*-**9**

Following the general procedure, the reaction between the alkyne **1g** (68.3 mg, 0.2 mmol) and $Pd(OAc)_2$ (2.24 mg, 0.01 mmol) at 100 °C for 5 h afforded after purification by flash column chromatography (cyclohexane:AcOEt 3:1) 58.6 mg (73% yield) of the titled compound as an oil. **$^1$H-NMR** (300 MHz, $CDCl_3$): δ 8.75 (ddd, *J* = 4.7, 1.7, 0.9 Hz, 1H), 8.05 (dt, *J* = 7.8, 1.1 Hz, 1H), 7.95 (td, *J* = 7.7, 1.7 Hz, 1H), 7.61 – 7.52 (m, 1H), 7.40 – 7.26 (m, 3H), 7.22 (d, *J* = 6.4 Hz, 2H), 4.98 (d, *J* = 10.6 Hz, 1H), 4.46 (td, *J* = 10.6, 3.2 Hz, 1H), 2.87 (dq, *J* = 13.0, 6.8, 5.9 Hz, 1H), 2.70 – 2.55 (m, 2H), 2.19 (t, *J* = 6.6 Hz, 2H), 2.16 – 2.04 (m, 1H), 2.00 (s, 3H), 1.33 – 1.14 (m, 4H), 0.88 (t, *J* = 7.0 Hz, 3H). **$^{13}$C-NMR** (75 MHz, $CDCl_3$): δ 167.8, 156.5, 156.4, 150.4, 140.4, 137.7, 128.7, 128.6, 127.4, 126.3, 123.9, 108.5, 58.2, 32.8, 32.4, 28.6, 27.9, 22.0, 20.7, 13.8. **HRMS** (ESI+) m/z $[M + H]^+$ calcd for $C_{22}H_{28}NO_4S$ 402.1734, found 402.1735.

**(*Z*)-9-chloro-1-phenyl-3-(pyridin-2-ylsulfonyl)non-4-en-5-yl acetate (*Z*-10):**

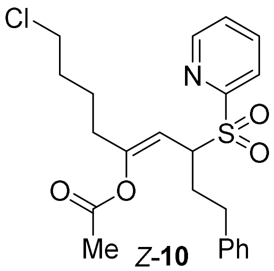

*Z*-**10**

Following the general procedure, the reaction between the alkyne **1h** (75.2 mg, 0.2 mmol) and $Pd(OAc)_2$ (2.24 mg, 0.01 mmol) at 100 °C for 4.5 h afforded after purification by flash column chromatography (cyclohexane:AcOEt 2:1) 53.2 mg (61% yield) of the titled compound as an oil. **$^1$H-NMR** (300 MHz, $CDCl_3$): δ 8.71 (ddd, J = 4.6, 2.3, 1.1 Hz, 1H), 8.01 (dq, J = 7.8, 1.2 Hz, 1H), 7.92 (tt, J = 7.8, 1.4 Hz, 1H), 7.53 (ddt, J = 7.5, 4.7, 1.3 Hz, 1H), 7.30 – 7.25 (m, 2H), 7.23 – 7.15 (m, 3H), 4.97 (d, J = 10.6 Hz, 1H), 4.42 (td, J = 10.6, 2.2 Hz, 1H), 3.46 (t, J = 6.5 Hz, 2H), 2.89 – 2.78 (m, 1H), 2.66 – 2.53 (m, 2H), 2.19 (t, J = 7.5 Hz, 2H), 2.14 – 2.03 (m, 1H), 1.96 (s, 3H), 1.69 – 1.58 (m, 2H), 1.40 (p, J = 7.4 Hz, 2H). **$^{13}$C-NMR** (75 MHz, $CDCl_3$): δ 167.9, 156.5, 155.5, 150.4, 140.3, 128.7, 128.6, 127.5, 126.4, 123.8, 109.2, 58.2, 44.6, 32.4, 32.4, 31.6, 28.0, 23.8, 20.7. **HRMS** (ESI+) m/z $[M + H]^+$ calcd for $C_{22}H_{27}ClNO_4S$ 436.1344, found 436.1342 (Cl 35 was used to determine accurate mass calculation).

**(*Z*)-8-chloro-2-(pyridin-2-ylsulfonyl)oct-3-en-4-yl acetate (*Z*-11):**

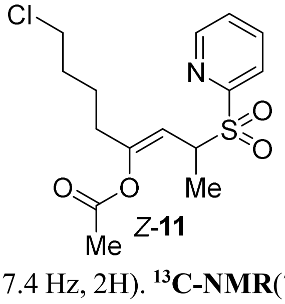

*Z*-**11**

Following the general procedure, the reaction between the alkyne **1i** (57.1 mg, 0.2 mmol) and $Pd(OAc)_2$ (2.24 mg, 0.01 mmol) at 100 °C for 4.5 h afforded after purification by flash column chromatography (cyclohexane:AcOEt 2:1) 54.6 mg (79% yield) of the titled compound as an oil. **$^1$H-NMR** (300 MHz, $CDCl_3$): δ 8.80 – 8.67 (m, 1H), 8.03 (dd, J = 7.8, 1.1 Hz, 1H), 7.93 (td, J = 7.7, 1.6 Hz, 1H), 7.54 (ddd, J = 7.6, 4.7, 1.3 Hz, 1H), 5.00 (d, J = 10.2 Hz, 1H), 4.47 (dq, J = 10.2, 7.0 Hz, 1H), 3.44 (t, J = 6.5 Hz, 2H), 2.13 (t, J = 6.5 Hz, 3H), 2.08 (s, 3H), 1.66 – 1.53 (m, 2H), 1.51 (d, J = 7.0 Hz, 3H), 1.40 (p, J = 7.4 Hz, 2H). **$^{13}$C-NMR**(75 MHz, $CDCl_3$): δ 168.0, 156.4, 153.4, 150.4, 137.9, 127.5, 123.8, 110.7, 54.1, 44.6, 32.5, 31.5, 23.6, 20.7, 12.6. **HRMS** (ESI+) m/z $[M + H]^+$ calcd for $C_{15}H_{21}ClNO_4S$ 346.0874, found 346.0870 (Cl 35 was used to determine accurate mass calculation).

**(*Z*)-7-cyano-2-(pyridin-2-ylsulfonyl)hept-3-en-4-yl acetate (*Z*-12):**

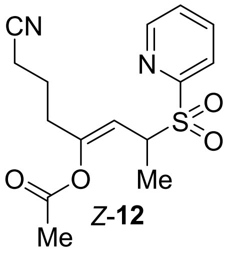


Following the general procedure, the reaction between the alkyne **1j** (52.4 mg, 0.2 mmol) and $Pd(OAc)_2$ (2.24 mg, 0.01 mmol) at 100 °C for 5 h afforded after purification by flash column chromatography (cyclohexane:AcOEt 2:1) 46.4 mg (72% yield) of the titled compound as an oil. **$^1$H-NMR** (300 MHz, $CDCl_3$): δ 8.75 (ddd, J = 4.7, 1.7, 0.9 Hz, 1H), 8.04 (dq, J = 7.8, 1.2 Hz, 1H), 7.95 (td, J = 7.7, 1.8 Hz, 1H), 7.56 (ddt, J = 7.5, 4.7, 1.2 Hz, 1H), 5.08 (d, J = 10.4 Hz, 1H), 4.50-4.25 (m, 1H), 2.35-2-20 (m, 4H), 2.10 (s, 3H), 1.65 (p, J = 7.2 Hz, 2H), 1.48 (d, J = 7.0 Hz, 3H). **$^{13}$C-NMR** (75 MHz, $CDCl_3$): δ 168.1, 156.3, 151.6, 150.5, 138.0, 127.6, 119.0, 112.0, 54.3, 32.1, 22.4, 20.7, 16.1, 12.8. **HRMS** (ESI+) m/z $[M + H]^+$ calcd for $C_{15}H_{19}N_2O_4S$ 323.1060, found 323.1058.

**(*Z*)-1-phenyl-3-(pyridin-2-ylsulfonyl)prop-1-en-1-yl acetate (*Z*-13):**

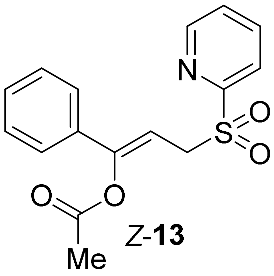


Following the general procedure, the reaction between the alkyne **1k** (51.5 mg, 0.2 mmol) and $Pd(OAc)_2$ (2.24 mg, 0.01 mmol) at 90 °C for 1.5 h afforded after purification by flash column chromatography (cyclohexane:AcOEt 2:1) 43.8 mg (69% yield) of the titled compound as an oil. **$^1$H-NMR** (300 MHz, $CDCl_3$): 8.76 (dq, J = 4.0, 1.3 Hz, 1H), 8.05 (d, J = 7.6 Hz, 1H), 7.99 – 7.86 (m, 1H), 7.55 (ddt, J = 7.5, 4.8, 1.4 Hz, 1H), 7.33 – 7.26 (m, 5H), 5.76 (t, J = 7.9 Hz, 1H), 4.27 (d, J = 7.9 Hz, 2H), 2.22 (s, 3H). δ **$^{13}$C-NMR** (75 MHz, $CDCl_3$): 168.0, 156.6, 152.5, 150.5, 138.2, 133.9, 129.6, 128.8, 127.7, 125.0, 123.2, 103.6, 50.6, 20.7. δ. **HRMS** (ESI+) m/z $[M + H]^+$ calcd for $C_{16}H_{16}NO_4S$ 318.0795, found 318.0798.

.

**(*Z*)-1-(4-methoxyphenyl)-3-(pyridin-2-ylsulfonyl)prop-1-en-1-yl acetate (*Z*-14):**

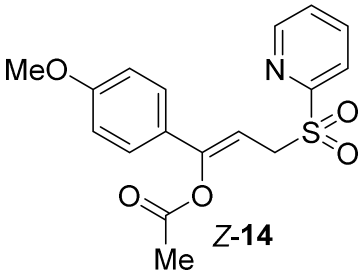


Following the general procedure, the reaction between the alkyne **1l** (57.5 mg, 0.2 mmol) and $Pd(OAc)_2$ (2.24 mg, 0.01 mmol) at 90 °C for 1.5 h afforded after purification by flash column chromatography (cyclohexane:AcOEt 2:1) 41.7 mg (60% yield) of the titled compound as an oil. **$^1$H-NMR** (300 MHz, $CDCl_3$): δ 8.81 – 8.68 (m, 1H), 8.05 (d, J = 7.8 Hz, 1H), 7.93 (td, J = 7.7, 1.6 Hz, 1H), 7.55 (dd, J = 6.9, 4.9 Hz, 1H), 7.21 (d, J = 8.8 Hz, 2H), 6.81 (d, J = 8.8 Hz, 2H), 5.64 (t, J = 7.9 Hz, 1H), 4.24 (d, J = 7.9 Hz, 2H), 3.78 (s, 3H), 2.21 (s, 3H). **$^{13}$C-NMR** (75 MHz, $CDCl_3$): δ 168.0, 160.7, 156.7, 152.3, 150.4, 138.2, 127.7, 126.5, 126.4, 123.2, 114.2, 101.7, 55.5, 50.7, 20.7. **HRMS** (ESI+) m/z $[M + H]^+$ calcd for $C_{17}H_{18}NO_5S$ 348.0900, found 348.0905.

**(*Z*)-1-(4-acetylphenyl)-3-(pyridin-2-ylsulfonyl)but-1-en-1-yl acetate (*Z*-15):**

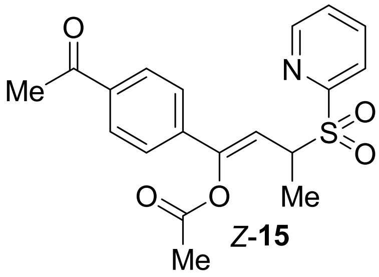


Following the general procedure, the reaction between the alkyne **1m** (62.7 mg, 0.2 mmol) and $Pd(OAc)_2$ (2.24 mg, 0.01 mmol) at 90 °C for 1.5 h afforded after purification by flash column chromatography (cyclohexane:AcOEt 2:1) 38.8 mg (52% yield) of the titled compound as an oil. **$^1$H-NMR** (300 MHz, $CDCl_3$): δ 8.84 – 8.72 (m, 1H), 8.04 (d, J = 7.7 Hz, 1H), 7.95 – 7.82 (m, 3H), 7.57 – 7.50 (m, 1H), 7.31 (d, J = 8.3 Hz, 2H), 5.77 (d, J = 10.5 Hz, 1H), 4.74 – 4.57 (m, 1H), 2.56 (s, 3H), 2.20 (s, 3H), 1.64 (d, J = 7.0 Hz, 3H). **$^{13}$C-NMR** (75 MHz, $CDCl_3$): δ 197.3, 167.8, 156.3, 150.6, 149.9, 138.2, 138.0, 137.5, 128.8, 127.7, 125.2, 123.8, 113.3, 55.0, 26.8, 20.6, 12.6. **HRMS** (ESI+) m/z $[M + H]^+$ calcd for $C_{19}H_{20}NO_5S$ 374.1057, found 374.1061.

**(*Z*)- 3-(pyridin-2-ylsulfonyl)-1-(4-(trifluoromethyl)phenyl)prop-1-en-1-yl acetate (*Z*-16):**

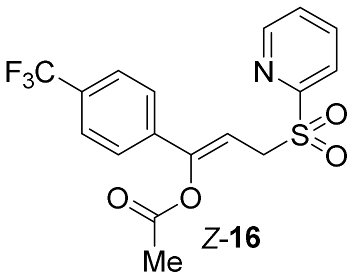

*Z*-**16**

Following the general procedure, the reaction between the alkyne **1n** (65 mg, 0.2 mmol) and $Pd(OAc)_2$ (2.24 mg, 0.01 mmol) at 90 °C for 1.5 h afforded after purification by flash column chromatography (cyclohexane:AcOEt 2:1) 38.5 mg (50% yield) of the titled compound as an oil. **$^1$H-NMR** (300 MHz, $CDCl_3$): δ 8.77 (ddd, J = 4.7, 1.6, 0.9 Hz, 1H), 8.07 (dt, J = 7.9, 1.1 Hz, 1H), 7.96 (td, J = 7.9, 1.6 Hz, 1H), 7.61 – 7.54 (m, 3H), 7.40 (d, J = 8.1 Hz, 2H), 5.86 (t, J = 7.9 Hz, 1H), 4.31 (d, J = 7.9 Hz, 2H), 2.25 (s, 3H).**$^{13}$C-NMR** (75 MHz, $CDCl_3$): δ (the *ipso* to fluorine carbon does not appear) 167.9, 156.7, 151.3, 150.5, 138.3, 137.5, 131.4 (q, J = 32.6 Hz), 127.82, 125.9 (q, J = 3.8 Hz), 125.4, 123.0, 106.1, 50.4, 20.8. $^{19}$F-NMR (282 MHz, $CDCl_3$): δ -62.9. **HRMS** (ESI+) m/z $[M + H]^+$ calcd for $C_{17}H_{15}F_3NO_4S$ 386.0668, found 386.0673.

## 3. KIE experiments

The determination of the KIE was performed by comparison of the reaction rate of propargyl sulfone **1a** in either acetic acid or acetic acid-$d_4$ taking aliquots of the reaction at the indicated time (Figure S1). The reaction kinetics was performed on a 0.4 mmol scale of **1a** taking aliquots of 0.1 mL at the indicated time. The resulting sample was further passed through a pad of silica gel and 0.1 mL of a 0.067 M stock solution of 1,3,5-trimethoxybenzene (TMB) in $CH_2Cl_2$ was added. After that, the solvent was evaporated in vacuo and the mixture was analyzed by $^1$H NMR spectroscopy employing $CDCl_3$ as the solvent. The conversion of starting material and yield of acetoxylation product **2a** were determined by comparing the integrals taking the singlet at 6 ppm from TMB as the reference (see Table S1 for the data).

**Table S1. Kinetic data for the determination of KIE.**

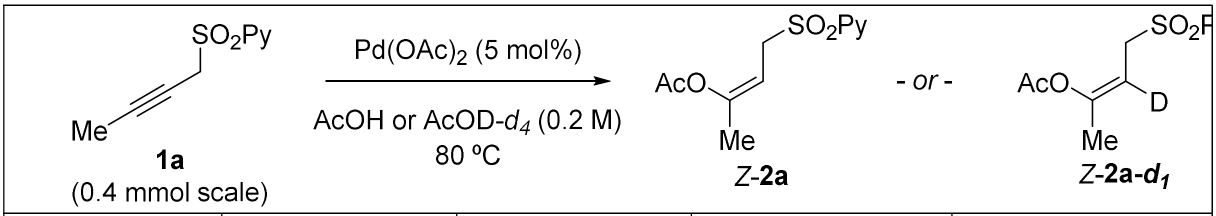


| Time (min) | Conversion 1a (for AcOH) | Conversion 1a (for AcOD-d4) | ln(100-convH) | ln(100-convD) |
|---|---|---|---|---|
| 5 | 21.78 | 10.45 | 4,35952537 | 4,49479713 |
| 10 | 40.40 | 14.26 | 4,08765557 | 4,45131946 |
| 15 | 53.26 | 17.35 | 3,84460033 | 4,41461482 |
| 20 | 64.56 | 20.52 | 3,56784113 | 4,37550542 |
| 25 | 74.17 | 23.91 | 3,25153661 | 4,33191685 |
| 30 | 79.52 | 27.05 | 3,0194488 | 4,28977427 |
| 35 | - | 30.27 | - | 4,24463064 |
| 40 | 83.29 | 33.71 | 2,81600734 | 4,19403906 |
| 45 | - | 36.44 | - | 4,15198434 |
| 50 | 89.05 | 40.19 | 2,39333946 | 4,09117287 |
| 55 | - | 41.85 | - | 4,06302588 |
| 60 | 92.48 | 45.23 | 2,01756614 | 4,0031426 |
| 65 | - | 47.89 | - | 3,95335687 |
| 70 | - | 49.17 | - | 3,92848673 |
| 75 | - | 53.94 | - | 3,82994489 |
| 80 | 99 | 56.6 | 0 | 3,77045944 |

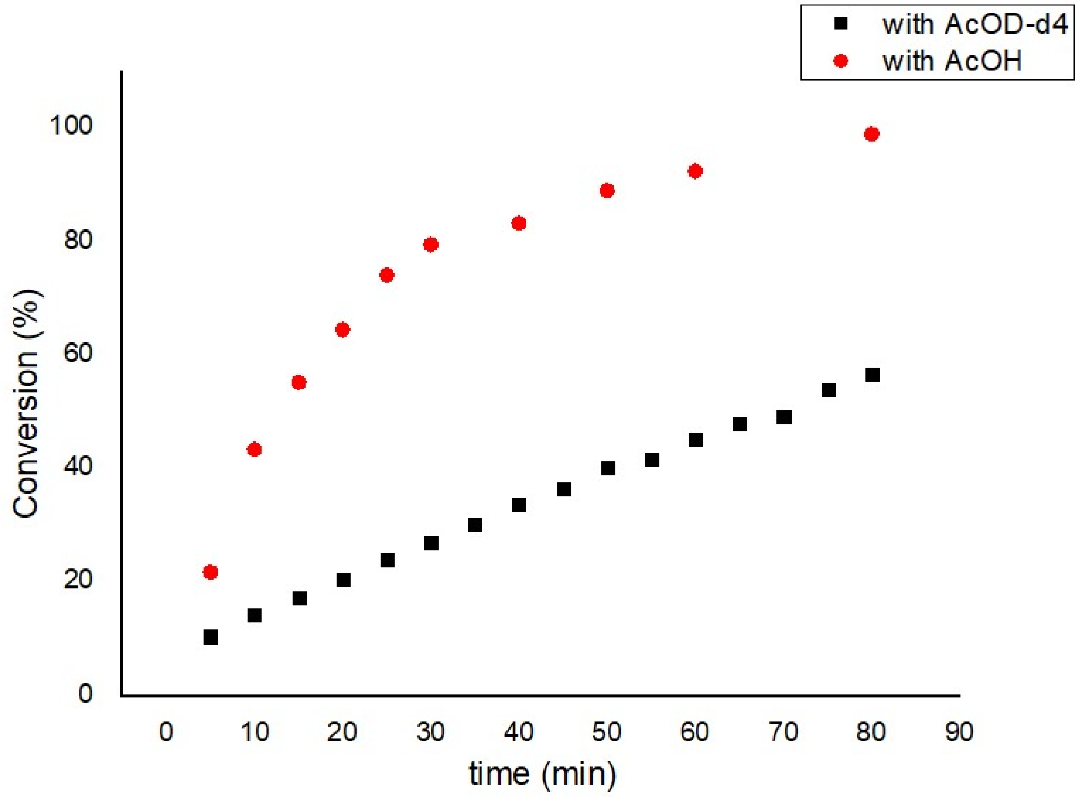


**Figure S1. Reaction conversions for acetoxylation of propargyl sulfone 1a in acetic acid (red) and acetic acid-*d₄*.**

Plotting ln(100-conv) vs time for each kinetic data (Figure S2), the corresponding k-values for the H- and the D-experiments can be obtained:

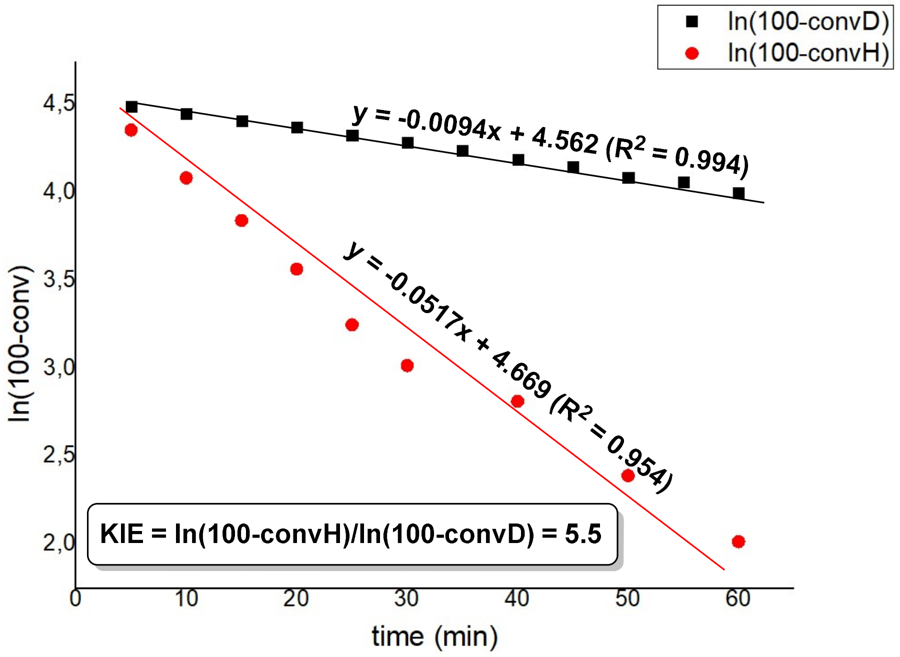


**Figure S2. Determination of KIE for acetoxylation in AcOH (red) and AcOD-$d_4$ (black).**

***Linear adjustment:***

Reaction in AcOH: y = -0.0517x + 4.669 ($R^2$ = 0.954)

Reaction in AcOD-$d_4$: y = -0.0094x + 4.562 ($R^2$ = 0.994)
Thus, the KIE value in this case is given by the expression:

$$KIE = \frac{k_H}{k_D} = \frac{-0.0517}{-0.0094} = \approx 5.5$$

## 4 Determination of the stereochemistry

The configuration of the alkene *Z*-**3a** was determined by performing quantitative nOe experiments. The corresponding correlation between the olefinic proton and allylic protons is given:

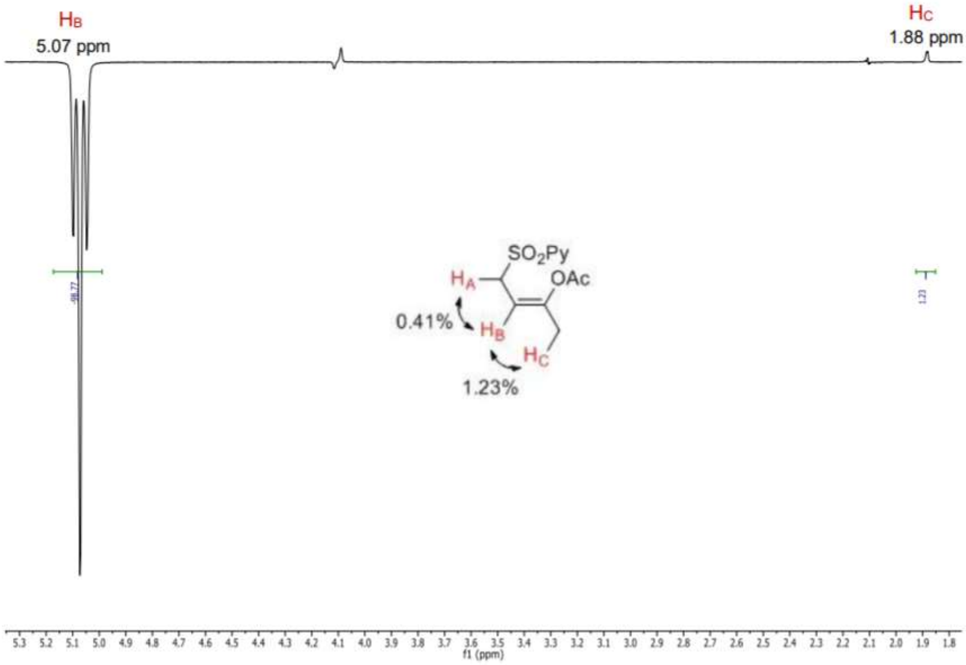


**Figure S3. Correlation between HB and HC by nOe experiment. The HB proton was irradiated showing a correlation of 1.23% with HC.**

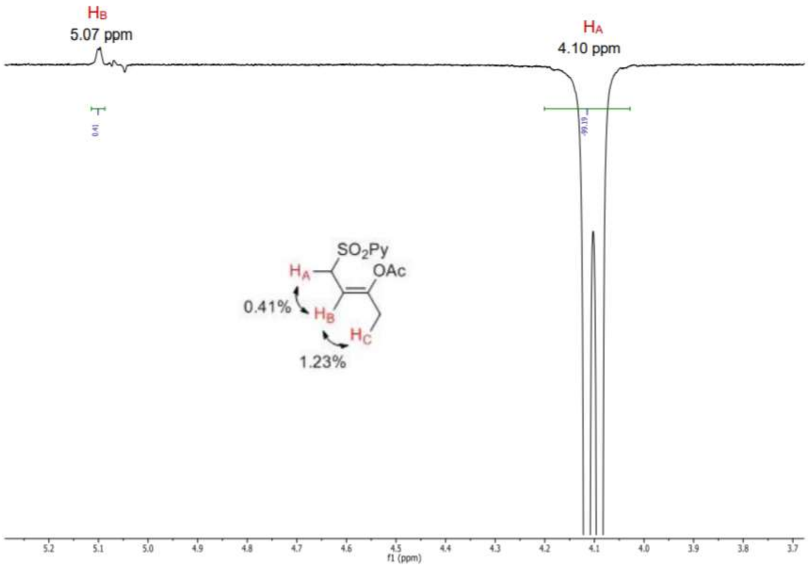


**Figure S4. Correlation between HB and HA by nOe experiment. The HA proton was irradiated showing a correlation of 0.41% with HB.**

# 5 DFT studies

## 5.1. Computational Details

All calculations have been carried out within the framework of density functional theory, using *Gaussian 09* package.[3] In particular, gas-phase geometry optimizations and frequency analysis, the meta-GGA M06-L exchange-correlation functional[4] was employed, in combination with the cc-pVDZ basis set[5] for C, H, N, O and S atoms, and the def2-SVP basis set and ECP[6] for Pd. More accurate electronic energies were obtained through single-point energy calculations at the M06-L/cc-pVTZ(C,H,N,O,S),def2-TZVP(Pd) level of theory, including solvent effects (AcOH) using the SMD model.[7]
The condensed Fukui functions[8] can be used to estimate the relative electrophilicity or nucleophilicity of a given atomic position in a molecule. In contrast to the "global" Fukui functions, which are obtained from the difference between the unperturbed electron density (no additional electrons) and the electron density upon addition/removal of one electron, condensed Fukui functions come from the variation in the atomic charges (populations) under the same circumstances. Therefore, to evaluate the electrophilicity of the Cα and Cβ positions of propargyl sulfone **1a**, NBO analysis allowed us to calculate their population variation upon addition of an extra electron, $f^-_i = P_i\,(N+1) - P_i\,(N) = q_i\,(N) - q_i\,(N+1)$. Larger values of $f^-_i$ are related to larger electrophilicities.

## 5.2. Electrophilicity of Cα and Cβ positions

We have calculated the condensed Fukui functions at the Cα and Cβ positions ($f^-_\alpha$ and $f^-_\beta$) to qualitatively assign the intrinsic reactivity of the triple bond in different scenarios (Table S1). We considered the free propargyl sulfone **I1**, the **I1**-$Pd(OAc)_2$ complex (**I2**, with and without Pd-alkyne bond), and **I2** with one or two additional AcOH molecules (linked by hydrogen bonds to the acetate ligands). We also evaluated the final adducts **I7** and **I8** (see Figure 2 of the main text), to assess if a second acetoxylation is feasible given that there exists a Pd-alkene bond in those structures. These results (see below) show that formation of a Pd-alkyne bond is critical to increase the electrophilicity of the triple bond, and that subsequent coordination of AcOH molecules slightly rises it. On the other hand, the lower electrophilicity of the double bond prevents diacetoxylation.

**Table S1.**

| Molecule | $f^-_\alpha$ | $f^-_\beta$ |
|---|---|---|
| **I1** | -0.023 | +0.031 |
| **I2** (Pd-sulfone bond) | -0.018 | +0.032 |
| **I2** (Pd-alkyne bond) | +0.028 | +0.051 |
| **I2** + 1 AcOH | +0.034 | +0.053 |
| **I2** + 2 AcOH | +0.039 | +0.058 |
| **I7** | +0.043 | +0.041 |
| **I8** | +0.045 | +0.043 |

## 5.3. Evaluation of alternative reaction pathways

Apart from intermolecular AcOH α- and β-addition, we also calculated three additional pathways. One of them is the intramolecular *syn* insertion of the alkyne into the Pd-OAc bond, which would lead to the β-adduct but with opposite stereochemistry, and unable to give an intramolecular protodepalladation (Figure S5). The other pathways are also intermolecular AcOH additions but considering a second AcOH molecule linked to the complex (see below, AcOH marked with dashed circles). None of these pathways are more favorable than the intermolecular β-addition with just one AcOH, either due to larger activation barriers or endergonic coordination of the second AcOH molecule.

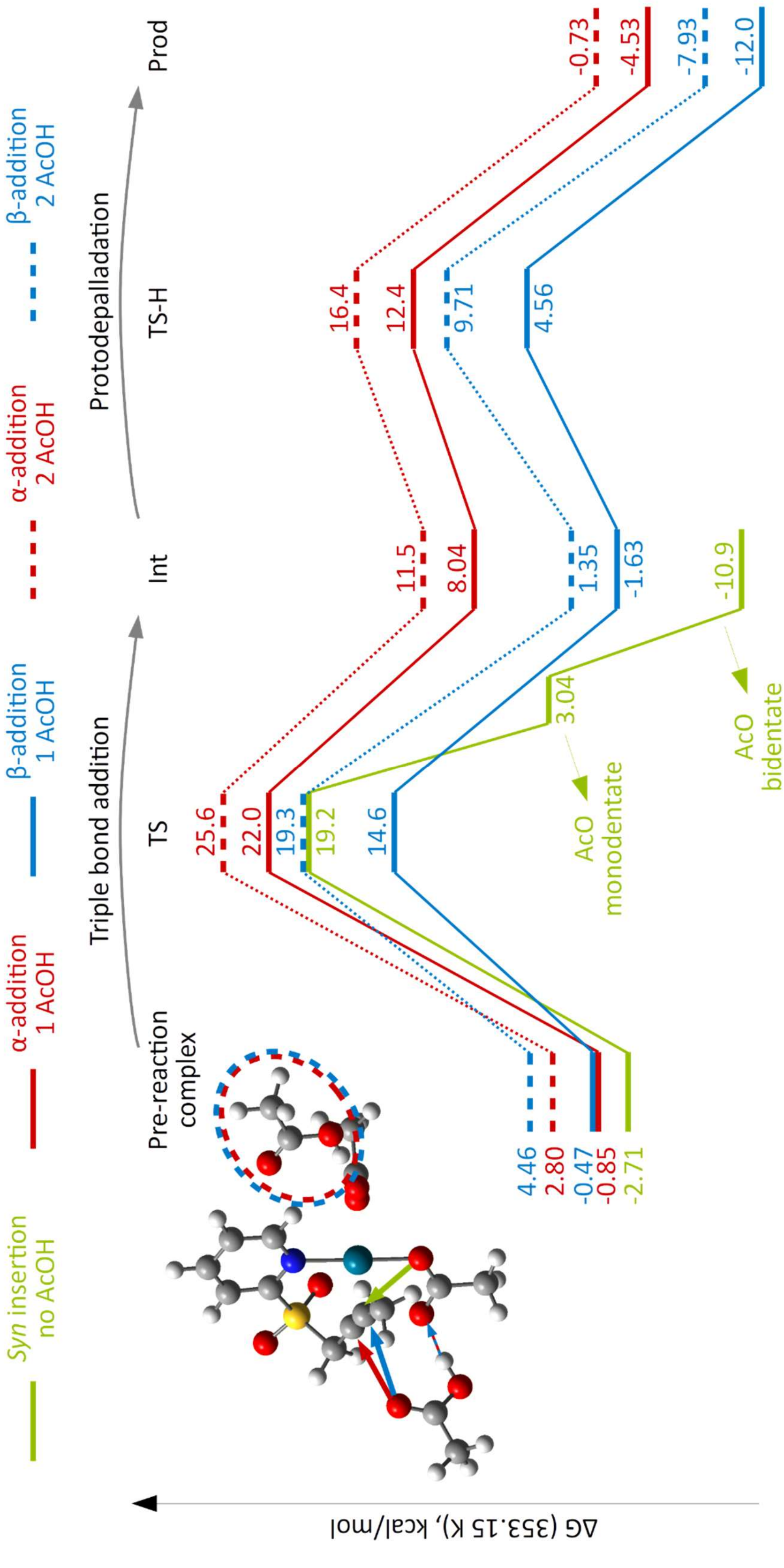


**Figure S5.** Alternative pathways.

## 5.4. IRC analysis with the Distortion/Interaction model

In the main text, we have presented the distortion-interaction analysis for the IRC of the acetoxylation considering one AcOH molecule. We also calculated the reaction pathways including an additional AcOH molecule, linked through hydrogen bonding to the non-participating AcO ligand. Thus, this second AcOH molecule should account for explicit solute-solvent interaction. In the previous section, we showed that this additional AcOH does not have any substantial effect on the activation barriers, and that the energy profile is just up-shifted due to entropy loss. Furthermore, identical conclusions can be extracted if the distortion-interaction analysis is carried out for these reaction pathways considering 2 AcOH molecules, as shown below (Figure S6). In this case, [Pd] stands for **1a**-Pd(OAc)$_2$-AcOH (the AcOH that does not participate in the reaction).

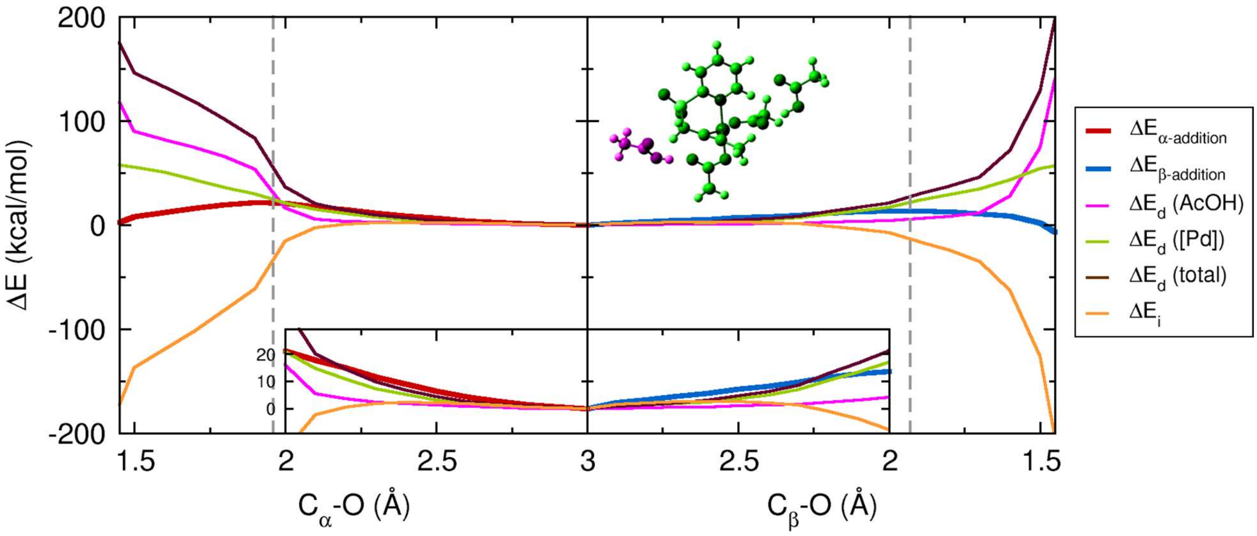


**Figure S6.** Distortion-interaction model considering 2 molecules of AcOH.

## 5.5. Geometries of minima and transition states

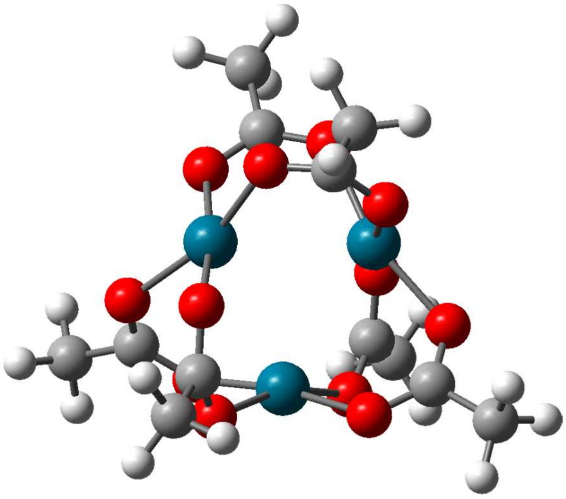

Pd3OAc6.xyz E=-1755.45824312 au

| | | | |
|---|---|---|---|
| Pd | -1.624472 | 0.744780 | -0.001142 |
| O | -2.221592 | -0.178795 | 1.729947 |
| C | -2.136163 | -1.435004 | 1.882403 |
| O | -1.469377 | -2.244029 | 1.173398 |
| C | -2.890207 | -2.027447 | 3.035584 |
| H | -2.182052 | -2.224381 | 3.851732 |

| | | | |
|---|---|---|---|
| H | -3.666927 | -1.345921 | 3.395127 |
| H | -3.325747 | -2.989916 | 2.745341 |
| O | -2.604237 | -0.639281 | -1.180818 |
| C | -2.059929 | -1.535184 | -1.889692 |
| O | -0.900004 | -2.025899 | -1.740176 |
| C | -2.872326 | -2.057857 | -3.036976 |
| H | -3.912004 | -2.202718 | -2.722487 |
| H | -2.878793 | -1.304617 | -3.835875 |
| H | -2.456584 | -2.991766 | -3.426505 |
| O | -1.309850 | 1.794734 | -1.732617 |
| C | -0.304479 | 2.554777 | -1.878001 |
| O | 0.742214 | 2.577158 | -1.166356 |
| C | -0.348416 | 3.521809 | -3.023469 |
| H | 0.308709 | 3.151704 | -3.821557 |
| H | -1.364145 | 3.630563 | -3.414746 |
| H | 0.046916 | 4.493283 | -2.706132 |
| O | -1.205681 | 2.389392 | 1.178561 |
| C | -0.173025 | 2.555835 | 1.891308 |
| O | 0.955054 | 1.994287 | 1.743160 |
| C | -0.307135 | 3.508579 | 3.041509 |
| H | -0.879197 | 4.390232 | 2.731613 |
| H | -0.878410 | 3.016862 | 3.840204 |
| H | 0.672162 | 3.803388 | 3.429870 |
| Pd | 0.164320 | -1.773340 | -0.005969 |
| Pd | 1.458718 | 1.031227 | 0.005122 |
| O | 1.256321 | -1.835903 | 1.728164 |
| C | 2.306006 | -1.140691 | 1.881403 |
| O | 2.677008 | -0.156910 | 1.176506 |
| C | 3.199078 | -1.508727 | 3.028582 |
| H | 3.054961 | -0.777770 | 3.835224 |
| H | 2.970030 | -2.509653 | 3.406086 |
| H | 4.248613 | -1.445321 | 2.719842 |
| O | 2.218039 | 0.245610 | -1.729483 |
| C | 2.368063 | -1.004104 | -1.886175 |
| O | 1.858567 | -1.925772 | -1.184362 |
| C | 3.227857 | -1.442781 | -3.034196 |
| H | 2.578934 | -1.822950 | -3.834426 |
| H | 3.828323 | -0.614323 | -3.420971 |
| H | 3.873525 | -2.271334 | -2.721888 |

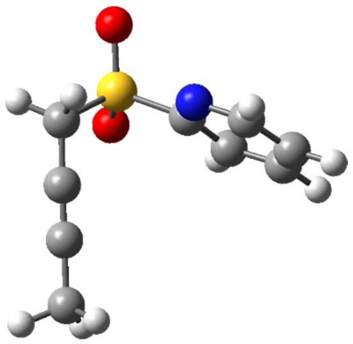

22
I1.xyz E=-951.793881842 au

| | | | |
|---|---|---|---|
| C | -3.065952 | 2.731794 | 0.268594 |
| H | -4.126071 | 2.847023 | 0.002025 |
| H | -2.519872 | 3.575857 | -0.175403 |
| H | -2.991641 | 2.825395 | 1.361083 |
| C | -2.545990 | 1.460222 | -0.185384 |
| C | -2.124325 | 0.384878 | -0.564859 |
| C | -1.588398 | -0.879586 | -0.990476 |
| H | -2.334901 | -1.686462 | -1.034712 |
| H | -1.063841 | -0.831492 | -1.955573 |
| S | -0.324987 | -1.533324 | 0.172575 |
| O | -0.845245 | -1.505788 | 1.556369 |
| O | 0.196021 | -2.776197 | -0.432866 |
| C | 0.945553 | -0.235862 | 0.058505 |
| C | 1.448668 | 0.312703 | 1.232113 |
| N | 1.319005 | 0.082697 | -1.176817 |
| C | 2.453210 | 1.268904 | 1.099034 |
| H | 1.053829 | -0.005442 | 2.197345 |
| C | 2.274038 | 1.013599 | -1.281261 |
| C | 2.873333 | 1.625994 | -0.179931 |
| H | 2.895762 | 1.734398 | 1.981979 |
| H | 2.573534 | 1.280449 | -2.298916 |
| H | 3.652933 | 2.374603 | -0.327339 |

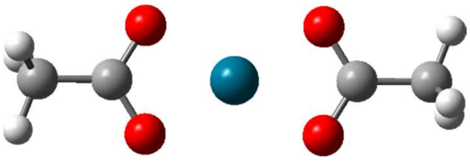

15
PdOAc2.xyz E=-585.111203079 au

| | | | |
|---|---|---|---|
| Pd | 0.000000 | 0.000000 | 0.000085 |
| O | 0.000000 | 2.083062 | -0.000174 |
| C | -1.277655 | 2.080980 | 0.000201 |
| O | -1.855424 | 0.942193 | -0.000003 |
| O | -0.000000 | -2.083062 | -0.000174 |
| C | 1.277655 | -2.080980 | 0.000201 |
| O | 1.855424 | -0.942193 | -0.000003 |
| C | 2.052034 | -3.345525 | -0.000165 |
| H | 3.127144 | -3.142124 | -0.000071 |
| H | 1.787333 | -3.942367 | 0.881653 |
| H | 1.787441 | -3.941936 | -0.882321 |
| C | -2.052034 | 3.345525 | -0.000165 |
| H | -3.127144 | 3.142124 | -0.000071 |
| H | -1.787333 | 3.942367 | 0.881653 |
| H | -1.787441 | 3.941936 | -0.882321 |

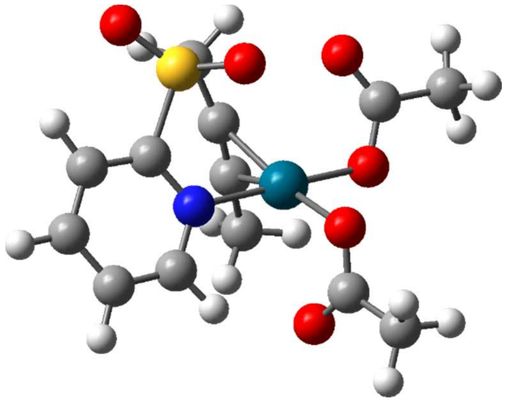

37

I2.xyz E=-1536.94062190 au

| | | | |
|---|---|---|---|
| C | 1.199886 | -0.090330 | 3.126432 |
| H | 1.062377 | -0.625474 | 4.076684 |
| H | 2.272764 | -0.045059 | 2.893335 |
| H | 0.848647 | 0.943624 | 3.249066 |
| C | 0.489810 | -0.760482 | 2.051935 |
| C | -0.089732 | -1.516537 | 1.248338 |
| C | -0.926454 | -2.477762 | 0.566992 |
| H | -1.661139 | -2.959717 | 1.226780 |
| H | -0.316530 | -3.219883 | 0.030588 |
| S | -1.915619 | -1.690202 | -0.782860 |
| O | -3.183412 | -2.439615 | -0.896268 |
| O | -1.037440 | -1.447321 | -1.939814 |
| C | -2.351454 | -0.043890 | -0.109494 |
| C | -3.693315 | 0.276137 | 0.022640 |
| N | -1.352434 | 0.823833 | 0.115059 |
| C | -4.016551 | 1.576969 | 0.406658 |
| H | -4.443864 | -0.485888 | -0.187350 |
| C | -1.664323 | 2.078353 | 0.490587 |
| C | -2.988265 | 2.484790 | 0.639205 |
| H | -5.060507 | 1.874406 | 0.518778 |
| H | -0.803421 | 2.731630 | 0.666128 |
| H | -3.197450 | 3.511653 | 0.939207 |
| Pd | 0.657584 | 0.169380 | 0.104182 |
| O | 1.217880 | 1.559550 | -1.319219 |
| C | 1.491623 | 2.679316 | -0.719757 |
| O | 1.291279 | 2.901922 | 0.483522 |
| O | 2.586728 | -0.446887 | 0.158190 |
| C | 2.739746 | -1.682263 | -0.257079 |
| O | 1.832357 | -2.451949 | -0.561261 |
| C | 2.110573 | 3.714068 | -1.624585 |
| H | 3.190095 | 3.519820 | -1.690756 |
| H | 1.969437 | 4.718362 | -1.210465 |
| H | 1.708698 | 3.655098 | -2.642748 |
| C | 4.193323 | -2.075949 | -0.359830 |
| H | 4.561724 | -1.790748 | -1.354892 |
| H | 4.293902 | -3.162190 | -0.260824 |
| H | 4.815016 | -1.560831 | 0.380977 |

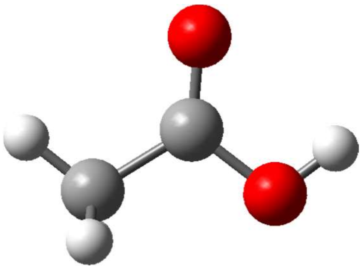

8
AcOH.xyz E=-229.141289535 au

| | | | |
|---|---|---|---|
| C | -0.091520 | 0.127464 | 0.000042 |
| O | -0.653584 | 1.196928 | -0.000015 |
| O | -0.762220 | -1.052681 | -0.000017 |
| H | -1.704837 | -0.814270 | 0.000044 |
| C | 1.385255 | -0.098668 | -0.000032 |
| H | 1.678286 | -0.684563 | 0.880496 |
| H | 1.678061 | -0.686690 | -0.879196 |
| H | 1.912521 | 0.858777 | -0.001150 |

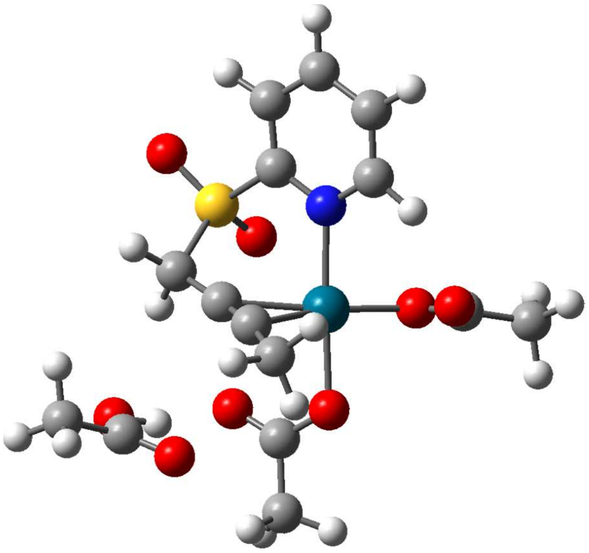

45
I3.xyz E=-1766.10107182 au

| | | | |
|---|---|---|---|
| C | 0.900837 | -2.074499 | -0.411294 |
| C | 0.871363 | -0.936061 | 0.478774 |
| C | -1.961709 | -1.844827 | -0.002836 |
| H | 1.600234 | -1.918339 | -1.245723 |
| C | -2.899035 | -2.784116 | 0.397619 |
| H | -2.816589 | -3.806896 | 0.029048 |
| C | -2.964340 | -0.153335 | 1.210021 |
| H | -2.917908 | 0.902828 | 1.494942 |
| C | -3.949941 | -1.031497 | 1.653881 |
| H | -4.730632 | -0.660022 | 2.317729 |
| C | -3.919510 | -2.362043 | 1.248876 |
| H | -4.680914 | -3.066615 | 1.587286 |
| N | -1.980967 | -0.560677 | 0.385261 |
| H | 1.125427 | -3.018375 | 0.105625 |
| S | -0.721607 | -2.332474 | -1.256845 |
| O | -0.833000 | -1.357944 | -2.356422 |

| | | | |
|---|---|---|---|
| O | -0.896518 | -3.783265 | -1.469457 |
| Pd | -0.399655 | 0.749382 | -0.063391 |
| C | 0.925031 | -0.138987 | 1.433954 |
| C | 1.216626 | 0.678214 | 2.595860 |
| H | 1.905030 | 0.145545 | 3.266618 |
| H | 1.703331 | 1.611066 | 2.282051 |
| H | 0.299722 | 0.934602 | 3.144295 |
| O | 3.946410 | 0.360769 | 1.176559 |
| C | 4.225797 | -0.746165 | 0.752845 |
| O | 3.982001 | -1.156335 | -0.495391 |
| C | 4.856525 | -1.829321 | 1.574891 |
| H | 4.101631 | -2.596047 | 1.803092 |
| H | 5.656461 | -2.332452 | 1.018370 |
| H | 5.242890 | -1.423135 | 2.514133 |
| C | -2.150176 | 2.902371 | -0.089369 |
| O | -2.007129 | 2.762321 | 1.132762 |
| O | -1.660709 | 2.097186 | -0.986300 |
| C | -2.912029 | 4.063277 | -0.674732 |
| H | -2.194940 | 4.850085 | -0.946489 |
| H | -3.442991 | 3.779266 | -1.590534 |
| H | -3.607951 | 4.474928 | 0.064069 |
| O | 1.098209 | 2.087016 | -0.368517 |
| C | 2.125421 | 1.660013 | -1.030822 |
| O | 2.223530 | 0.525955 | -1.531012 |
| H | 3.472425 | -0.433907 | -0.966536 |
| C | 3.243249 | 2.659976 | -1.129228 |
| H | 3.974501 | 2.409560 | -0.347413 |
| H | 3.739605 | 2.571368 | -2.102270 |
| H | 2.896291 | 3.685662 | -0.968012 |

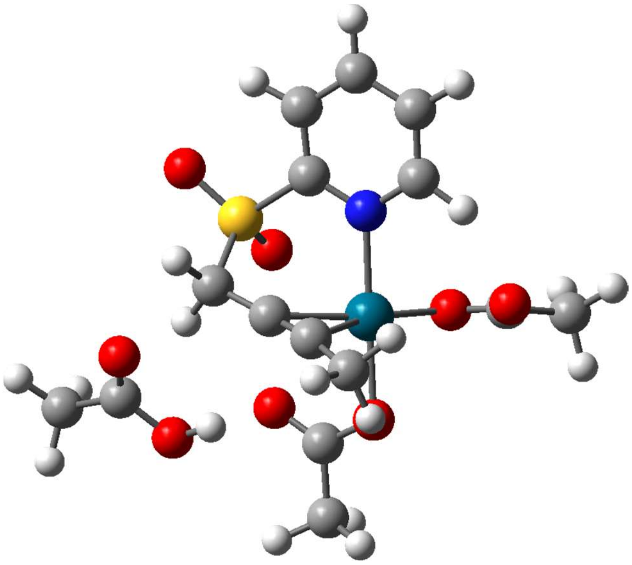

45

I4.xyz E=-1766.09987956 au

| | | | |
|---|---|---|---|
| C | -1.270417 | 1.404766 | 1.202742 |
| C | -0.382859 | 0.363386 | 1.666703 |
| C | 1.146536 | 2.267640 | -0.118771 |
| H | -2.275041 | 1.033492 | 0.915219 |

| | | | |
|---|---|---|---|
| C | 1.754733 | 3.512877 | -0.148772 |
| H | 1.129546 | 4.402160 | -0.228805 |
| C | 3.171149 | 1.159988 | 0.034176 |
| H | 3.661094 | 0.183791 | 0.106780 |
| C | 3.858168 | 2.370444 | 0.003480 |
| H | 4.947008 | 2.363409 | 0.052731 |
| C | 3.146236 | 3.562478 | -0.086757 |
| H | 3.665141 | 4.522270 | -0.111416 |
| N | 1.826391 | 1.113850 | -0.030357 |
| H | -1.361208 | 2.230243 | 1.922679 |
| S | -0.672300 | 2.187618 | -0.350122 |
| O | -0.907763 | 1.274882 | -1.482132 |
| O | -1.138610 | 3.588363 | -0.357552 |
| Pd | 0.819841 | -0.714340 | 0.196246 |
| C | 0.383933 | -0.387815 | 2.299535 |
| C | 1.099578 | -1.244890 | 3.228278 |
| H | 2.186387 | -1.112351 | 3.136558 |
| H | 0.805884 | -1.020015 | 4.263410 |
| H | 0.877801 | -2.300134 | 3.017557 |
| C | 2.836817 | -2.007064 | -1.212604 |
| O | 3.393086 | -1.898372 | -0.111274 |
| O | 1.674396 | -1.503208 | -1.506725 |
| C | 3.467012 | -2.776304 | -2.345616 |
| H | 3.132428 | -3.821300 | -2.286193 |
| H | 3.161265 | -2.389028 | -3.323968 |
| H | 4.558415 | -2.767806 | -2.249959 |
| O | -4.142664 | 1.051552 | 0.264695 |
| C | -4.842859 | 0.298931 | -0.395027 |
| O | -4.492281 | -0.924891 | -0.772754 |
| C | -6.212698 | 0.662977 | -0.881596 |
| H | -6.934790 | -0.122492 | -0.625514 |
| H | -6.528208 | 1.620606 | -0.458543 |
| H | -6.203547 | 0.736362 | -1.977371 |
| O | -0.126680 | -2.509108 | 0.459425 |
| C | -1.400375 | -2.426224 | 0.238503 |
| O | -1.993734 | -1.361303 | 0.005447 |
| H | -3.553817 | -1.103444 | -0.466184 |
| C | -2.122854 | -3.745952 | 0.252450 |
| H | -3.147362 | -3.614861 | 0.620350 |
| H | -2.185926 | -4.116255 | -0.780217 |
| H | -1.594292 | -4.498575 | 0.846669 |

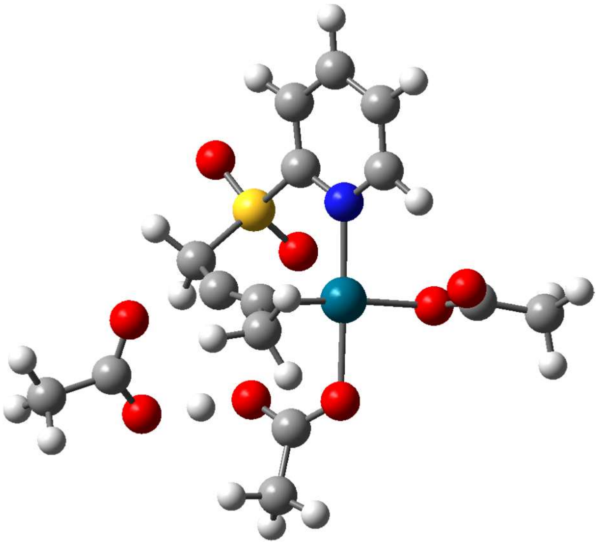

45

TS-α.xyz E=-1766.06261803 au

| | | | |
|---|---|---|---|
| C | -1.402101 | 1.801921 | -0.572956 |
| C | -1.407394 | 0.819281 | 0.493940 |
| C | 1.475198 | 2.068924 | -0.063718 |
| H | -2.037287 | 1.509458 | -1.424627 |
| C | 2.140987 | 3.238041 | 0.269395 |
| H | 1.829273 | 4.174834 | -0.192008 |
| C | 2.829916 | 0.775419 | 1.294529 |
| H | 3.052935 | -0.241511 | 1.633912 |
| C | 3.547553 | 1.904155 | 1.681543 |
| H | 4.375717 | 1.789435 | 2.380981 |
| C | 3.199320 | 3.151114 | 1.172975 |
| H | 3.749549 | 4.047394 | 1.463702 |
| N | 1.799681 | 0.864348 | 0.430664 |
| H | -1.732229 | 2.782177 | -0.203978 |
| S | 0.216279 | 2.138619 | -1.389999 |
| O | 0.514038 | 1.046531 | -2.334290 |
| O | 0.170689 | 3.551537 | -1.821612 |
| Pd | 0.630039 | -0.774986 | 0.045066 |
| C | -0.879324 | -0.048573 | 1.263638 |
| C | -1.217258 | -0.765999 | 2.505985 |
| H | -0.428559 | -0.641326 | 3.261521 |
| H | -2.164546 | -0.392088 | 2.919840 |
| H | -1.299310 | -1.846972 | 2.323772 |
| C | 2.921936 | -2.374207 | -0.127932 |
| O | 2.848959 | -2.246487 | 1.103298 |
| O | 2.145342 | -1.782212 | -0.981894 |
| C | 3.944652 | -3.284658 | -0.762799 |
| H | 3.475448 | -4.259065 | -0.958055 |
| H | 4.292328 | -2.894299 | -1.726349 |
| H | 4.789079 | -3.444169 | -0.083294 |
| O | -3.317304 | 1.271986 | 1.067238 |
| C | -4.261141 | 0.620996 | 0.527651 |
| O | -4.181744 | -0.262804 | -0.379967 |
| C | -5.653802 | 0.935238 | 1.011371 |
| H | -6.105786 | 0.027952 | 1.433365 |

| | | | |
|---|---|---|---|
| H | -5.654753 | 1.729674 | 1.763610 |
| H | -6.279075 | 1.227977 | 0.158249 |
| O | -0.622365 | -2.457373 | -0.223225 |
| C | -1.777970 | -2.259419 | -0.665481 |
| O | -2.101554 | -1.171347 | -1.278205 |
| H | -3.066511 | -0.760007 | -0.920489 |
| C | -2.822246 | -3.309507 | -0.465688 |
| H | -3.642924 | -2.877960 | 0.125402 |
| H | -3.254172 | -3.597192 | -1.432565 |
| H | -2.417636 | -4.188323 | 0.043845 |

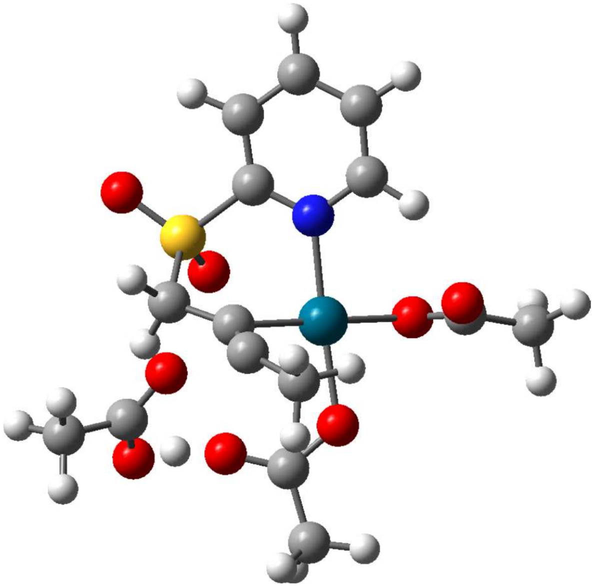

45

TS-β.xyz E=-1766.07522643 au

| | | | |
|---|---|---|---|
| C | 1.245899 | -1.691689 | -0.483373 |
| C | 0.951488 | -0.493703 | 0.316059 |
| C | -1.580709 | -2.141128 | -0.078262 |
| H | 1.882930 | -1.439338 | -1.346899 |
| C | -2.319780 | -3.248380 | 0.308035 |
| H | -2.027921 | -4.232614 | -0.058424 |
| C | -2.907550 | -0.687422 | 1.134251 |
| H | -3.052820 | 0.349517 | 1.453205 |
| C | -3.707568 | -1.744405 | 1.557831 |
| H | -4.550517 | -1.538089 | 2.217502 |
| C | -3.415600 | -3.040466 | 1.143797 |
| H | -4.029795 | -3.882285 | 1.467373 |
| N | -1.856389 | -0.887474 | 0.313646 |
| H | 1.686532 | -2.521077 | 0.088600 |
| S | -0.246208 | -2.377276 | -1.302219 |
| O | -0.574941 | -1.528878 | -2.463607 |
| O | -0.062161 | -3.838690 | -1.436677 |
| Pd | -0.631493 | 0.698311 | -0.174985 |
| C | 1.542840 | 0.067734 | 1.312327 |
| C | 1.591615 | 1.155603 | 2.280082 |
| H | 1.748926 | 0.778776 | 3.299808 |
| H | 2.394207 | 1.874675 | 2.053762 |

| | | | |
|---|---|---|---|
| H | 0.632155 | 1.692642 | 2.244710 |
| O | 3.210944 | -0.786669 | 1.711041 |
| C | 4.171507 | -0.688721 | 0.919298 |
| O | 4.199421 | -0.051512 | -0.209906 |
| C | 5.460445 | -1.362675 | 1.253199 |
| H | 5.693201 | -2.105428 | 0.479508 |
| H | 6.274666 | -0.628018 | 1.237752 |
| H | 5.407428 | -1.847922 | 2.230522 |
| C | -2.664400 | 2.571458 | 0.260028 |
| O | -2.323505 | 2.366509 | 1.435757 |
| O | -2.241117 | 1.896700 | -0.763142 |
| C | -3.632507 | 3.674991 | -0.093935 |
| H | -3.058986 | 4.553988 | -0.420057 |
| H | -4.283319 | 3.389301 | -0.929012 |
| H | -4.229594 | 3.960857 | 0.779150 |
| O | 0.562388 | 2.339854 | -0.535230 |
| C | 1.735144 | 2.243866 | -1.035901 |
| O | 2.310836 | 1.181352 | -1.374180 |
| H | 3.350769 | 0.436641 | -0.539483 |
| C | 2.451273 | 3.556915 | -1.227398 |
| H | 3.431255 | 3.516240 | -0.732093 |
| H | 2.649239 | 3.700024 | -2.297878 |
| H | 1.876881 | 4.407370 | -0.847418 |

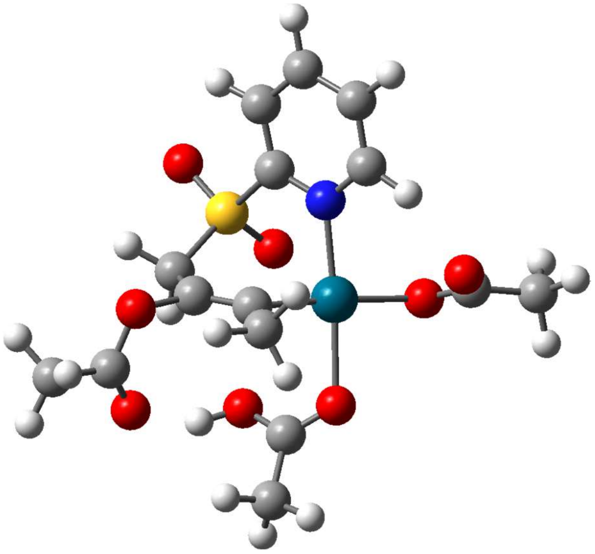

45
I5.xyz E=-1766.09261520 au

| | | | |
|---|---|---|---|
| C | 1.657136 | 1.529033 | 0.918614 |
| C | 1.762703 | 0.799275 | -0.374262 |
| C | -1.119120 | 2.157119 | 0.102002 |
| H | 2.058426 | 0.961381 | 1.774719 |
| C | -1.567061 | 3.417400 | -0.262386 |
| H | -1.145633 | 4.292299 | 0.232136 |
| C | -2.579196 | 1.095683 | -1.346328 |
| H | -2.943939 | 0.122301 | -1.697885 |
| C | -3.076619 | 2.324837 | -1.771713 |
| H | -3.869287 | 2.344096 | -2.519968 |

| | | | |
|---|---|---|---|
| C | -2.562334 | 3.501048 | -1.235496 |
| H | -2.938662 | 4.474014 | -1.556028 |
| N | -1.600853 | 1.019704 | -0.422408 |
| H | 2.196720 | 2.483780 | 0.858922 |
| S | 0.007077 | 2.025516 | 1.540896 |
| O | -0.526824 | 0.958702 | 2.410149 |
| O | 0.156757 | 3.412022 | 2.039656 |
| Pd | -0.701111 | -0.782366 | -0.023782 |
| C | 0.934897 | -0.079292 | -0.944997 |
| C | 1.181779 | -0.693065 | -2.283196 |
| H | 0.322344 | -0.518774 | -2.949916 |
| H | 2.080116 | -0.302407 | -2.787334 |
| H | 1.271146 | -1.788756 | -2.211947 |
| C | -3.216349 | -2.078282 | -0.063861 |
| O | -3.083168 | -1.864284 | -1.282446 |
| O | -2.398400 | -1.677209 | 0.852200 |
| C | -4.395613 | -2.864682 | 0.460671 |
| H | -4.044100 | -3.802785 | 0.911750 |
| H | -4.904597 | -2.308246 | 1.258697 |
| H | -5.102921 | -3.095001 | -0.343667 |
| O | 3.013428 | 1.146745 | -1.017507 |
| C | 4.083904 | 0.413632 | -0.666004 |
| O | 4.077556 | -0.476713 | 0.169677 |
| C | 5.289969 | 0.831504 | -1.441182 |
| H | 5.131879 | 0.629370 | -2.508729 |
| H | 5.446788 | 1.912860 | -1.347596 |
| H | 6.172998 | 0.290358 | -1.092210 |
| O | 0.423504 | -2.636019 | 0.222821 |
| C | 1.569813 | -2.567885 | 0.661169 |
| O | 1.918158 | -1.573751 | 1.468418 |
| H | 2.860411 | -1.341655 | 1.311542 |
| C | 2.610071 | -3.579826 | 0.321107 |
| H | 3.442081 | -3.078002 | -0.194573 |
| H | 3.023039 | -4.028487 | 1.233879 |
| H | 2.195037 | -4.358626 | -0.323874 |

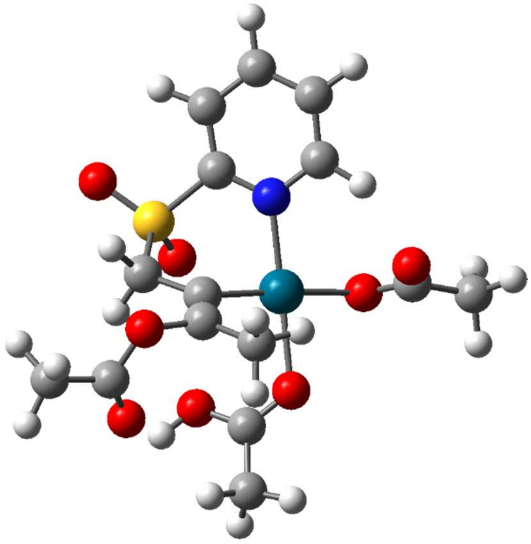

45

I6.xyz E=-1766.10826103 au

| | | | |
|---|---|---|---|
| C | 1.217483 | -1.712281 | -0.325658 |
| C | 0.943772 | -0.449038 | 0.417424 |
| C | -1.608020 | -2.087718 | -0.096520 |
| H | 1.955807 | -1.579847 | -1.135616 |
| C | -2.393716 | -3.176974 | 0.246253 |
| H | -2.123624 | -4.162282 | -0.133746 |
| C | -2.919351 | -0.613469 | 1.114276 |
| H | -3.039899 | 0.427867 | 1.436732 |
| C | -3.761135 | -1.653802 | 1.495849 |
| H | -4.612843 | -1.433590 | 2.139669 |
| C | -3.501805 | -2.949880 | 1.060421 |
| H | -4.150671 | -3.777563 | 1.351835 |
| N | -1.853369 | -0.836252 | 0.318185 |
| H | 1.536650 | -2.554666 | 0.307798 |
| S | -0.219683 | -2.337197 | -1.253453 |
| O | -0.454733 | -1.461708 | -2.420405 |
| O | -0.080579 | -3.802334 | -1.414213 |
| Pd | -0.597950 | 0.708775 | -0.147469 |
| C | 1.771786 | -0.043694 | 1.387255 |
| C | 1.660870 | 1.131967 | 2.283946 |
| H | 1.736860 | 0.823076 | 3.337834 |
| H | 2.473779 | 1.857580 | 2.110814 |
| H | 0.700040 | 1.643831 | 2.141512 |
| O | 2.944568 | -0.828724 | 1.673400 |
| C | 4.013742 | -0.631546 | 0.874851 |
| O | 4.054068 | 0.178578 | -0.032538 |
| C | 5.124875 | -1.560331 | 1.244811 |
| H | 4.874490 | -2.575510 | 0.906491 |
| H | 6.056166 | -1.248619 | 0.764162 |
| H | 5.249888 | -1.610932 | 2.332303 |
| C | -2.677982 | 2.625433 | 0.183996 |
| O | -2.455851 | 2.385713 | 1.383766 |
| O | -2.148607 | 1.985642 | -0.806852 |
| C | -3.626840 | 3.727696 | -0.227622 |
| H | -3.066375 | 4.527458 | -0.730919 |
| H | -4.358651 | 3.355499 | -0.956511 |
| H | -4.144169 | 4.146557 | 0.642530 |
| O | 0.788399 | 2.313377 | -0.481596 |
| C | 1.864612 | 2.138463 | -1.046443 |
| O | 2.042903 | 1.066095 | -1.813131 |
| H | 2.995554 | 0.928357 | -1.947848 |
| C | 2.985463 | 3.112668 | -0.934830 |
| H | 3.853989 | 2.608414 | -0.489012 |
| H | 3.282054 | 3.470358 | -1.929957 |
| H | 2.691298 | 3.961181 | -0.312027 |

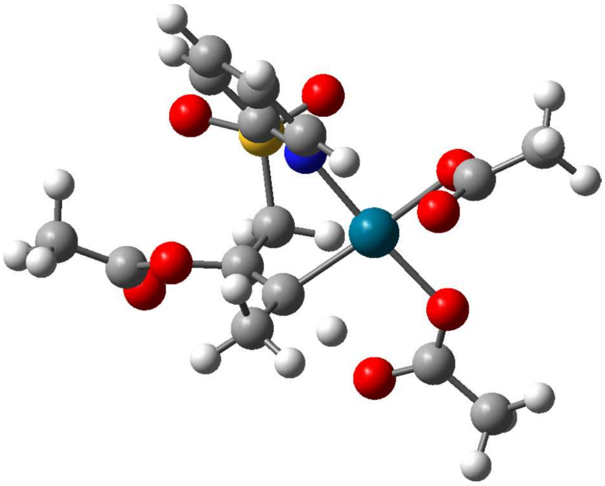

45
TS-Hα.xyz E=-1766.07965312 au

| | | | |
|---|---|---|---|
| C | 1.547433 | -1.101219 | 1.140640 |
| C | 1.495310 | -1.192553 | -0.337531 |
| C | 1.230802 | 1.696097 | 0.303118 |
| H | 0.579113 | -1.366020 | 1.582548 |
| C | 2.030497 | 2.780700 | -0.037390 |
| H | 3.021652 | 2.863502 | 0.407790 |
| C | -0.485496 | 2.418855 | -1.068036 |
| H | -1.495236 | 2.206496 | -1.440496 |
| C | 0.249467 | 3.535724 | -1.447257 |
| H | -0.189712 | 4.244498 | -2.149003 |
| C | 1.527465 | 3.721209 | -0.930646 |
| H | 2.129456 | 4.585258 | -1.216518 |
| N | 0.001011 | 1.503287 | -0.198665 |
| H | 2.366949 | -1.657065 | 1.612478 |
| S | 1.906533 | 0.625327 | 1.636733 |
| O | 1.177937 | 0.944978 | 2.878152 |
| O | 3.375879 | 0.803365 | 1.532595 |
| Pd | -1.234062 | -0.154819 | 0.021748 |
| C | 0.304717 | -1.256182 | -0.992844 |
| C | 0.230405 | -1.209845 | -2.486981 |
| H | 0.307353 | -0.172325 | -2.851896 |
| H | 1.043519 | -1.777595 | -2.964290 |
| H | -0.726306 | -1.606946 | -2.850935 |
| C | -3.535921 | 1.454133 | -0.029945 |
| O | -3.358428 | 1.354230 | -1.250602 |
| O | -2.703495 | 1.035203 | 0.877229 |
| C | -4.779940 | 2.085408 | 0.544783 |
| H | -5.447624 | 1.291189 | 0.905969 |
| H | -4.542073 | 2.718620 | 1.408101 |
| H | -5.306885 | 2.664248 | -0.221211 |
| O | 2.660631 | -0.963440 | -1.042659 |
| C | 3.870502 | -1.499766 | -0.608784 |
| O | 3.939787 | -2.399167 | 0.183698 |
| C | 4.989422 | -0.788625 | -1.282497 |
| H | 4.783920 | -0.648270 | -2.349803 |
| H | 5.082514 | 0.209120 | -0.831585 |

| | | | |
|---|---|---|---|
| H | 5.924179 | -1.336263 | -1.137332 |
| O | -2.612042 | -1.681867 | 0.201424 |
| C | -2.259988 | -2.896309 | 0.123961 |
| O | -1.083889 | -3.296087 | -0.134412 |
| H | -0.398075 | -2.222372 | -0.450147 |
| C | -3.311008 | -3.937458 | 0.361760 |
| H | -3.333252 | -4.635424 | -0.483723 |
| H | -3.042360 | -4.522586 | 1.250400 |
| H | -4.294899 | -3.483360 | 0.504656 |

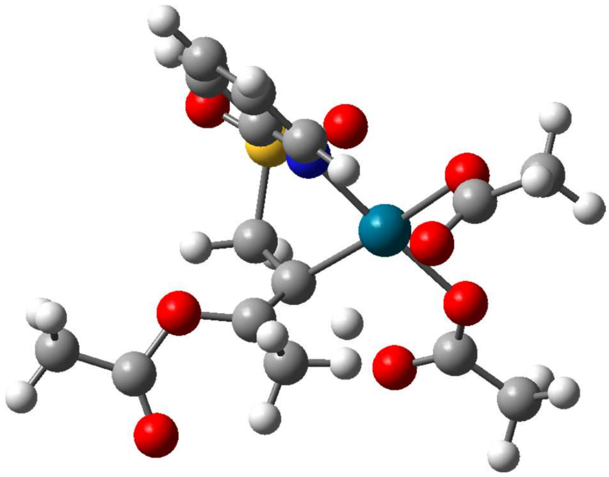

45

TS-Hβ.xyz E=-1766.08949849 au

| | | | |
|---|---|---|---|
| C | -1.632204 | 0.629847 | 1.595871 |
| C | -0.744929 | 1.066066 | 0.471553 |
| C | -1.128432 | -1.980876 | 0.577084 |
| H | -1.443740 | 1.199867 | 2.518656 |
| C | -1.980618 | -3.038065 | 0.297807 |
| H | -2.775271 | -3.274315 | 1.005543 |
| C | 0.081476 | -2.293666 | -1.367900 |
| H | 0.897235 | -1.922148 | -1.996447 |
| C | -0.726193 | -3.371019 | -1.718010 |
| H | -0.530166 | -3.897214 | -2.652465 |
| C | -1.766167 | -3.756048 | -0.877404 |
| H | -2.407350 | -4.601501 | -1.132146 |
| N | -0.113817 | -1.612554 | -0.220225 |
| H | -2.706162 | 0.653944 | 1.369387 |
| S | -1.319292 | -1.080617 | 2.150025 |
| O | -0.015684 | -1.130957 | 2.844131 |
| O | -2.549071 | -1.611945 | 2.777290 |
| Pd | 1.106575 | 0.001199 | 0.196013 |
| C | -1.292964 | 1.365617 | -0.743012 |
| C | -0.516536 | 1.801140 | -1.929371 |
| H | -0.565085 | 1.026761 | -2.711926 |
| H | -0.913628 | 2.729541 | -2.357530 |
| H | 0.541713 | 1.929609 | -1.672713 |
| O | -2.651430 | 1.136545 | -0.932457 |
| C | -3.467580 | 2.101759 | -1.501839 |
| O | -3.111832 | 3.228130 | -1.717545 |
| C | -4.817794 | 1.517672 | -1.747217 |

| | | | |
|---|---|---|---|
| H | -5.243259 | 1.138903 | -0.809227 |
| H | -5.478944 | 2.275955 | -2.174043 |
| H | -4.741944 | 0.661131 | -2.428940 |
| C | 3.168162 | -1.060776 | -1.378965 |
| O | 2.483135 | -0.565070 | -2.284232 |
| O | 2.805626 | -1.144462 | -0.133622 |
| C | 4.539591 | -1.631651 | -1.646117 |
| H | 5.293104 | -0.876025 | -1.384085 |
| H | 4.738204 | -2.512239 | -1.024019 |
| H | 4.652818 | -1.876048 | -2.708019 |
| O | 2.434667 | 1.556199 | 0.498199 |
| C | 2.059236 | 2.676140 | 0.955912 |
| O | 0.855995 | 3.003792 | 1.190797 |
| H | 0.117893 | 2.002856 | 0.814667 |
| C | 3.125617 | 3.685390 | 1.255820 |
| H | 2.799569 | 4.685328 | 0.949265 |
| H | 3.284170 | 3.712868 | 2.342182 |
| H | 4.068551 | 3.418298 | 0.770651 |

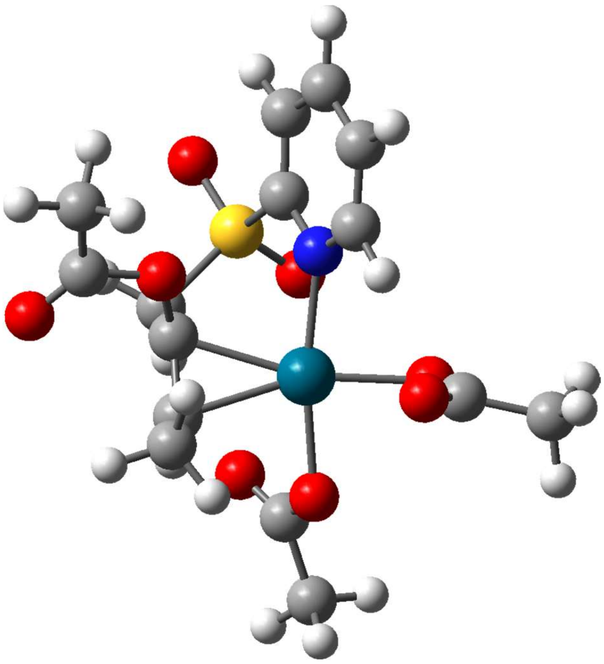

45

I7.xyz E=-1766.11337629 au

| | | | |
|---|---|---|---|
| C | 1.112807 | -1.761031 | 1.077197 |
| C | 0.819334 | -1.424408 | -0.337266 |
| C | 1.678550 | 1.099674 | 1.012921 |
| H | 0.250213 | -2.263309 | 1.538430 |
| C | 2.822934 | 1.866390 | 1.162469 |
| H | 3.541566 | 1.602469 | 1.938661 |
| C | 0.904599 | 2.377714 | -0.748086 |
| H | 0.102730 | 2.515318 | -1.479799 |
| C | 2.024918 | 3.199915 | -0.667846 |
| H | 2.125196 | 4.030961 | -1.366119 |
| C | 2.994040 | 2.947777 | 0.299151 |
| H | 3.878098 | 3.582513 | 0.380542 |

| | | | |
|---|---|---|---|
| N | 0.738196 | 1.342259 | 0.093503 |
| H | 2.040417 | -2.343696 | 1.176041 |
| S | 1.381902 | -0.284671 | 2.169970 |
| O | 0.115925 | 0.017916 | 2.863222 |
| O | 2.651207 | -0.472091 | 2.903985 |
| Pd | -0.912747 | 0.054138 | -0.150375 |
| C | -0.249778 | -1.895104 | -1.068541 |
| C | -0.364240 | -1.798696 | -2.546171 |
| H | 0.257774 | -0.994648 | -2.960469 |
| H | -0.049015 | -2.749234 | -3.005216 |
| H | -1.406654 | -1.617481 | -2.837615 |
| C | -2.447476 | 2.290750 | -0.774275 |
| O | -1.952235 | 2.083489 | -1.886490 |
| O | -2.122824 | 1.652391 | 0.316312 |
| C | -3.523573 | 3.323804 | -0.554569 |
| H | -4.498559 | 2.817182 | -0.555006 |
| H | -3.417946 | 3.819106 | 0.417604 |
| H | -3.516212 | 4.059557 | -1.365849 |
| O | 1.897946 | -0.817495 | -1.011258 |
| C | 2.730407 | -1.680521 | -1.700535 |
| O | 2.567659 | -2.871297 | -1.719395 |
| C | 3.809895 | -0.894348 | -2.363486 |
| H | 3.375925 | -0.110477 | -2.997349 |
| H | 4.420724 | -0.388233 | -1.604281 |
| H | 4.442425 | -1.555142 | -2.961416 |
| O | -2.696233 | -0.940137 | -0.286777 |
| C | -2.886242 | -1.844232 | 0.640099 |
| O | -2.014388 | -2.309140 | 1.375463 |
| H | -0.927239 | -2.559266 | -0.531733 |
| C | -4.327791 | -2.277707 | 0.733782 |
| H | -4.392551 | -3.280904 | 1.168442 |
| H | -4.856249 | -1.581145 | 1.398983 |
| H | -4.828845 | -2.243937 | -0.240174 |

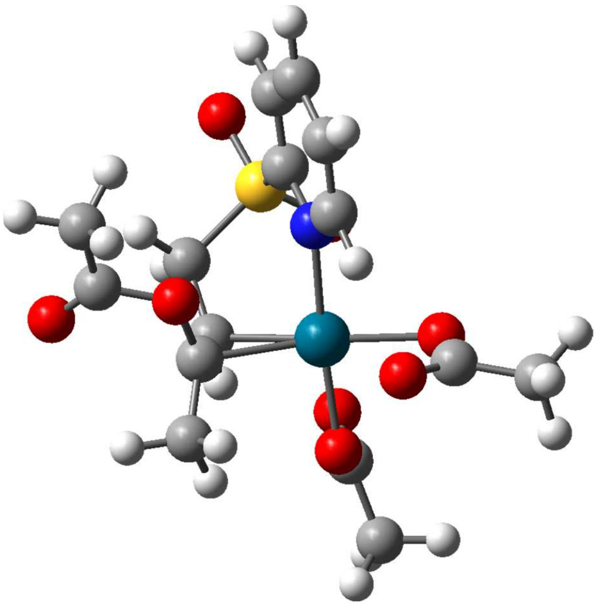

45

I8.xyz E=-1766.12608021 au

| | | | |
|---|---|---|---|
| C | 1.353549 | -1.843345 | 1.021164 |
| C | 0.168279 | -1.839576 | 0.114883 |
| C | 1.832500 | 0.950152 | 1.039606 |
| H | 1.309952 | -2.693327 | 1.716112 |
| C | 3.013248 | 1.670838 | 1.140443 |
| H | 3.753717 | 1.374725 | 1.883798 |
| C | 1.031919 | 2.268165 | -0.676984 |
| H | 0.207556 | 2.436663 | -1.377340 |
| C | 2.184612 | 3.047402 | -0.641865 |
| H | 2.283607 | 3.880134 | -1.338333 |
| C | 3.187063 | 2.752068 | 0.277693 |
| H | 4.096757 | 3.353200 | 0.323985 |
| N | 0.861060 | 1.232276 | 0.165912 |
| H | 2.319727 | -1.887955 | 0.499963 |
| S | 1.534781 | -0.438271 | 2.180337 |
| O | 0.262714 | -0.185919 | 2.875829 |
| O | 2.807871 | -0.643487 | 2.902166 |
| Pd | -0.874028 | 0.058422 | -0.056419 |
| C | 0.217547 | -1.426006 | -1.219876 |
| C | -0.652511 | -1.896202 | -2.328188 |
| H | -0.925066 | -1.061391 | -2.988136 |
| H | -0.093724 | -2.640419 | -2.916281 |
| H | -1.570794 | -2.347235 | -1.944163 |
| O | 1.395258 | -0.783972 | -1.665456 |
| C | 2.424113 | -1.606690 | -2.067152 |
| O | 2.365191 | -2.808014 | -2.007649 |
| C | 3.571402 | -0.776267 | -2.536032 |
| H | 3.948677 | -0.164005 | -1.705571 |
| H | 4.372055 | -1.419735 | -2.909565 |
| H | 3.245906 | -0.081514 | -3.320187 |
| C | -2.332253 | 2.322521 | -0.710879 |
| O | -1.824856 | 2.039233 | -1.806123 |
| O | -2.040834 | 1.726533 | 0.405580 |
| C | -3.369548 | 3.407010 | -0.572083 |
| H | -4.363857 | 2.940261 | -0.543869 |
| H | -3.248843 | 3.961511 | 0.365965 |
| H | -3.332207 | 4.087421 | -1.429494 |
| O | -2.633042 | -0.904621 | -0.375333 |
| C | -3.094112 | -1.540995 | 0.680587 |
| O | -2.456612 | -1.776118 | 1.699772 |
| H | -0.671851 | -2.465444 | 0.418574 |
| C | -4.536083 | -1.952691 | 0.506120 |
| H | -4.774902 | -2.779827 | 1.182844 |
| H | -5.175365 | -1.097920 | 0.766585 |
| H | -4.763840 | -2.223556 | -0.531268 |

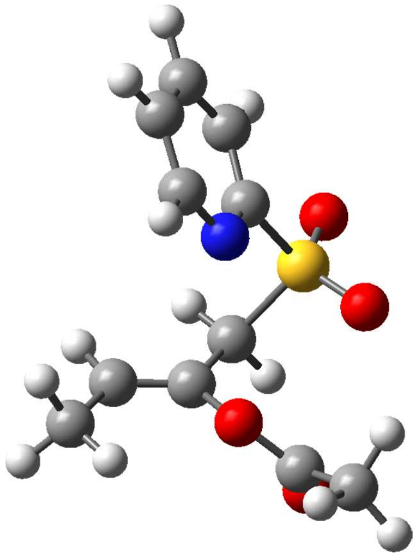

30
I9.xyz E=-1180.97311618 au

| | | | |
|---|---|---|---|
| C | 0.741944 | -0.381702 | -1.455190 |
| C | 1.021524 | 0.915029 | -0.783272 |
| C | -1.575107 | -0.578026 | 0.118375 |
| H | 0.072204 | -0.247070 | -2.312775 |
| C | -1.795399 | -0.319500 | 1.465503 |
| H | -1.104507 | -0.705445 | 2.215357 |
| C | -3.392037 | 0.585154 | -0.564241 |
| H | -4.007178 | 0.937634 | -1.397498 |
| C | -3.729298 | 0.904660 | 0.752216 |
| H | -4.613703 | 1.508745 | 0.959630 |
| C | -2.917144 | 0.443585 | 1.784893 |
| H | -3.149227 | 0.678794 | 2.825347 |
| N | -2.325832 | -0.152226 | -0.893455 |
| H | 1.640887 | -0.928570 | -1.766720 |
| S | -0.125661 | -1.565752 | -0.369941 |
| O | -0.560967 | -2.701904 | -1.204651 |
| O | 0.674235 | -1.783852 | 0.861879 |
| C | 0.471166 | 2.077068 | -1.162995 |
| C | 0.667380 | 3.388728 | -0.496957 |
| H | 1.368485 | 3.318429 | 0.342160 |
| H | 1.046785 | 4.144281 | -1.201438 |
| H | -0.286330 | 3.782831 | -0.111101 |
| O | 1.754841 | 0.883387 | 0.400230 |
| C | 2.918244 | 0.146219 | 0.446762 |
| O | 3.473994 | -0.275831 | -0.534906 |
| C | 3.339414 | -0.031617 | 1.863718 |
| H | 3.189862 | 0.885063 | 2.445310 |
| H | 2.702345 | -0.810943 | 2.304298 |
| H | 4.383257 | -0.353583 | 1.908572 |
| H | -0.192428 | 2.035452 | -2.031183 |

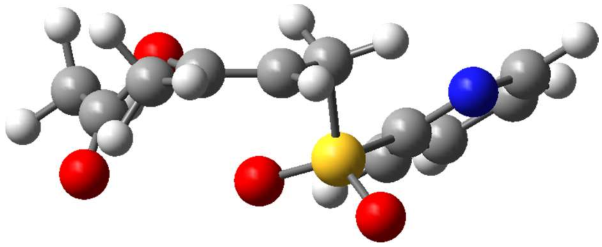

30
I10.xyz E=-1180.97352776 au

| | | | |
|---|---|---|---|
| C | 0.214013 | -1.216381 | -0.770263 |
| C | 1.558046 | -1.759786 | -0.457646 |
| C | -2.192420 | -0.031951 | 0.144741 |
| H | 0.235664 | -0.432489 | -1.539141 |
| C | -2.702829 | 1.243532 | 0.364203 |
| H | -2.078175 | 2.009404 | 0.824819 |
| C | -4.102663 | -0.796449 | -0.803909 |
| H | -4.631699 | -1.628489 | -1.277704 |
| C | -4.733044 | 0.435007 | -0.622322 |
| H | -5.765172 | 0.574697 | -0.947020 |
| C | -4.019972 | 1.472873 | -0.026645 |
| H | -4.480929 | 2.450709 | 0.125930 |
| N | -2.844184 | -1.043392 | -0.423237 |
| H | -0.517091 | -1.988043 | -1.041969 |
| S | -0.501542 | -0.409724 | 0.704871 |
| O | -0.579391 | -1.408260 | 1.790743 |
| O | 0.166713 | 0.899825 | 0.918301 |
| C | 2.679429 | -1.024903 | -0.509752 |
| C | 4.052867 | -1.516410 | -0.246222 |
| H | 4.714738 | -1.277088 | -1.091480 |
| H | 4.477055 | -1.031486 | 0.642198 |
| H | 4.051794 | -2.602271 | -0.097803 |
| O | 2.544561 | 0.291090 | -0.952130 |
| C | 2.962050 | 1.312305 | -0.122661 |
| O | 3.545192 | 1.136514 | 0.913554 |
| C | 2.550584 | 2.618392 | -0.711780 |
| H | 1.467945 | 2.728897 | -0.563759 |
| H | 3.066502 | 3.437391 | -0.203541 |
| H | 2.743546 | 2.648766 | -1.790452 |
| H | 1.636812 | -2.801388 | -0.145563 |

5.6. Thermochemical values

| Molecule | $E_{elec}$ (au) | $G_{corr}$ (au) | G (au) | ΔG (kcal $mol^{-1}$) |
|---|---|---|---|---|
| $Pd_3OAc_6$ | -1755.458243 | 0.225556 | -1755.232687 | -13.6[a] |
| **I1** | -951.793882 | 0.112138 | -951.681744 | 0.0[b] |
| $PdOAc_2$ | -585.111203 | 0.055236 | -585.055967 | |
| **I2** | -1536.940622 | 0.198586 | -1536.742036 | -2.7[c] |
| AcOH | -229.141290 | 0.028617 | -229.112673 | |
| **I3** | -1766.101072 | 0.249943 | -1765.851129 | -0.5 |
| **I4** | -1766.099880 | 0.248145 | -1765.851735 | -0.8 |
| TS-α | -1766.062618 | 0.247328 | -1765.815290 | 22.0 |
| TS-β | -1766.075226 | 0.248174 | -1765.827052 | 14.6 |
| **I5** | -1766.092615 | 0.255041 | -1765.837574 | 8.0 |
| **I6** | -1766.108261 | 0.255282 | -1765.852979 | -1.6 |
| TS-Hα | -1766.079653 | 0.249085 | -1765.830568 | 12.4 |
| TS-Hβ | -1766.089498 | 0.246379 | -1765.843119 | 4.6 |
| **I7** | -1766.113376 | 0.255773 | -1765.857603 | -4.5 |
| **I8** | -1766.126080 | 0.256619 | -1765.869461 | -12.0 |
| **I9** | -1180.973116 | 0.171785 | -1180.801331 | -4.3[d] |
| **I10** | -1180.973528 | 0.170688 | -1180.802840 | -5.3[e] |

$E_{elec}$: Electronic energy. $G_{corr}$: Thermal correction to Gibbs free energy. [a]**I1** + 1/3 $Pd_3OAc_6$ + AcOH. [b]**I1** + $PdOAc_2$ + AcOH. [c]**I2** + AcOH. [d]**I9** + $PdOAc_2$. [e]**I10** + $PdOAc_2$.

# 6 Unsuccessful substrates

The following substrates led to decomposition of starting material or degradation of the pre-catalyst source to form catalytically inactive Pd black in the media:

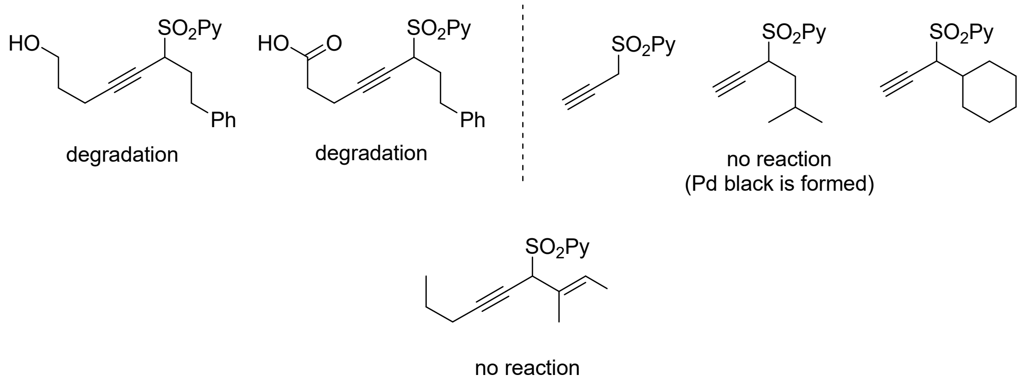

# 7 References


[1] García-Rubia, A.; Romero-Revilla, J. A.; Mauleón, P.; Arrayás, R. G.; Carretero, J. C. Cu-Catalyzed Silylation of Alkynes: A Traceless 2-Pyridylsulfonyl Controller Allows Access to Either Regioisomer on Demand. *J. Am. Chem Soc.* **2015**, *137*, 6857-6865.

[2] Corpas, J; Quirós, M. T.; Mauleón, P.; Arrayás, R. G.; Carretero, J. C. Metal- and Photocatalysis to Gain Regiocontrol and Stereodivergence in Hydroarylations of Unsymmetrical Dialkyl Alkynes. *ACS Catal.* **2019**, *9*, 10567-10574.

[3] Gaussian 09, Revision E.01, Frisch, M. J.; Trucks, G. W.; Schlegel, H. B.; Scuseria, G. E.; Robb, M. A.; Cheeseman, J. R.; Scalmani, G.; Barone, V.; Mennucci, B.; Petersson, G. A.; Nakatsuji, H.; Caricato, M.; Li, X.; Hratchian, H. P.; Izmaylov, A. F.; Bloino, J.; Zheng, G.; Sonnenberg, J. L.; Hada, M.; Ehara, M.; Toyota, K.; Fukuda, R.; Hasegawa, J.; Ishida, M.; Nakajima, T.; Honda, Y.; Kitao, O.; Nakai, H.; Vreven, T.; Montgomery, J. A., Jr.; Peralta, J. E.; Ogliaro, F.; Bearpark, M.; Heyd, J. J.; Brothers, E.; Kudin, K. N.; Staroverov, V. N.; Kobayashi, R.; Normand, J.; Raghavachari, K.; Rendell, A.; Burant, J. C.; Iyengar, S. S.; Tomasi, J.; Cossi, M.; Rega, N.; Millam, J. M.; Klene, M.; Knox, J. E.; Cross, J. B.; Bakken, V.; Adamo, C.; Jaramillo, J.; Gomperts, R.; Stratmann, R. E.; Yazyev, O.; Austin, A. J.; Cammi, R.; Pomelli, C.; Ochterski, J. W.; Martin, R. L.; Morokuma, K.; Zakrzewski, V. G.; Voth, G. A.; Salvador, P.; Dannenberg, J. J.; Dapprich, S.; Daniels, A. D.; Farkas, Ö.; Foresman, J. B.; Ortiz, J. V.; Cioslowski, J.; Fox, D. J. Gaussian, Inc., Wallingford CT, 2009.

[4] Zhao, Y.; Truhlar, D. G. The M06 Suite of Density Functionals for Main Group Thermochemistry, Thermochemical Kinetics, Noncovalent Interactions, Excited States, and Transition Elements: Two New Functionals and Systematic Testing of Four M06- Class Functionals and 12 Other Functionals. Theor. Chem. Acc. 2008, 120, 215-241.

[5] Dunning, T. H. Gaussian Basis Sets for Use in Correlated Molecular Calculations. I. The Atoms Boron through Neon and Hydrogen. *J. Chem. Phys.* **1989**, *90*, 1007-1023.

[6] Weigend, F.; Ahlrichs, R. Balanced Basis Sets of Split Valence, Triple Zeta Valence and Quadruple Zeta Valence Quality for H to Rn: Design and Assessment of Accuracy. *Phys. Chem. Chem. Phys.* **2005**, *7*, 3297-3305.

[7] Marenich, A. V.; Cramer, C. J.; Truhlar, D. G. Universal Solvation Model Based Onsolute Electron Density and on a Continuum Model of the Solvent Defined by the Bulk Dielectric Constant and Atomic Surface Tensions., *J. Phys. Chem. B* **2009**, *113*, 6378-6396.

[8] Ayers, P. W.; Morrison, R. C.; Roy, R. K. Variational Principles for Describing Chemical Reactions: Condensed Reactivity Indices. *J. Chem. Phys.* **2002**, *116*, 8731-8744.

# 8 NMR spectra

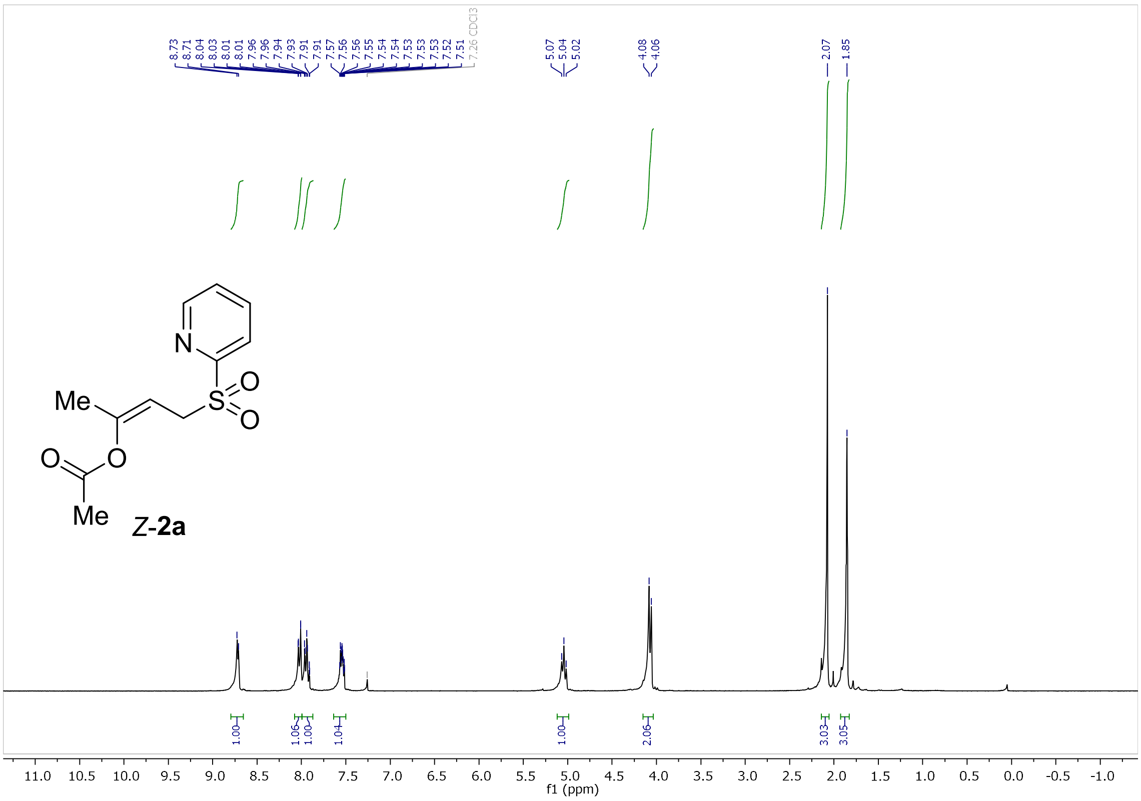


**Figure S7.** $^{1}$**H NMR spectrum of product** ***Z*****-2a.**

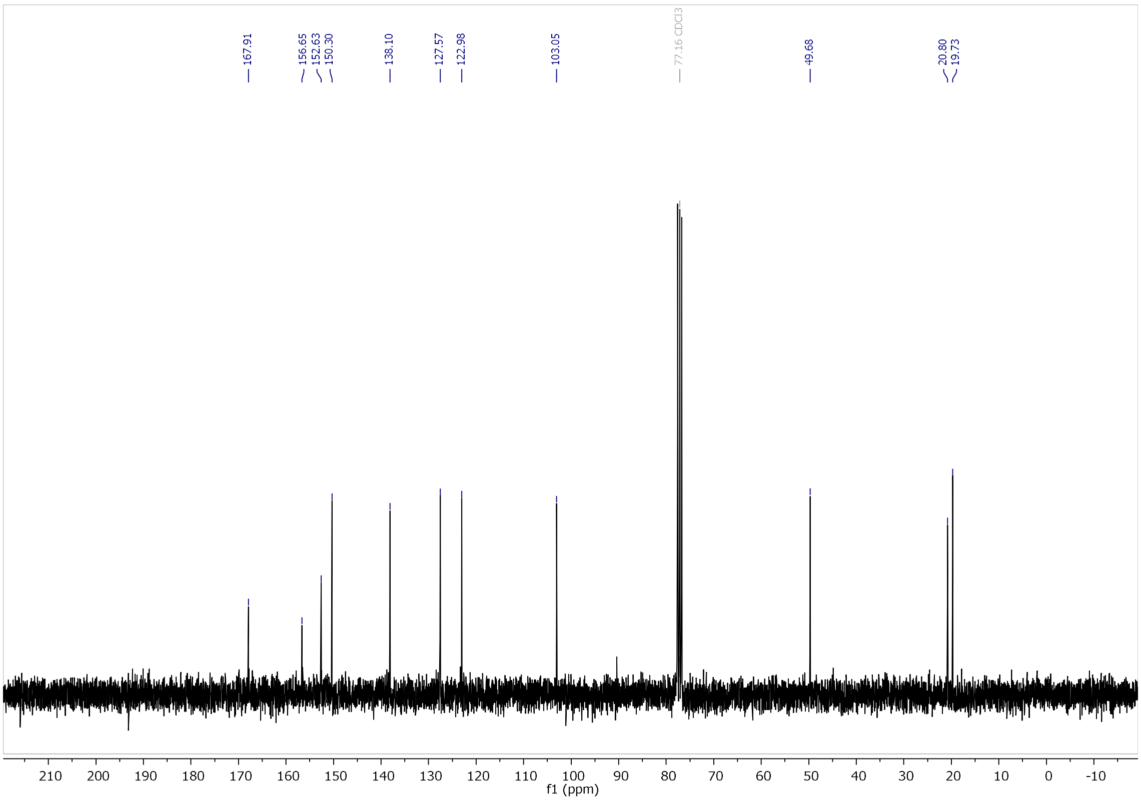


**Figure S8.** $^{13}$**C NMR spectrum of product** ***Z*****-2a.**

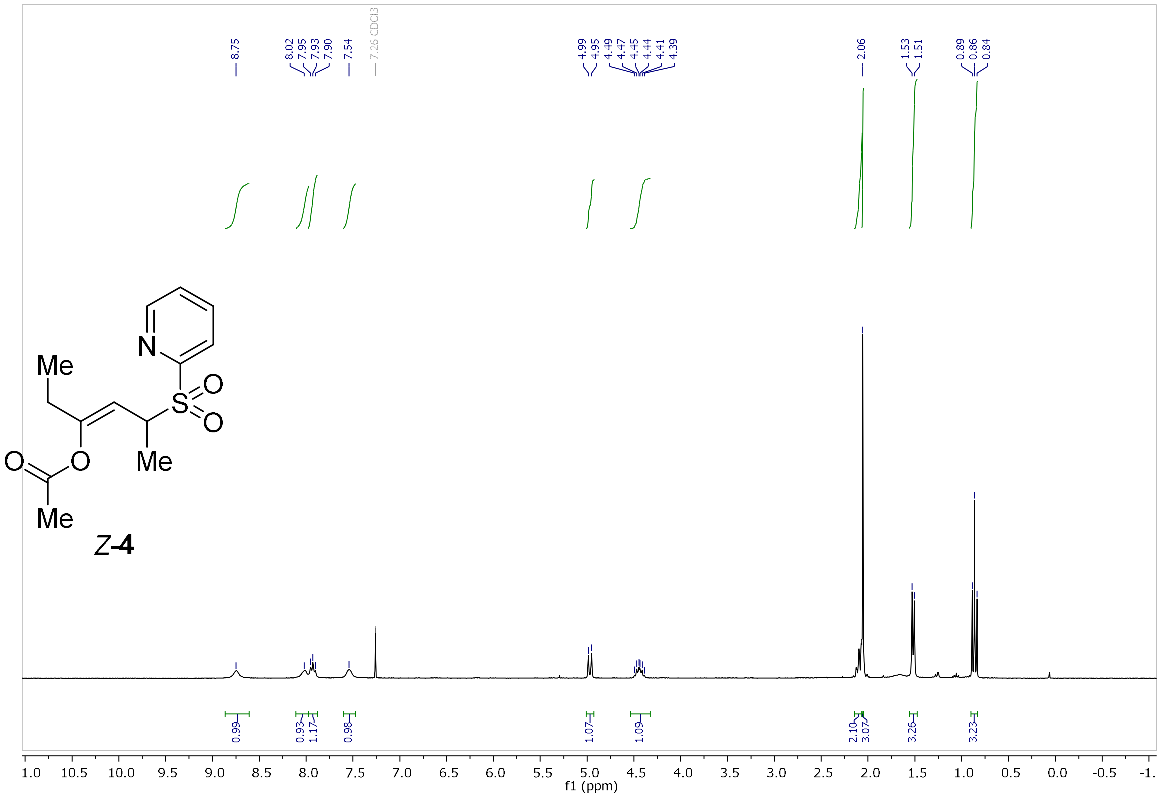

**Figure S9. $^{1}H$ NMR spectrum of product *Z*-4.**

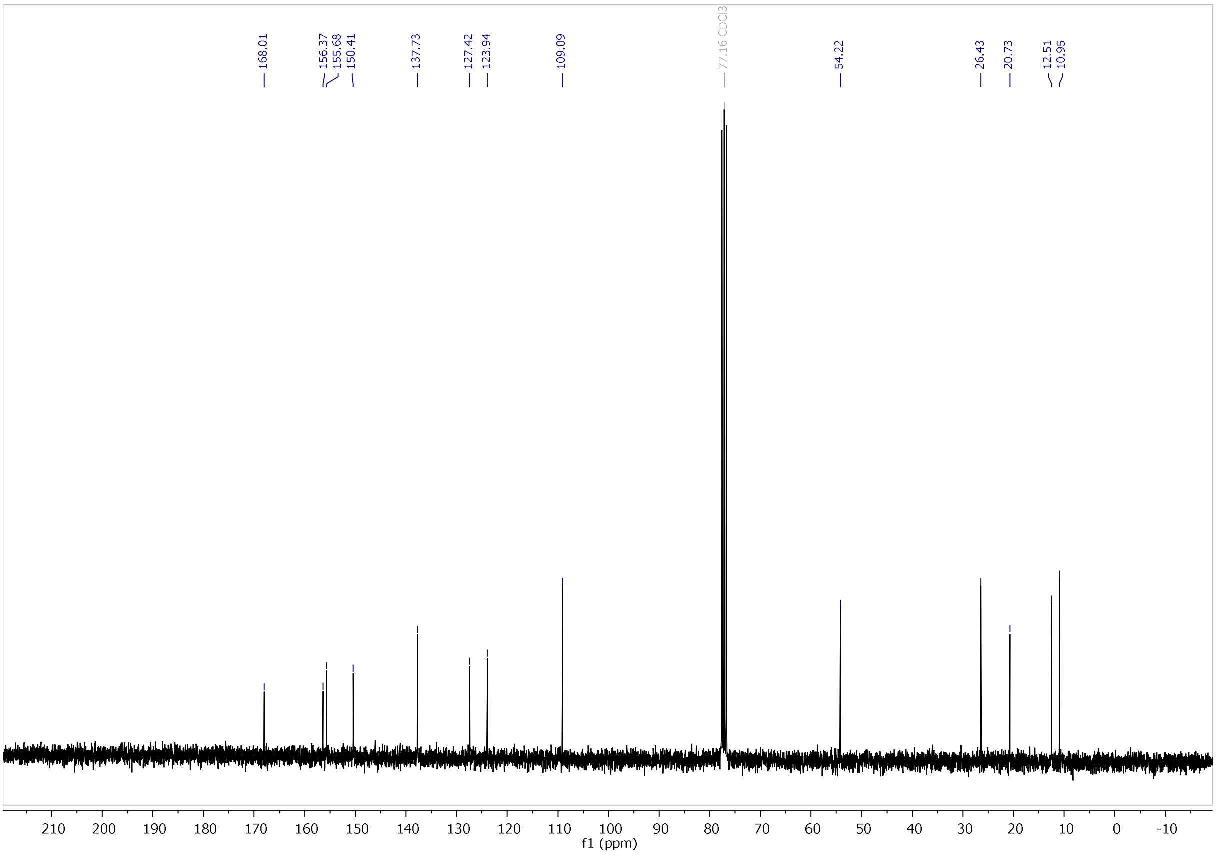

**Figure S10. $^{13}C$ NMR spectrum of product *Z*-4.**

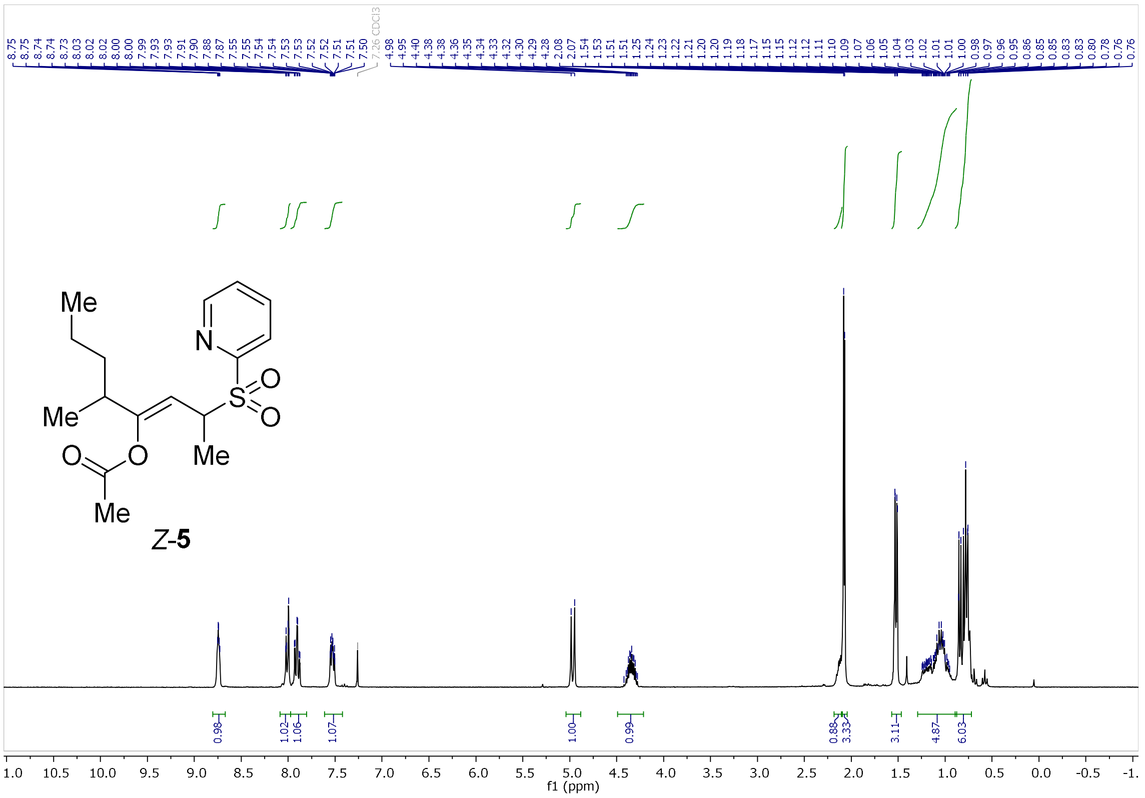


**Figure S11. $^{1}$H NMR spectrum of product *Z*-5.**

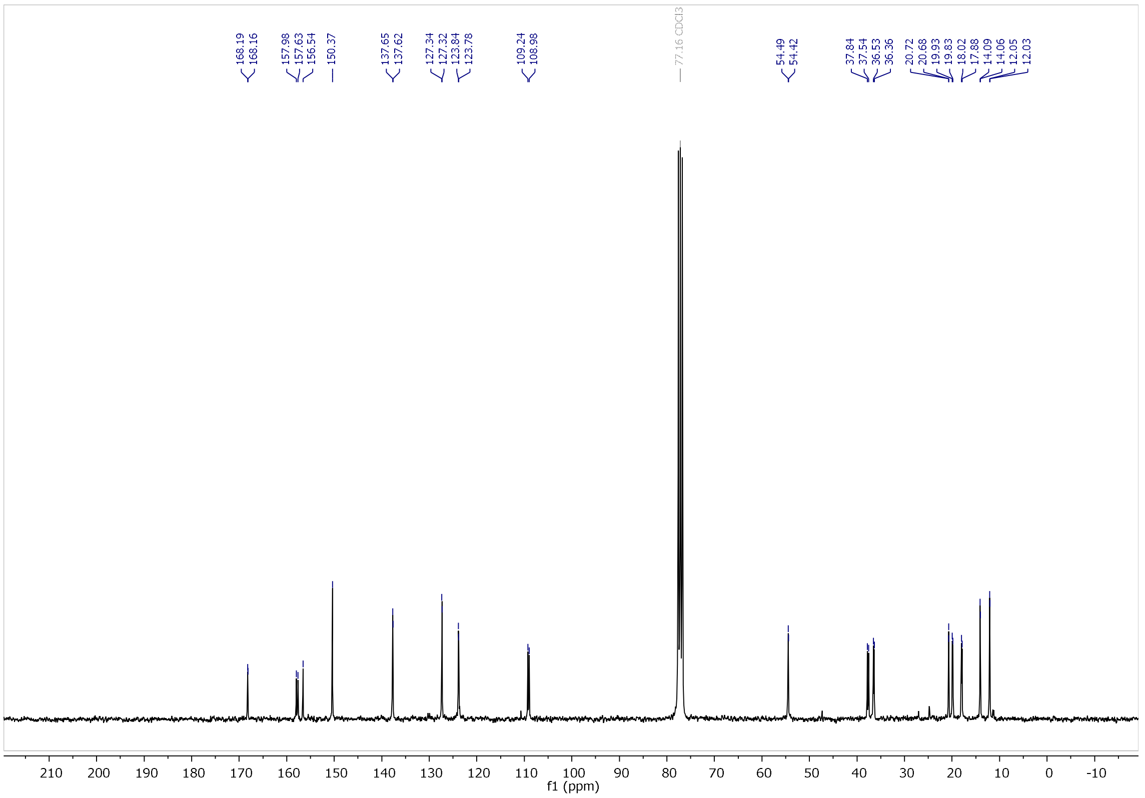


**Figure S12. $^{13}$C NMR spectrum of product *Z*-5.**

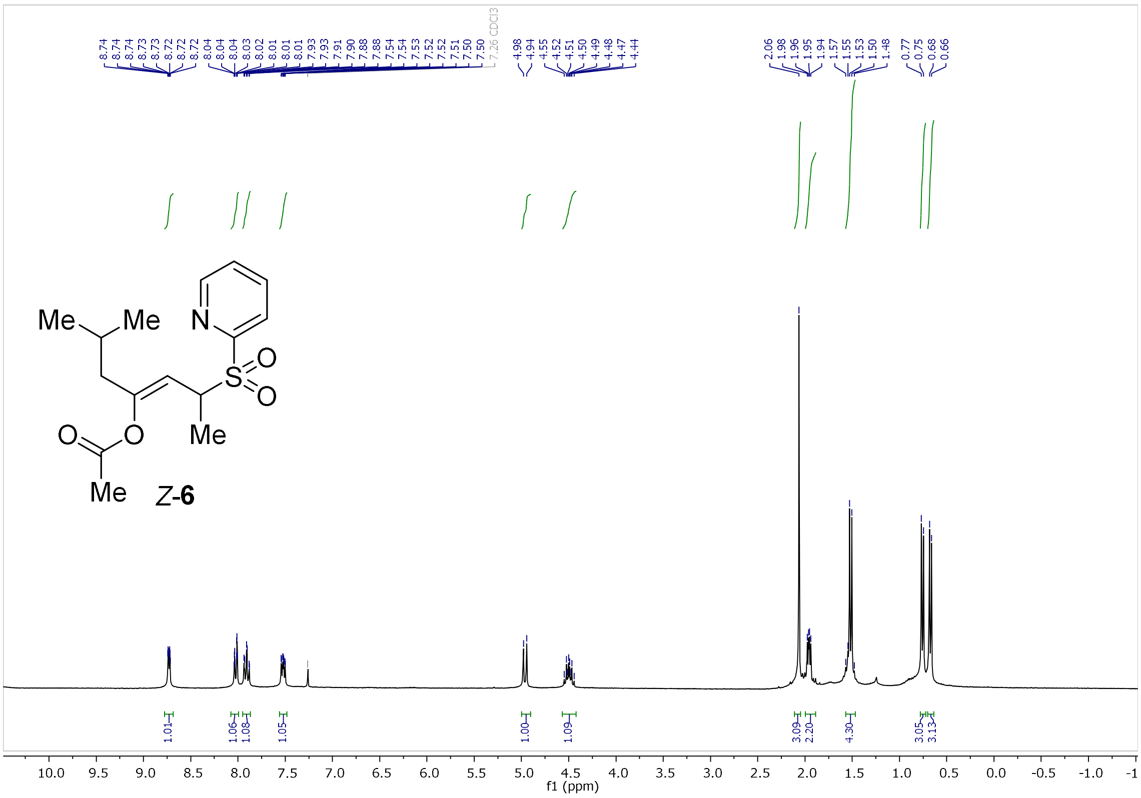


**Figure S13.** **$^{1}$H NMR spectrum of product *Z*-6.**

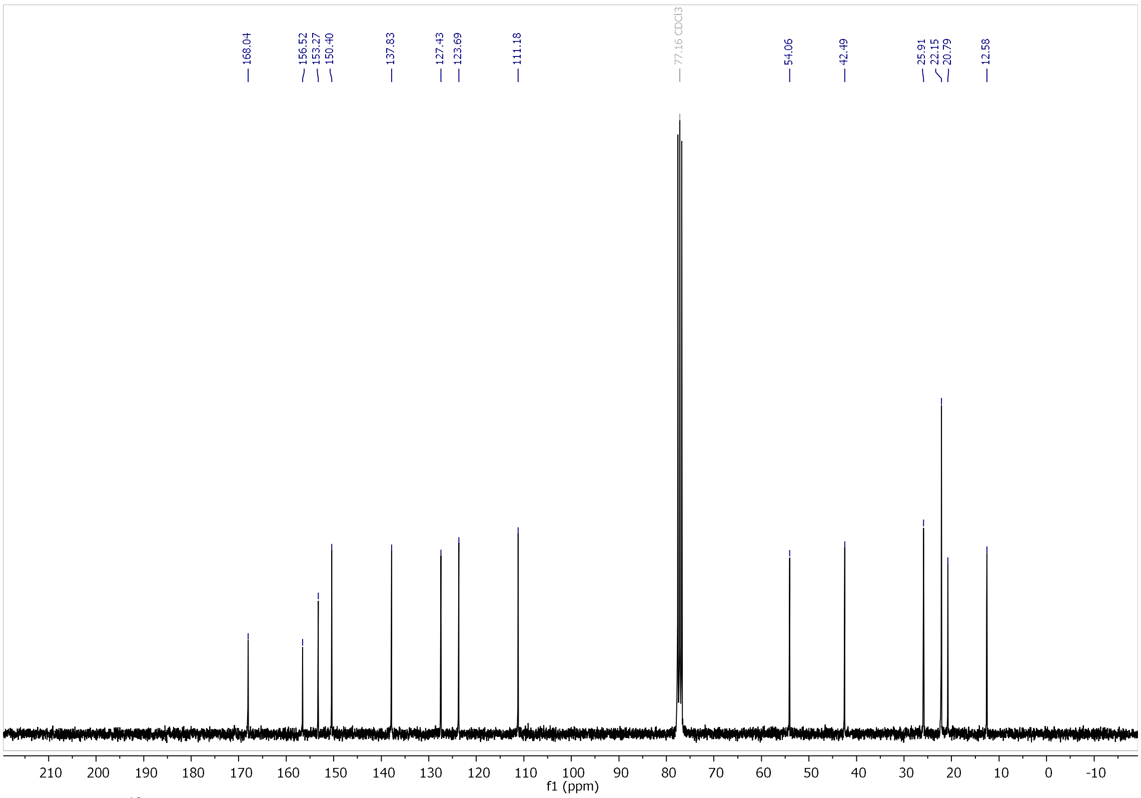


**Figure S14.** **$^{13}$C NMR spectrum of product *Z*-6.**

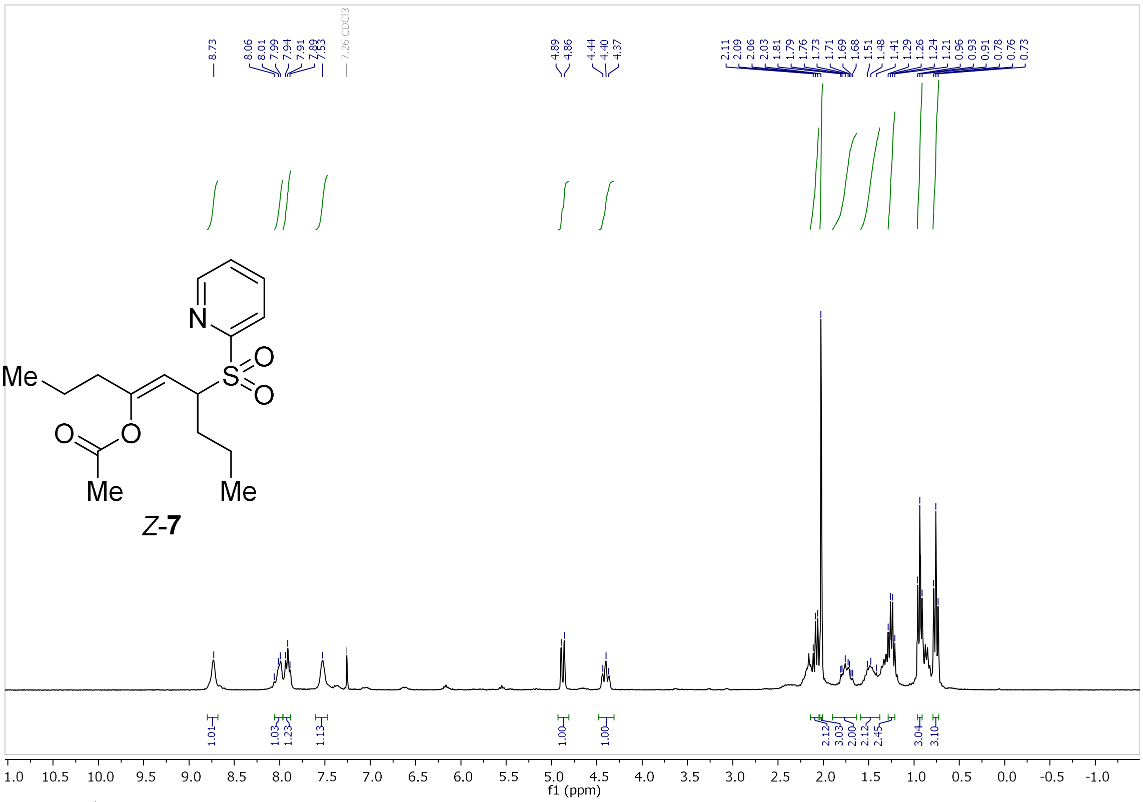


**Figure S15.** **$^{1}$H NMR spectrum of product *Z*-7.**

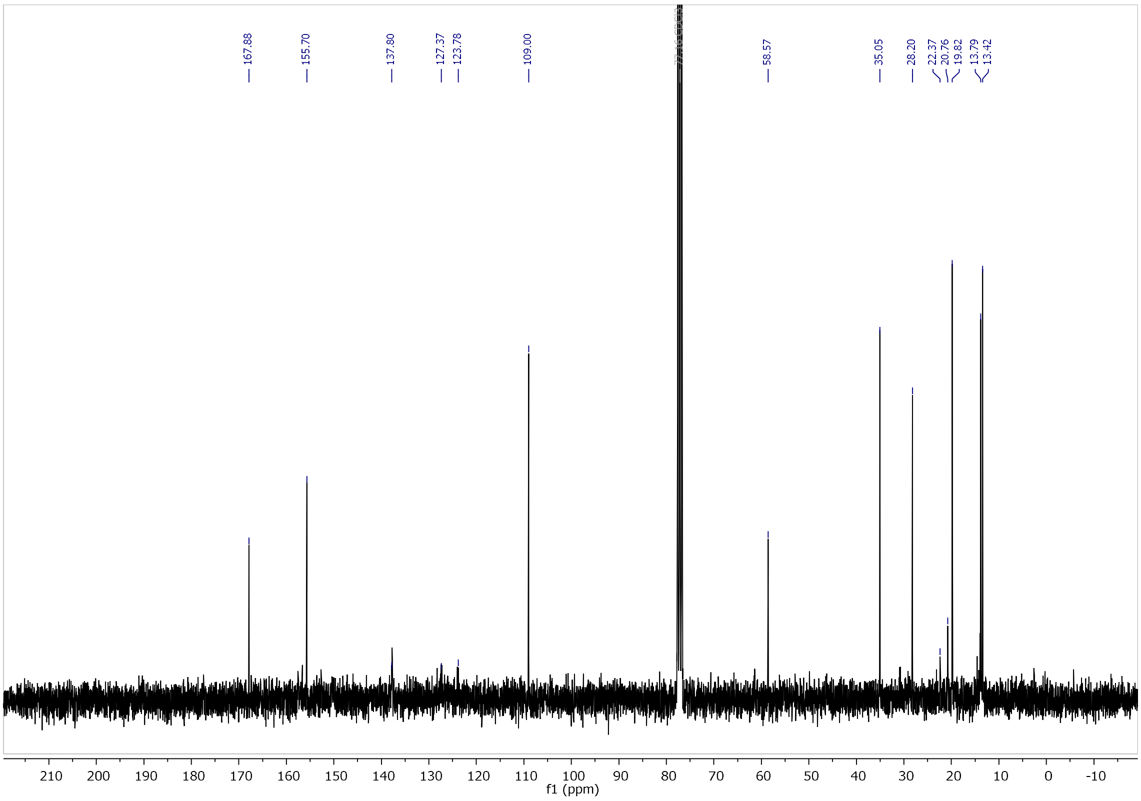


**Figure S16.** **$^{13}$C NMR spectrum of product *Z*-7.**

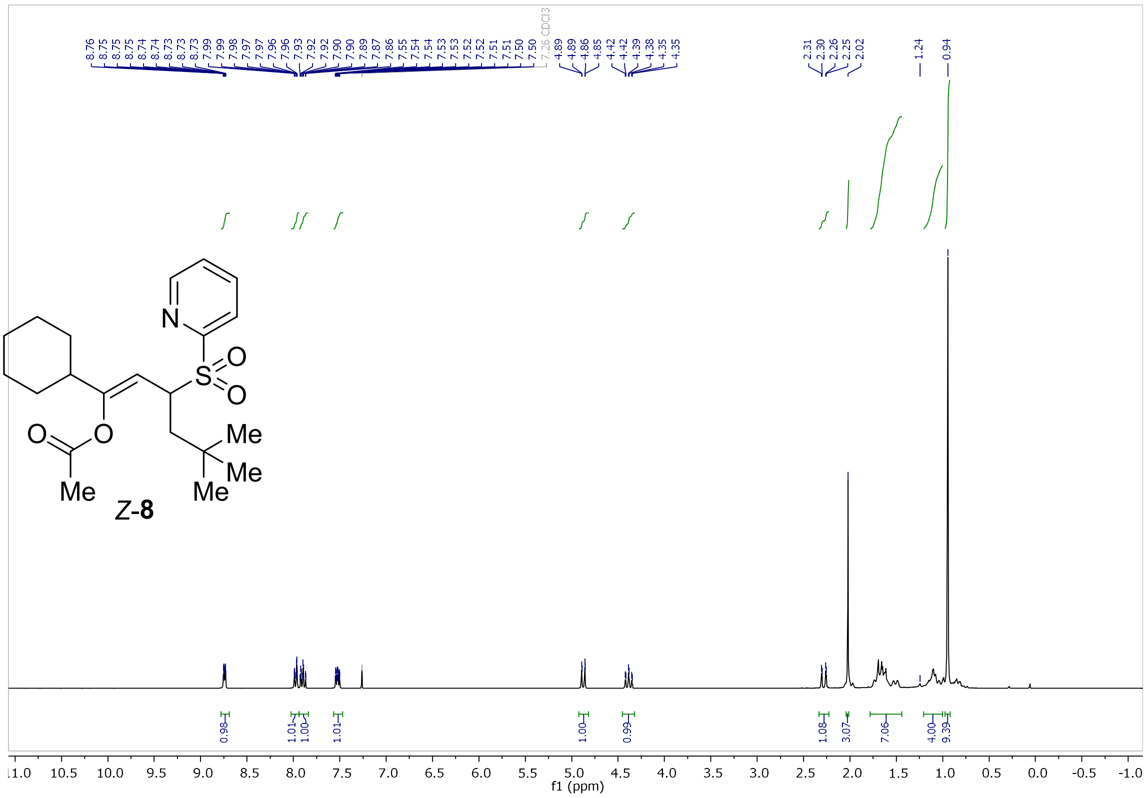


**Figure S17.** **$^{1}$H NMR spectrum of product *Z*-8.**

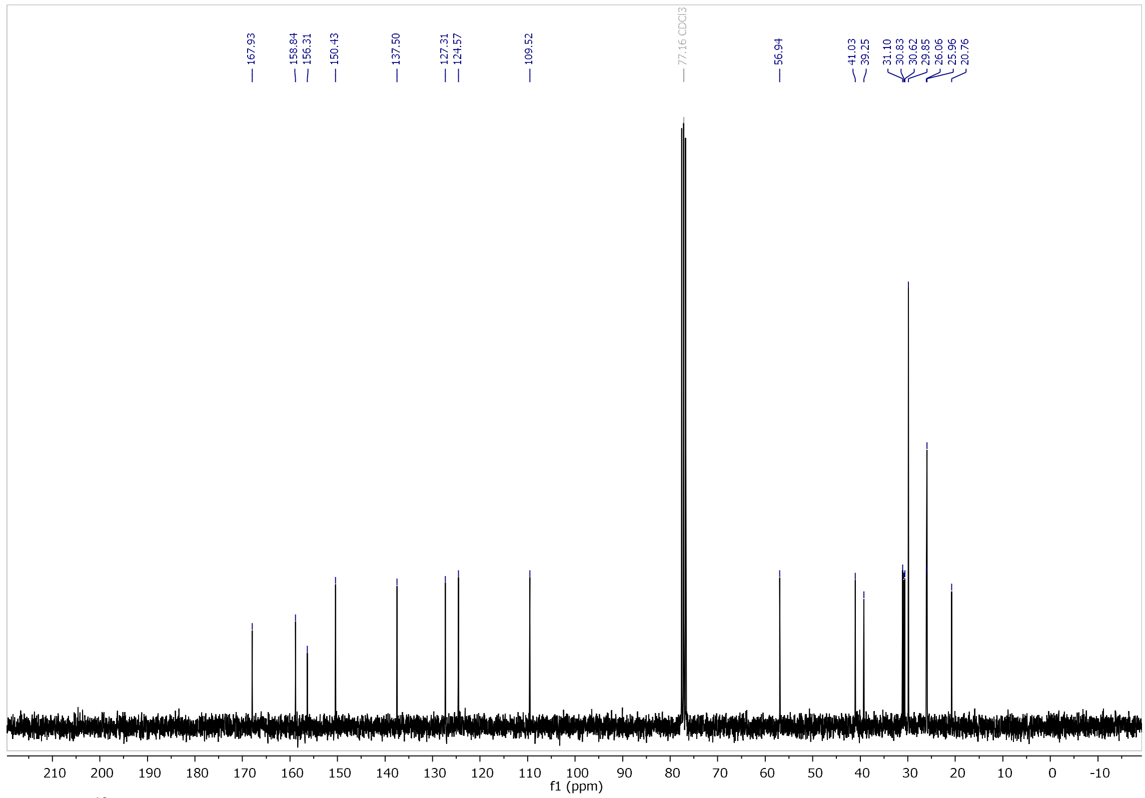


**Figure S18.** **$^{13}$C NMR spectrum of product *Z*-8.**

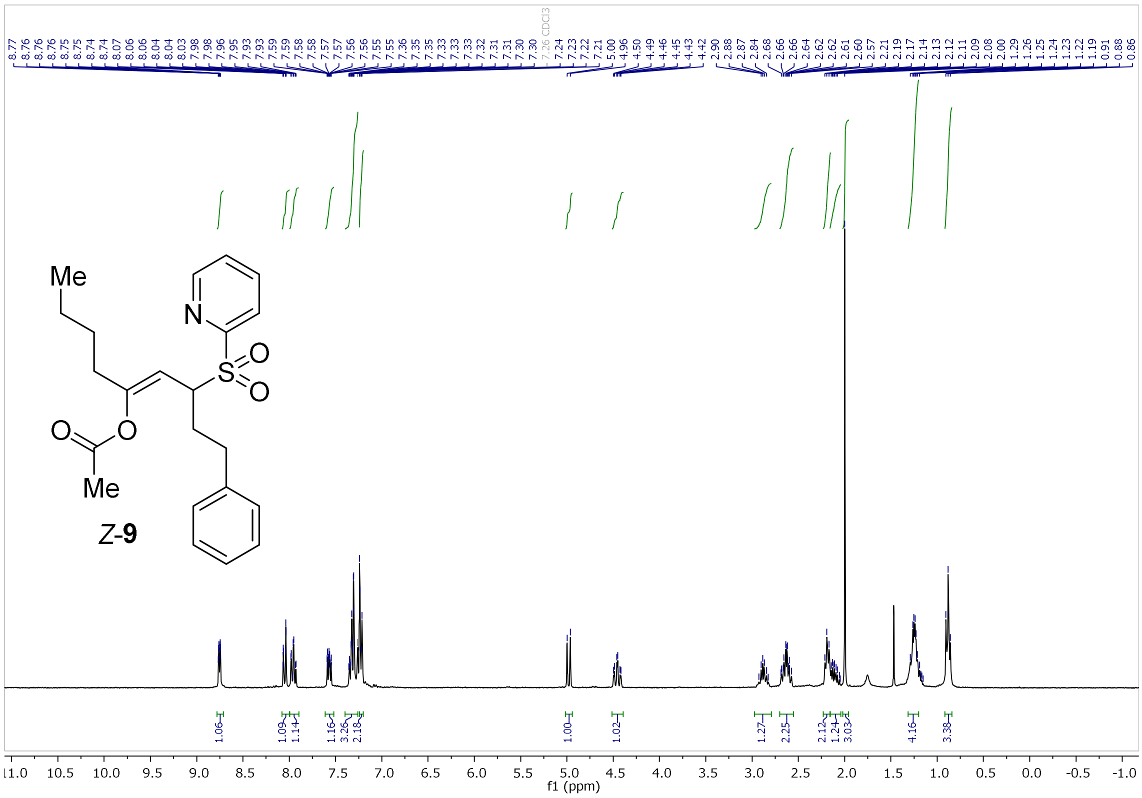

**Figure S19.** **$^{1}$H NMR spectrum of product *Z*-9.**

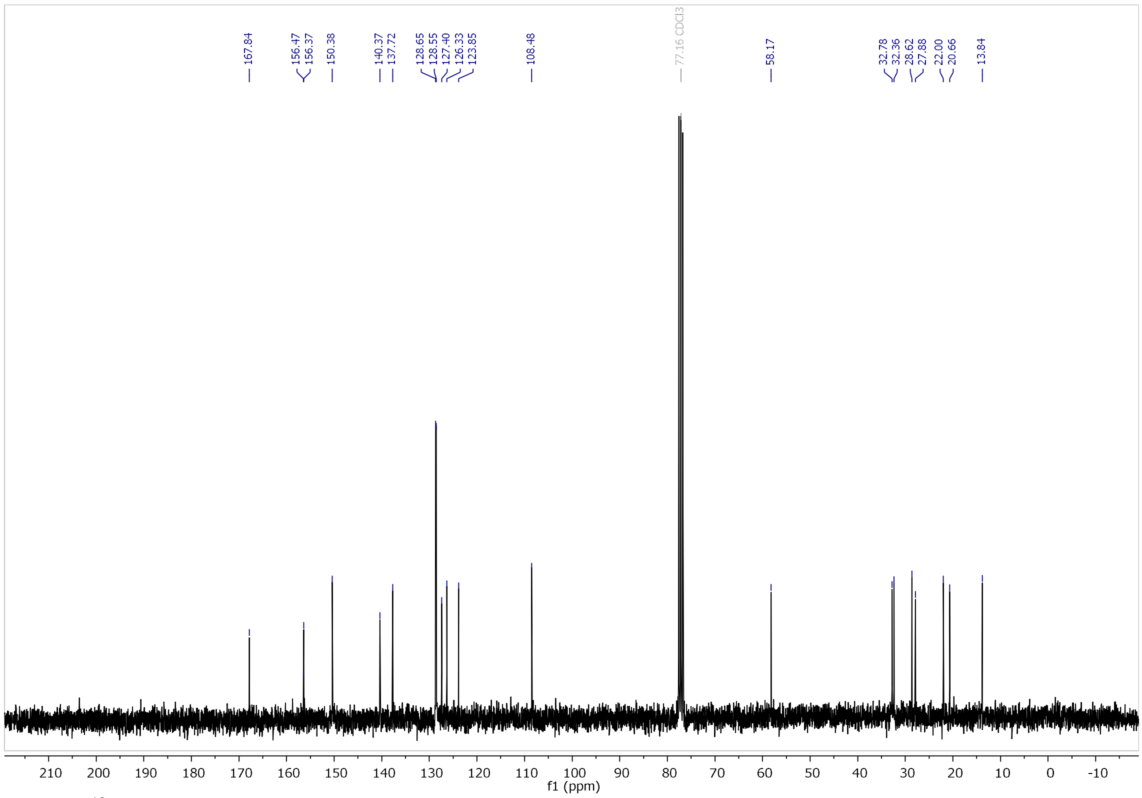

**Figure S20.** **$^{13}$C NMR spectrum of product *Z*-9.**

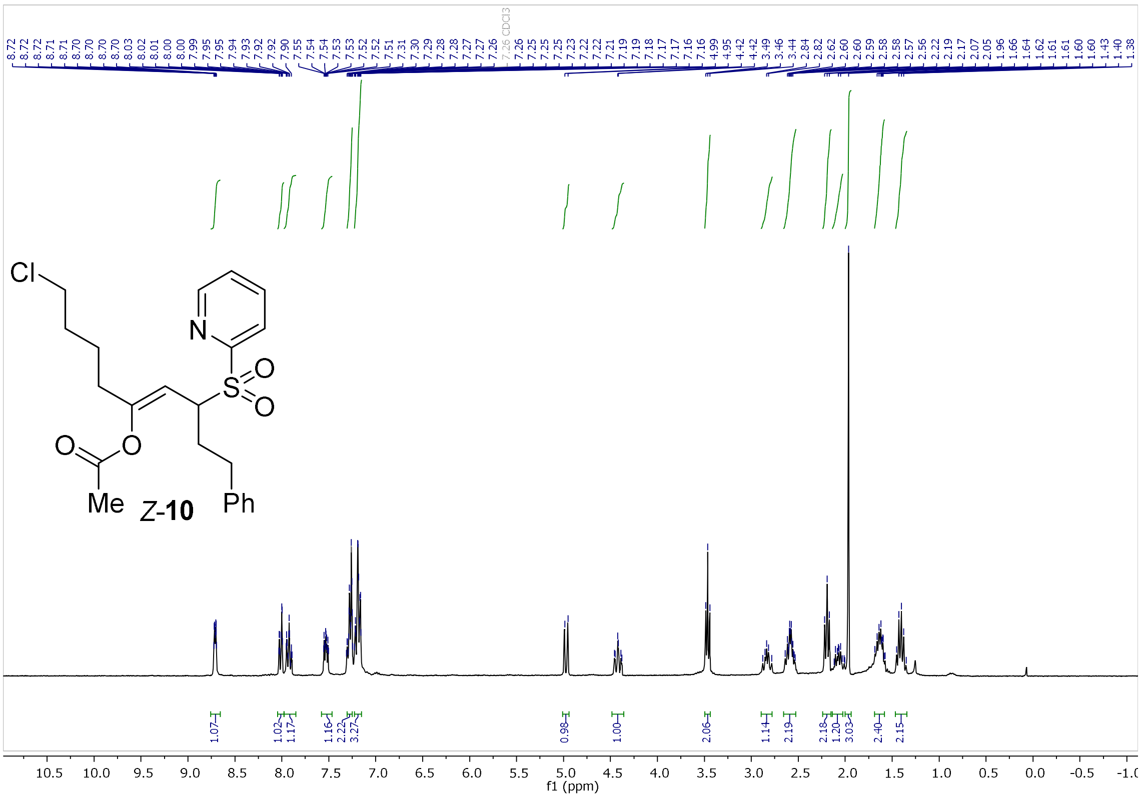

**Figure S21. $^{1}$H NMR spectrum of product *Z*-10.**

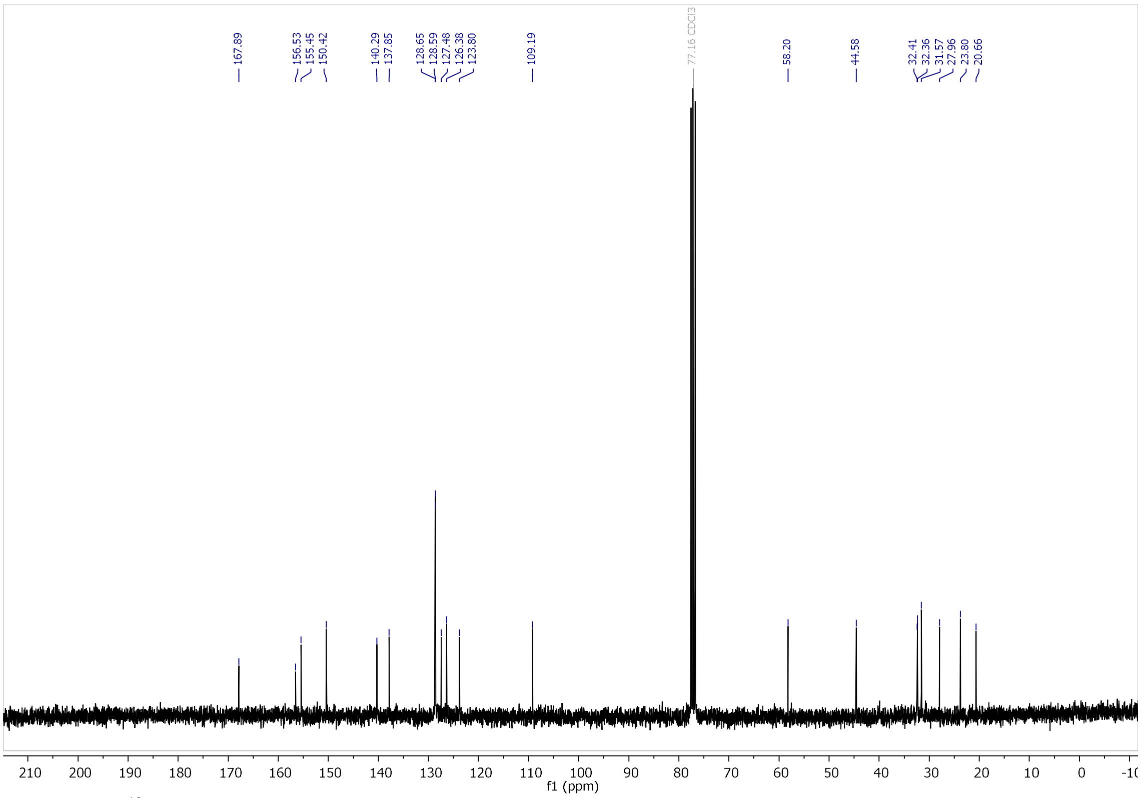

**Figure S22. $^{13}$C NMR spectrum of product *Z*-10.**

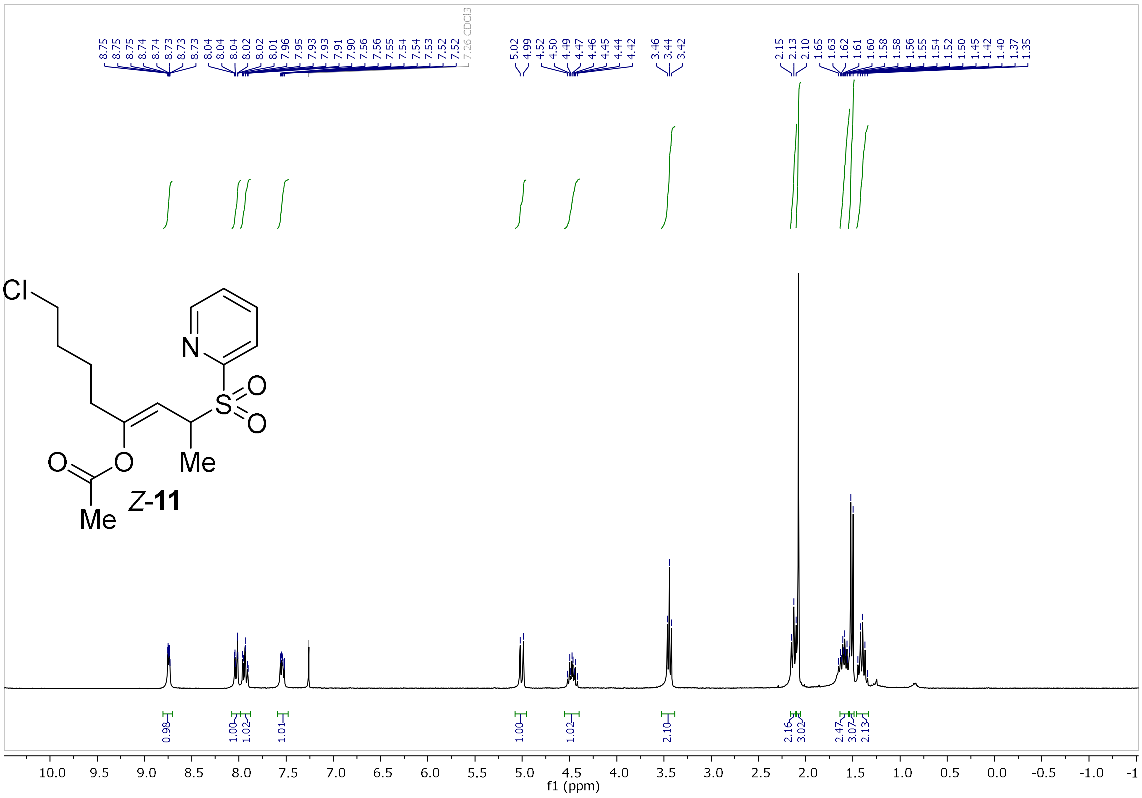


**Figure S23. $^{1}$H NMR spectrum of product *Z*-11.**

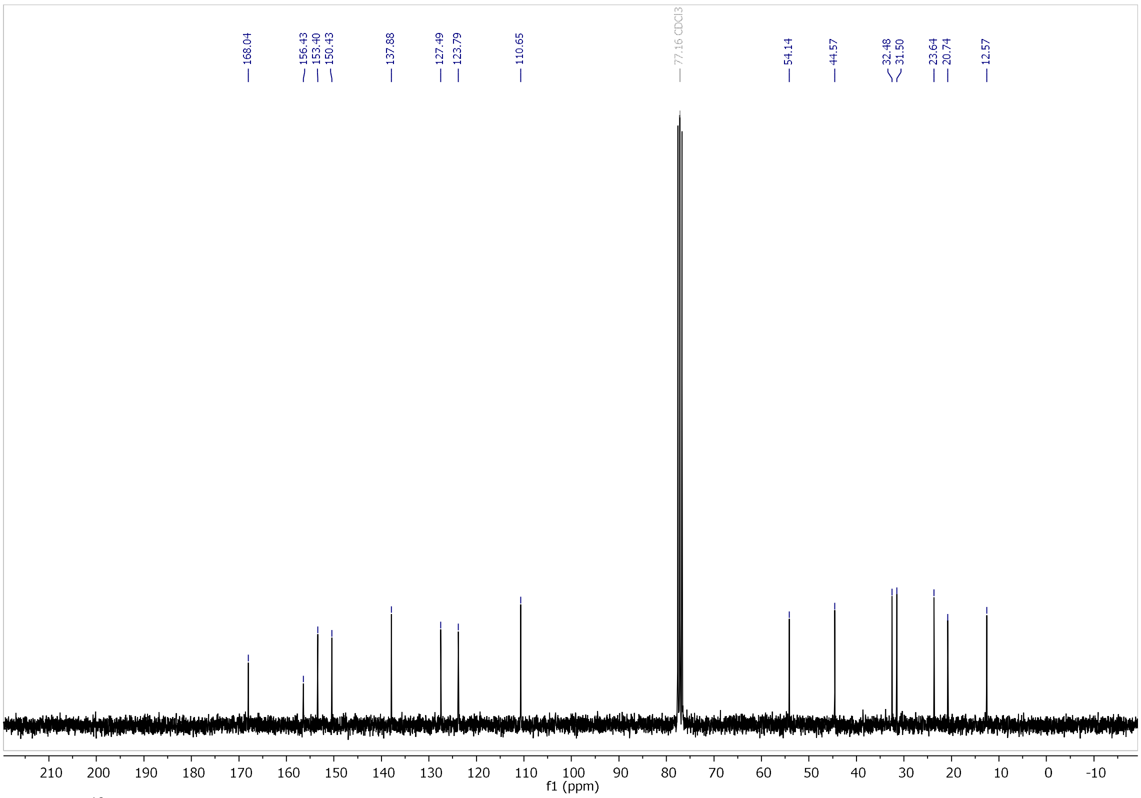


**Figure S24. $^{13}$C NMR spectrum of product *Z*-11.**

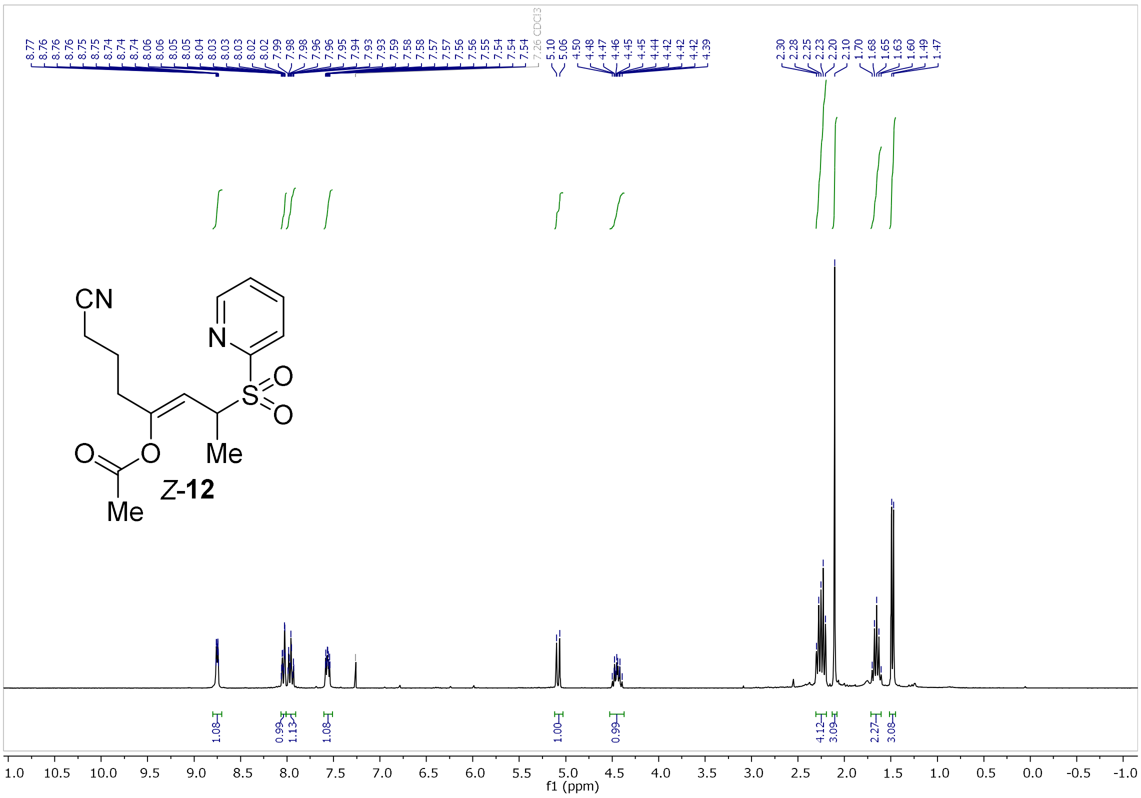

**Figure S25.** $^{1}$**H NMR spectrum of product** ***Z*****-12.**

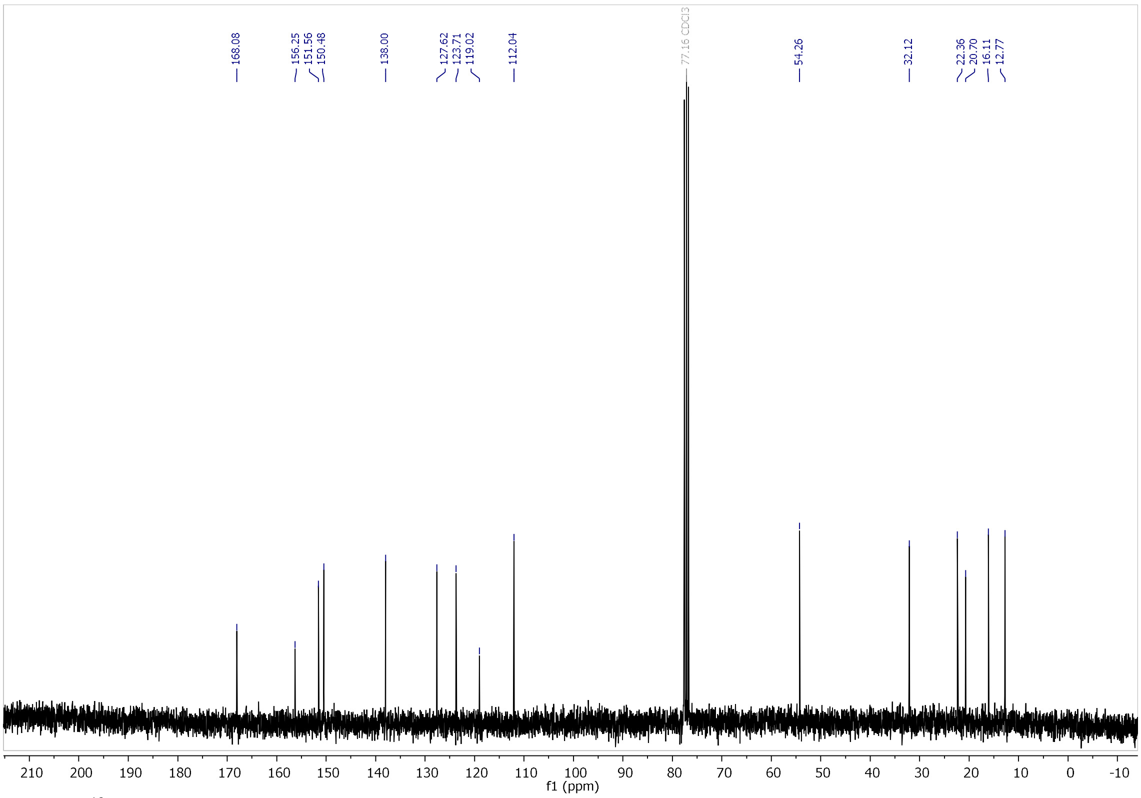

**Figure S26.** $^{13}$**C NMR spectrum of product** ***Z*****-12.**

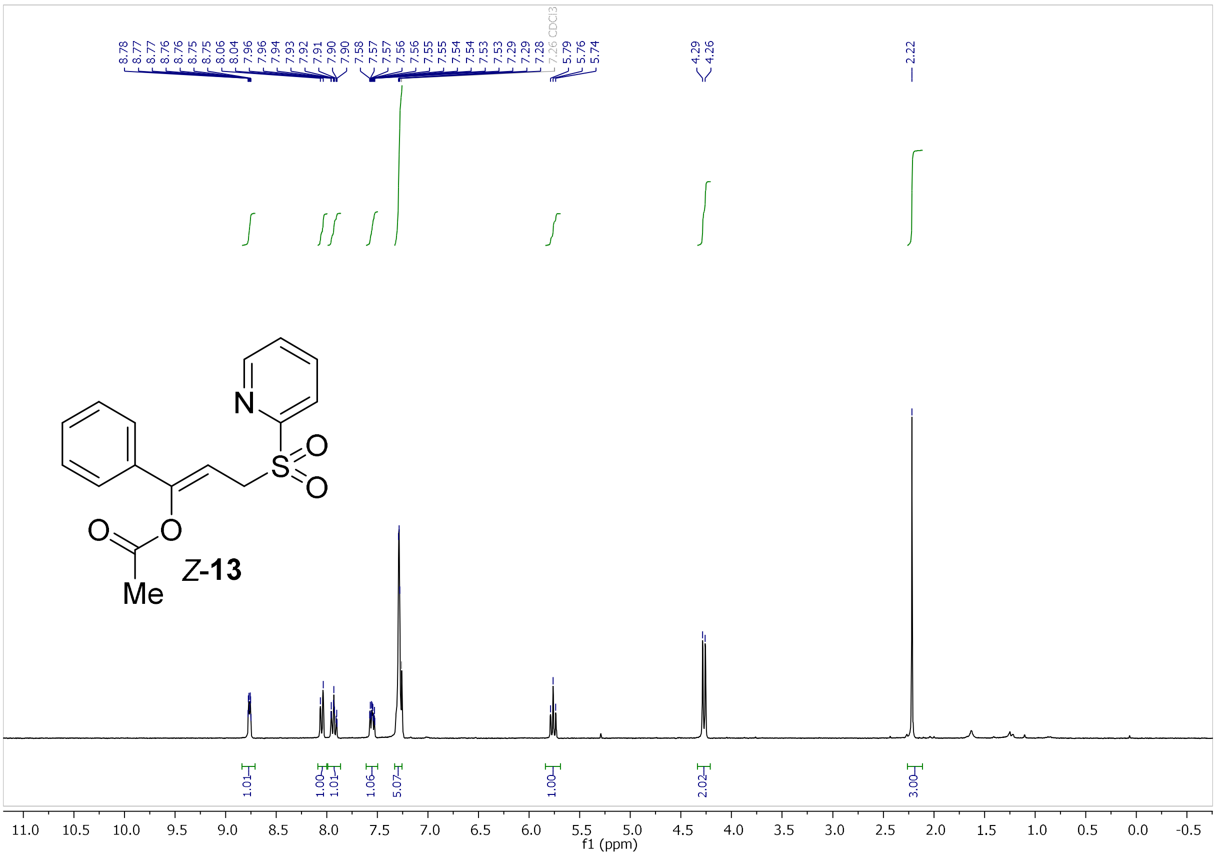


**Figure S27.** $^{1}$**H NMR spectrum of product** ***Z*****-13.**

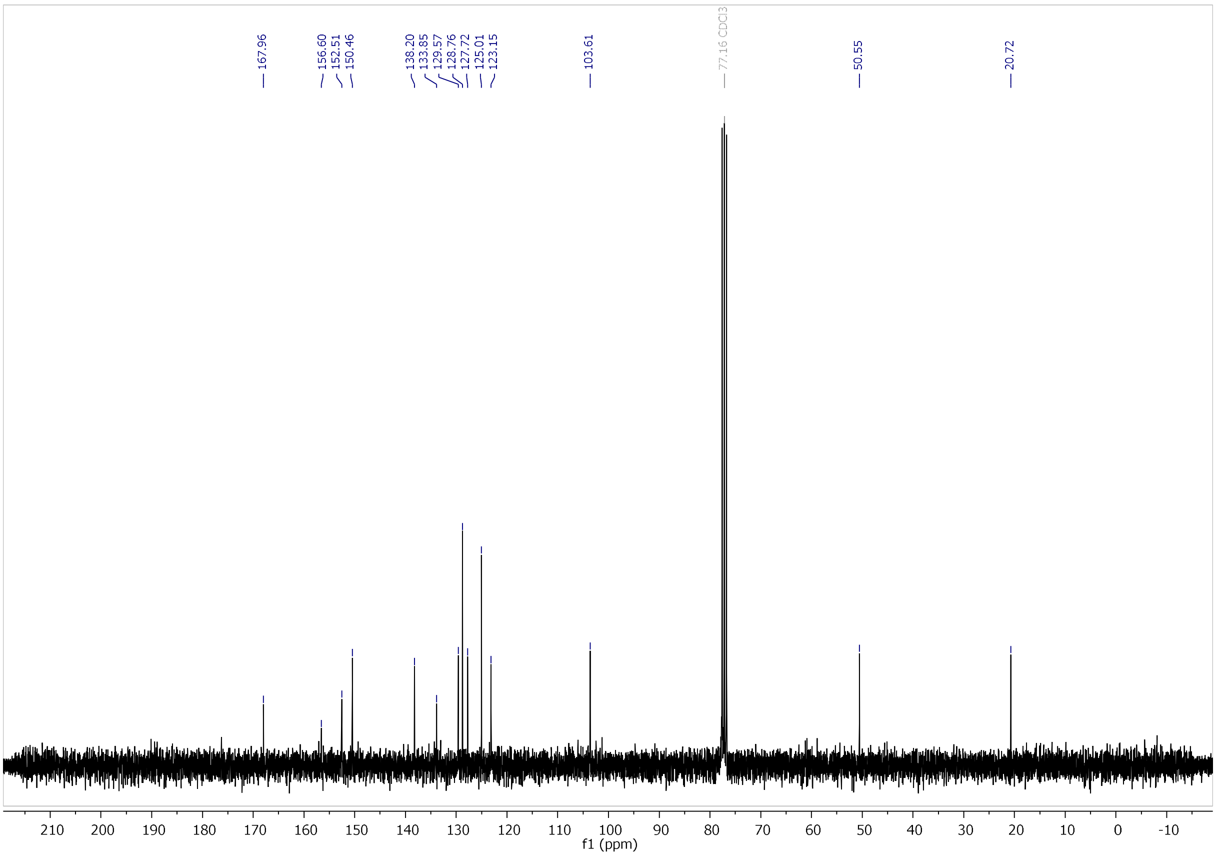


**Figure S28.** $^{13}$**C NMR spectrum of product** ***Z*****-13.**

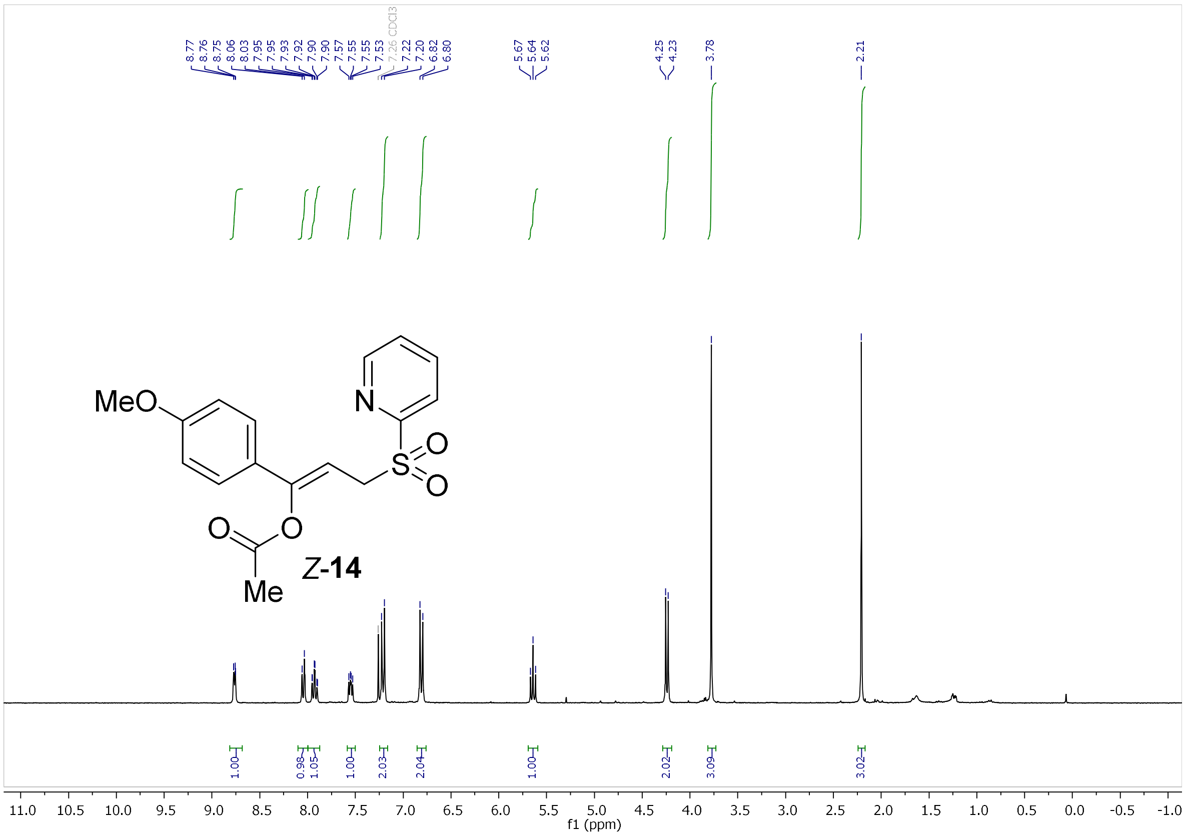


**Figure S29.** $^1$**H NMR spectrum of product** ***Z*****-14.**

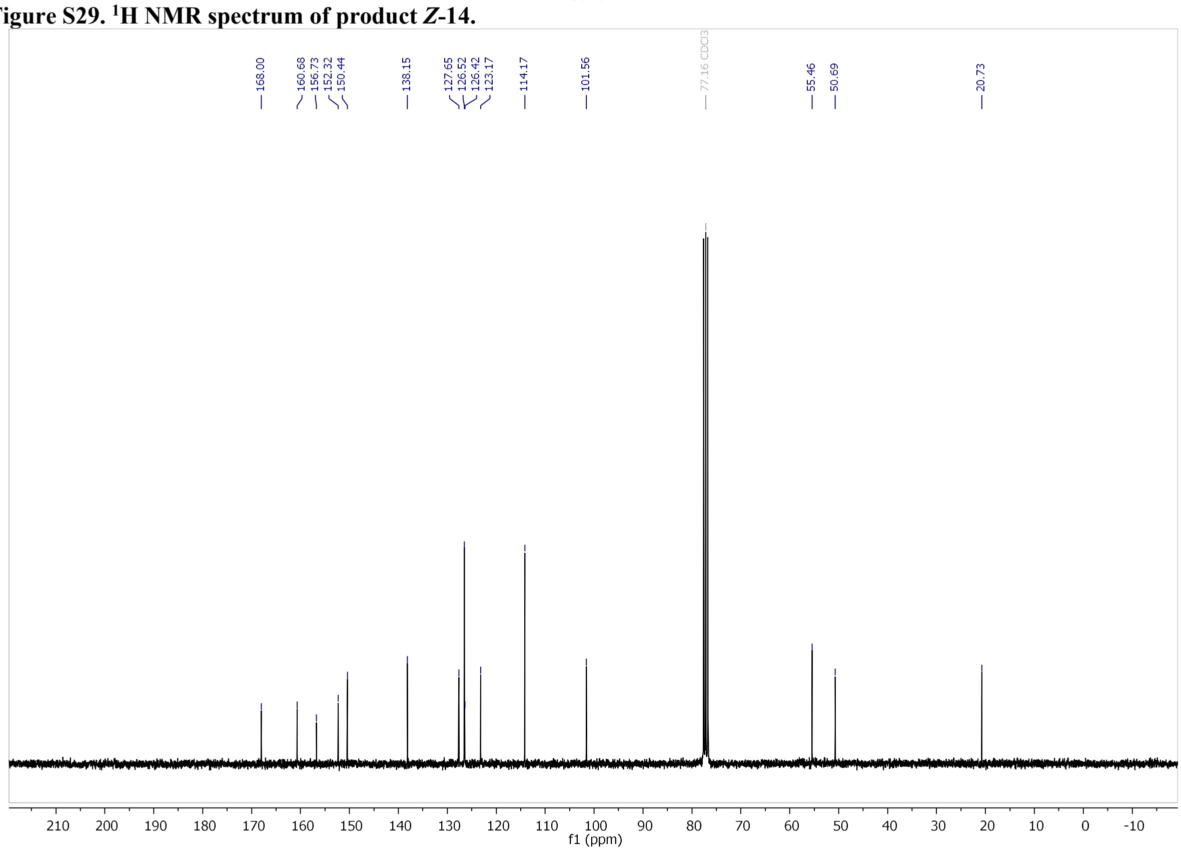


**Figure S30.** $^{13}$**C NMR spectrum of product** ***Z*****-14.**

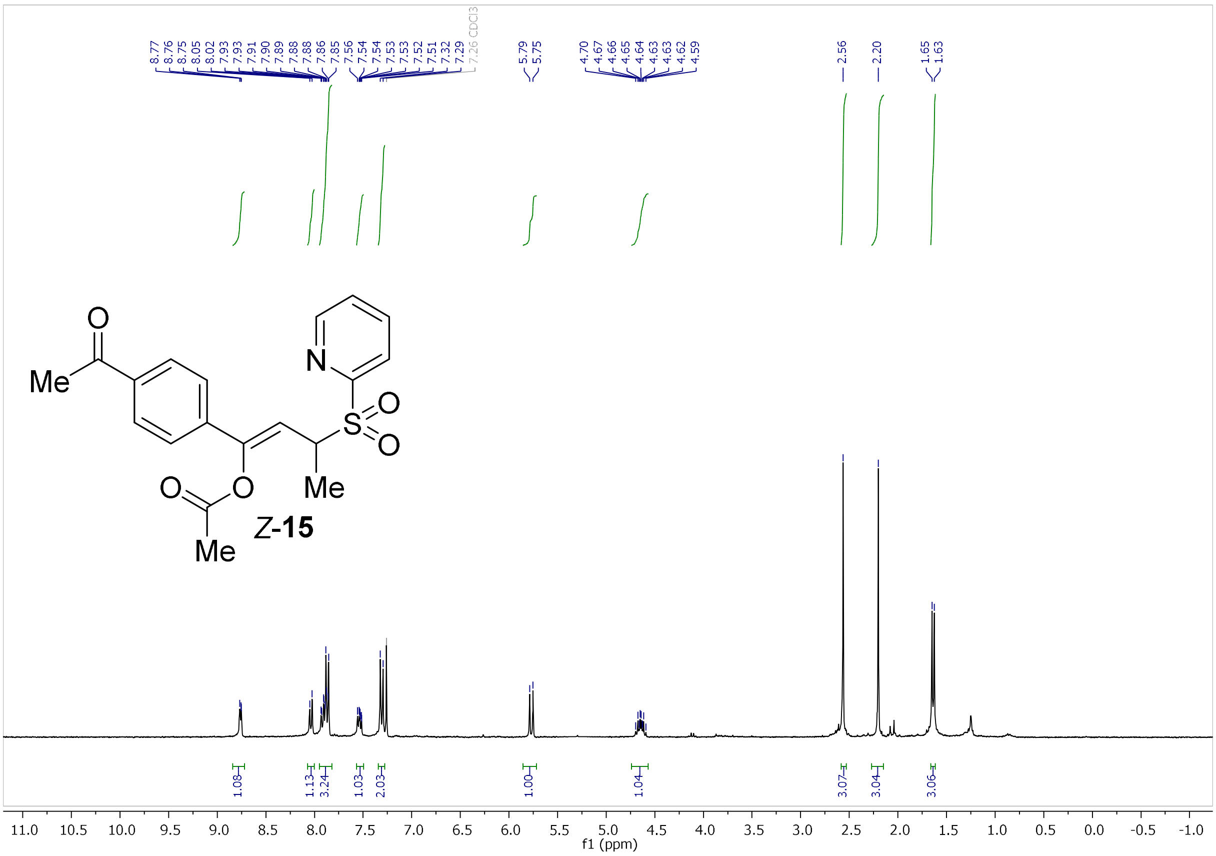

**Figure S31.** **$^{1}$H NMR spectrum of product *Z*-15.**

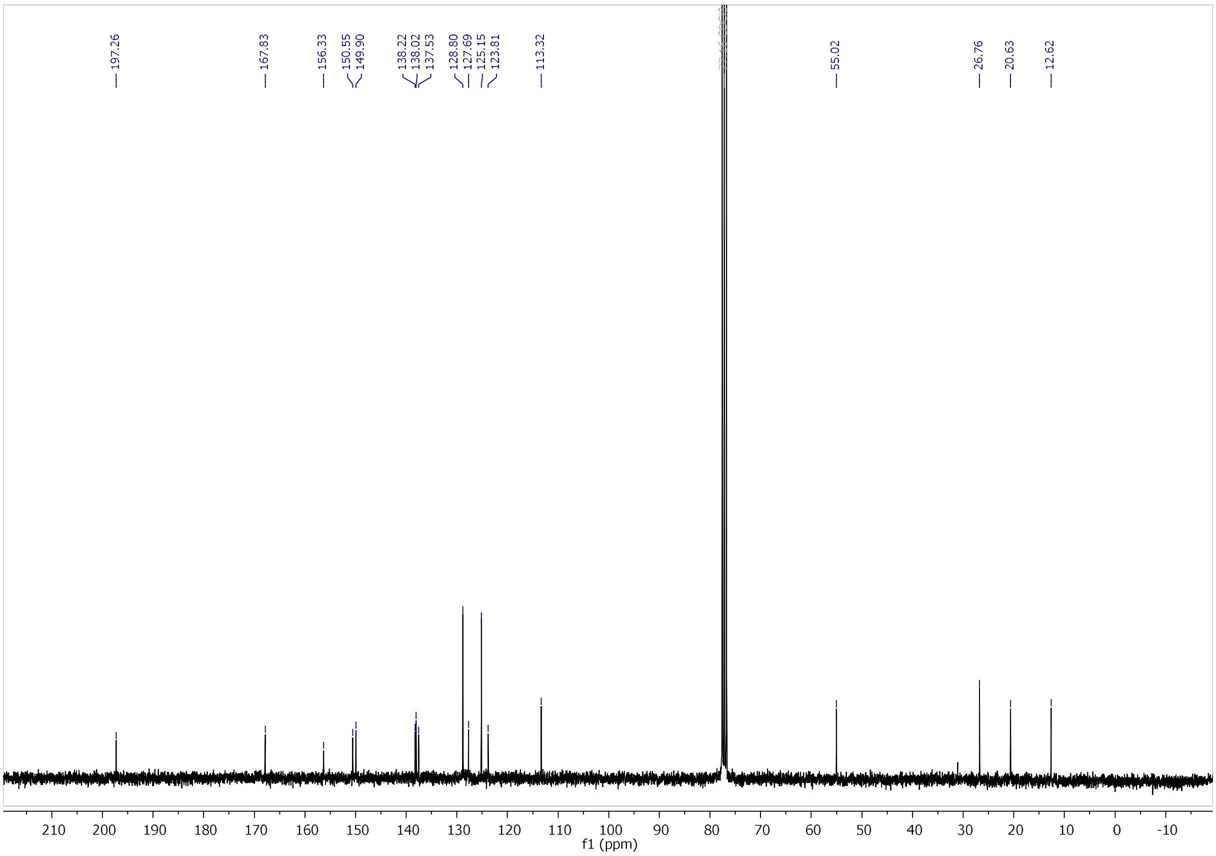

**Figure S32.** **$^{13}$C NMR spectrum of product *Z*-15.**

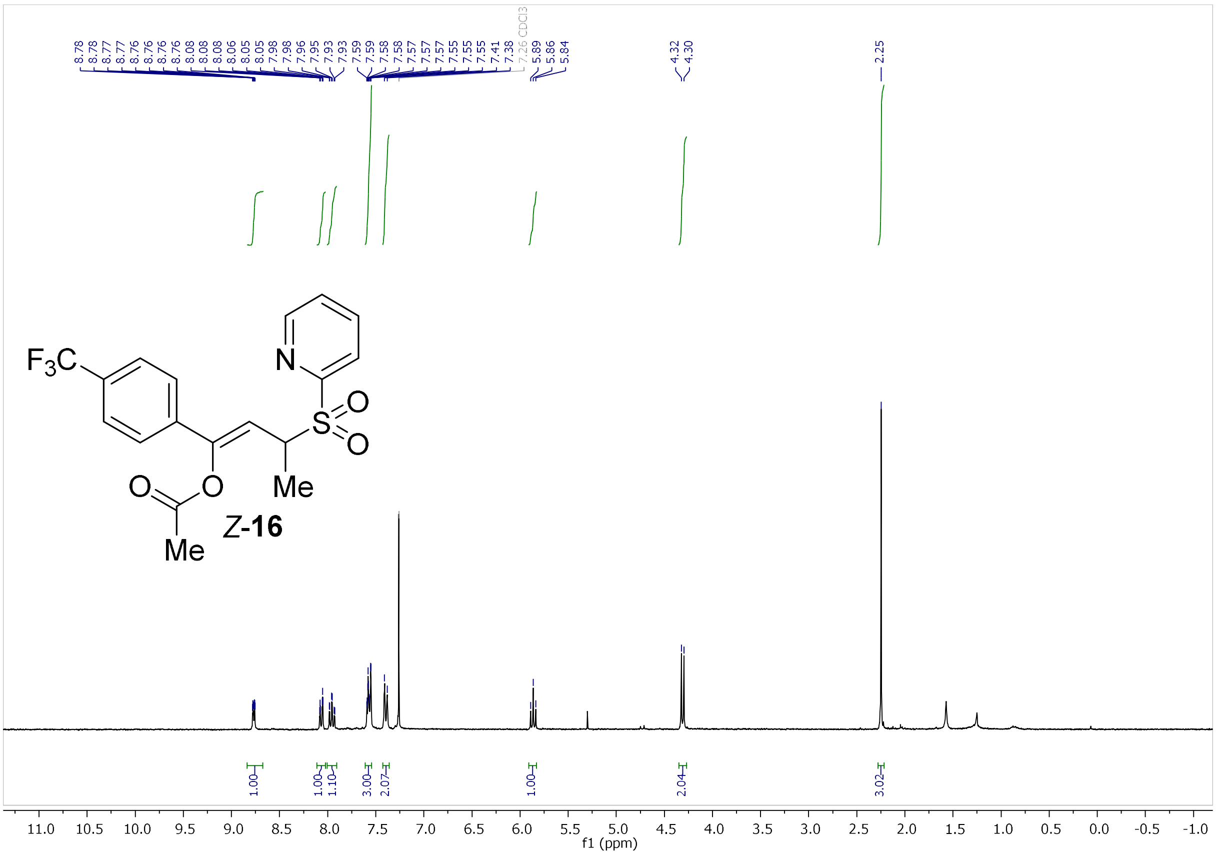


**Figure S33.** **$^{1}$H NMR spectrum of product *Z*-16.**

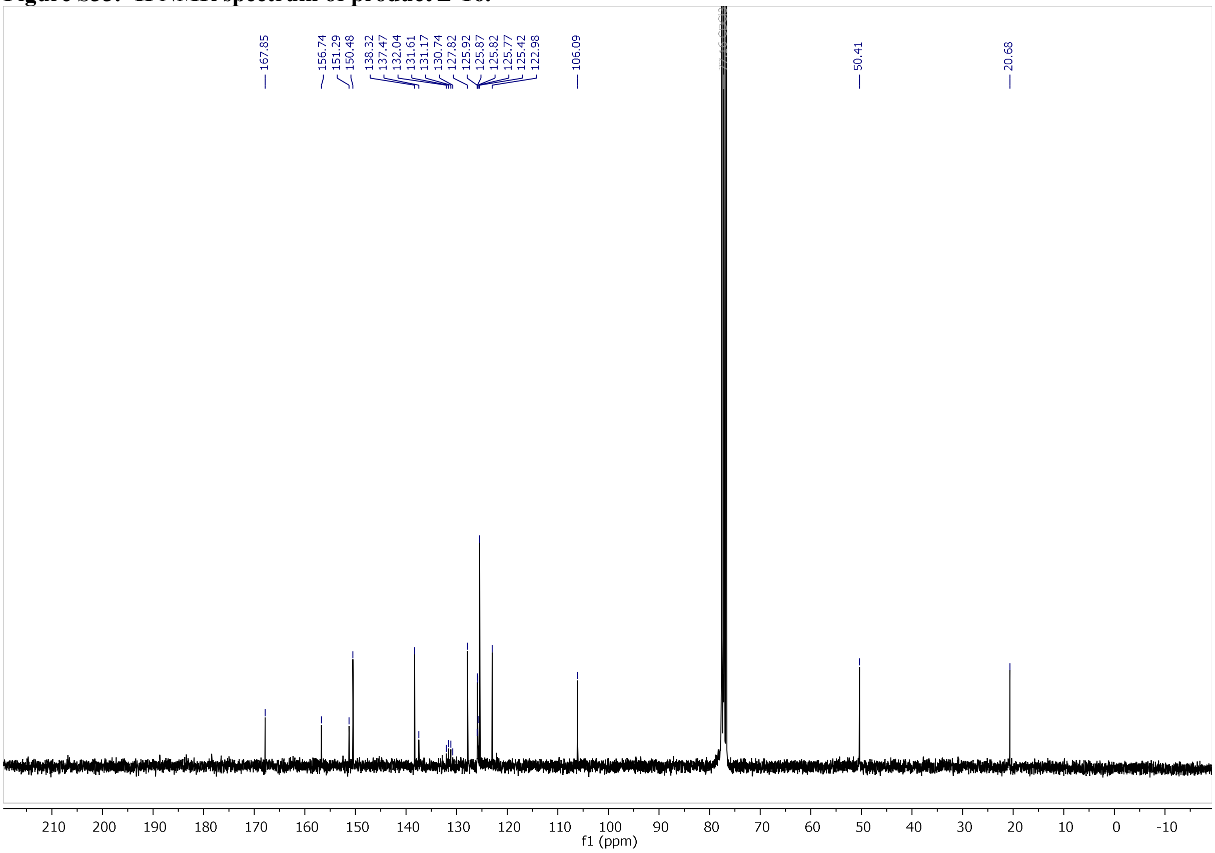


**Figure S34.** **$^{13}$C NMR spectrum of product *Z*-16.**

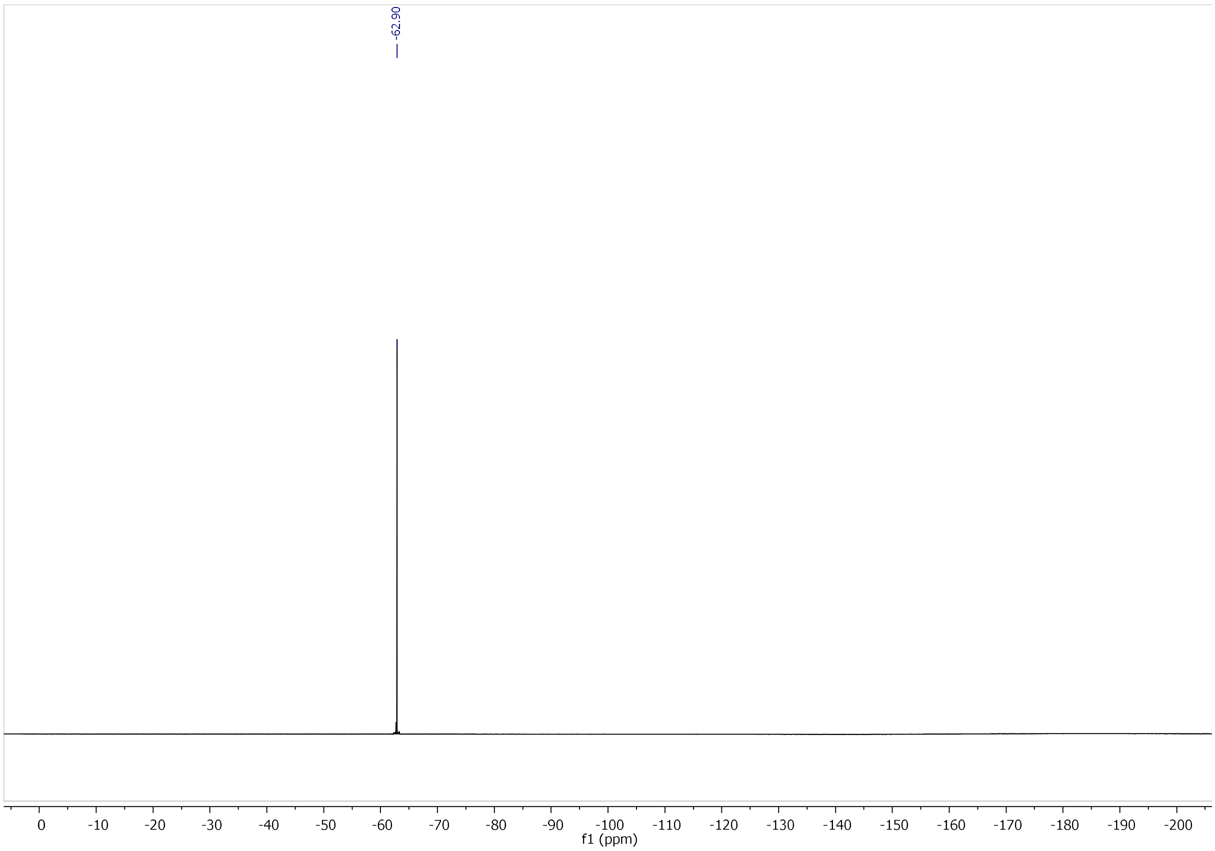

**Figure S35. $^{19}F$ NMR spectrum of product *Z*-16.**
